# Bichromophoric Photosensitizers: How and Where to Attach Pyrene Moieties to Phenanthroline to Generate Copper(I) Complexes

**DOI:** 10.1021/acs.inorgchem.3c00482

**Published:** 2023-05-18

**Authors:** Florian Doettinger, Yingya Yang, Michael Karnahl, Stefanie Tschierlei

**Affiliations:** Department of Energy Conversion, Institute of Physical and Theoretical Chemistry, Technische Universität Brauschweig, Rebenring 31, 38106 Braunschweig, Germany

## Abstract

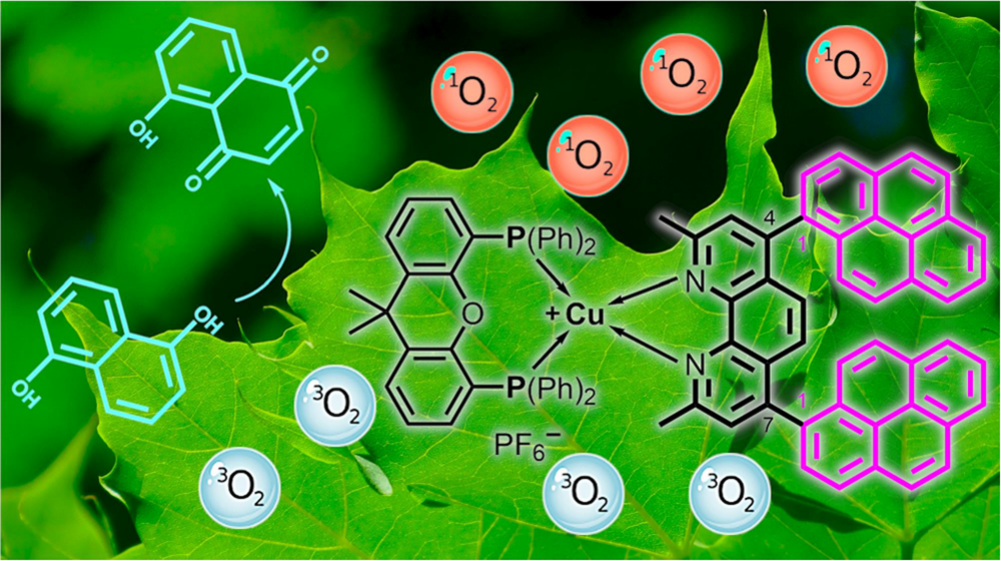

Pyrene is a polycyclic aromatic hydrocarbon and organic dye that can form superior bichromophoric systems when combined with a transition metal-based chromophore. However, little is known about the effect of the type of attachment (*i.e.*, 1- *vs* 2-pyrenyl) and the individual position of the pyrenyl substituents at the ligand. Therefore, a systematic series of three novel diimine ligands and their respective heteroleptic diimine-diphosphine copper(I) complexes has been designed and extensively studied. Special attention was given to two different substitution strategies: (i) attaching pyrene *via* its 1-position, which occurs most frequently in the literature, or *via* its 2-position and (ii) targeting two contrasting substitution patterns at the 1,10-phenanthroline ligand, *i.e.*, the 5,6- and the 4,7-position. In the applied spectroscopic, electrochemical, and theoretical methods (UV/vis, emission, time-resolved luminescence and transient absorption, cyclic voltammetry, density functional theory), it has been shown that the precise choice of the derivatization sites is crucial. Substituting the pyridine rings of phenanthroline in the 4,7-position with the 1-pyrenyl moiety has the strongest impact on the bichromophore. This approach results in the most anodically shifted reduction potential and a drastic increase in the excited state lifetime by more than two orders of magnitude. In addition, it enables the highest singlet oxygen quantum yield of 96% and the most beneficial activity in the photocatalytic oxidation of 1,5-dihydroxy-naphthalene.

## Abstract 2

Merging of a pyrene moiety with a copper(I) based photosensitizer enabled the development of superior bichromophoric systems. It has been demonstrated that the exact position and type of substitution is of crucial importance for the resulting properties and the photocatalytic activity.

## Introduction

The efficient capture and usage of solar energy provides a sustainable alternative to the burning of fossil fuels.^1−5^ In this context, photosensitizers (PS) offer a viable tool for harvesting sunlight and converting solar energy directly into chemical energy. This has been successfully demonstrated for the conversion of small molecules such as the photocatalytic production of hydrogen from water,^6−11^ the photoreduction of CO_2_ to CO,^12−17^ or the generation of reactive singlet oxygen.^18−20^ Furthermore, PS also enable a wide range of organic transformations like the *E* → *Z* isomerization of different alkenes,^20−23^ cyanation^24,25^ or even the challenging alkylation^26,27^ and arylation^28,29^ of various organic substrates.^30−33^

The main goals of current photochemical research are to further improve the catalytic activities, to expand the range of applications, and to better understand structure-activity relations. This includes the design of novel molecular PS with appropriate photophysical properties, such as long-lived excited states, high absorptivity in the visible region, and sufficient (photo)stability.^11,34−38^ Among the different types of PS, especially transition-metal complexes are attracting a great and long-lasting interest due to their largely tunable redox and excited state properties.^11,37−39^ In particular, complexes based on noble and expensive 4d/5d metals such as Pt,^40,41^ Ru,^9,30,42^ and Re^6,43,44^ have been intensively studied. Replacing the metal center in such systems with non-precious and more earth-abundant 3d metals, *e.g.*, Cr,^45−48^ Fe,^49−52^ and Cu^53−59^ has emerged as an attractive step toward large-scale applications.

In this context, especially heteroleptic copper(I) complexes of the type [Cu(N^N)(P^P)]^+^ bearing a diimine and diphosphine ligand enable long-lived excited states, sufficient quantum yields, and high excited state redox potentials.^35,39,55,60−64^ Moreover, they have already successfully demonstrated their competitiveness with noble metal-based Ir(III) and Ru(II) complexes in photocatalysis.^7,10,11,65,66^ As the absorptivity of the heteroleptic Cu(I) complexes in the visible region is rather limited, and with the aim of further improving the excited state properties, several studies have targeted this challenge by modifying the diimine ligand.^33,35,65,67^ One promising strategy is the introduction of extended π-systems in the backbone of the diimine moiety by directly fusing an additional organic chromophore to form large and highly conjugated ligand scaffolds.^39,68−70^ It was proven that the implementation of conjugated groups in this way can strongly increase the excited state lifetimes through the introduction of long-lived ligand-centered triplet (^3^LC) states.^20,69,71,72^ However, this sometimes comes at the cost of significantly shifting reduction potentials toward less negative values and therefore a loss in possible driving force in catalytic transformations.^55,69^

As a result, the proper selection of the coordinating ligands and substituents plays a key role in the design of novel PS. Pyrene and its derivatives, having four fused benzene rings, are promising alternatives, due to their appealing advantages: extensive π-electron delocalization, high attenuation coefficients, long fluorescence lifetimes (τ = 354 ns in toluene), and good ability to transport holes.^73^ Hence, they already attracted a great interest for applications in organic electronics, solar cells, and light-emitting electrochemical materials.^74−77^ Moreover, there are several studies applying pyrenes as substituents for ligand modifications to increase the catalytic activity of the resulting Ir,^71,78−82^ Ru,^7,83,84^ Cr,^47^ Fe,^85^ or Cu^86^ complexes. The incorporation of pyrene substituents into metal-organic chromophores successfully improves the photophysical properties of the complexes by introducing pyrene-based ^3^LC states, which (i) have a quasi-isoenergetic behavior compared to triplet metal-to-ligand charge transfer (^3^MLCT) states and can serve as an energy reservoir for excited states;^83,84,87,88^ (ii) or act as the lowest-lying excited state with long excited state lifetime.^71,72^

**Scheme 1:**
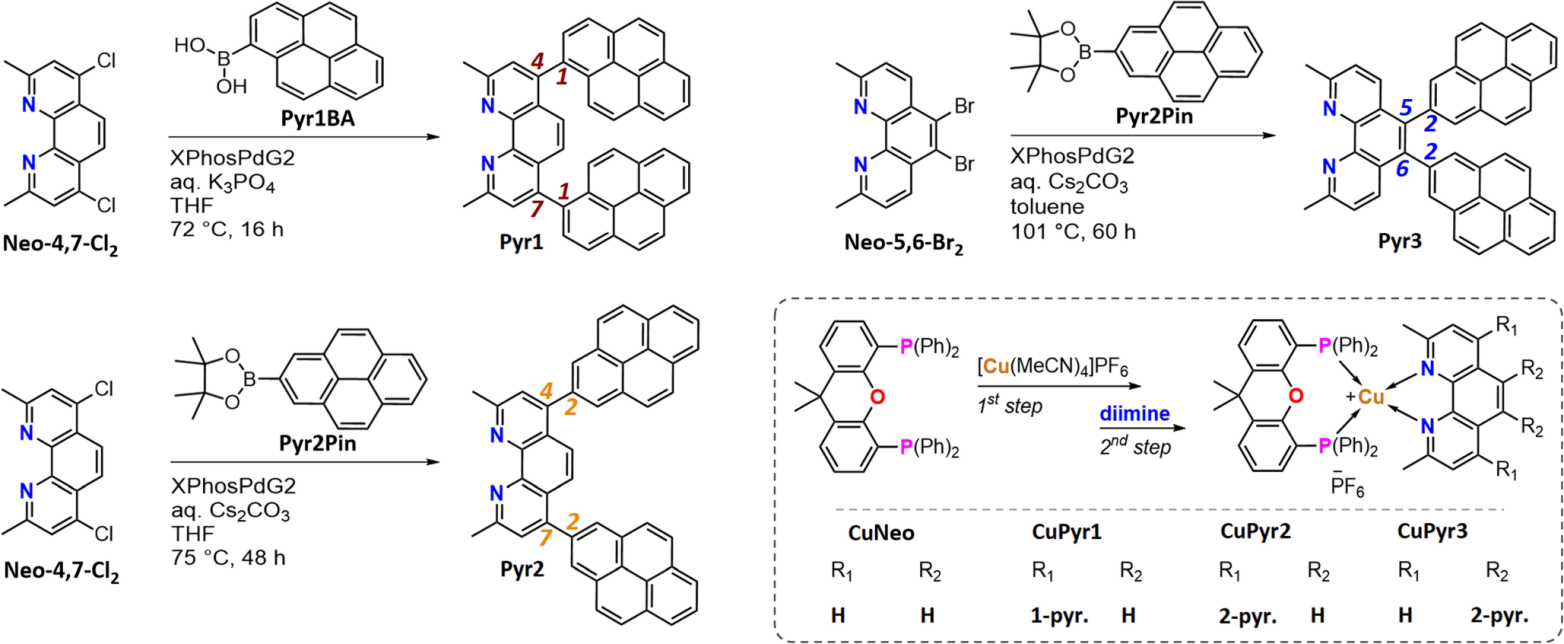
Representation of the General Synthetic Procedure Leading to the Different Types of Pyrene-Substituted Phenanthroline Ligands and Their Heteroleptic Copper(I) Complexes Discussed within This Work

There are two different options to attach a pyrene substituent to a potential ligand, *i.e.*, in the 1-position and in the 2-position (Scheme 1). Studies indicated that the 1-position of pyrene is markedly more active than other positions.^89^ In strong contrast, the 2-position is much more difficult to directly be substituted because the nodal planes of the highest occupied molecular orbital (HOMO) and the lowest unoccupied molecular orbital (LUMO) pass through it.^90,91^ The direct comparison between a 1- and 2-pyrene substituted deoxyuridine nucleoside illustrated that the different positions of pyrene can alter the optical properties. This is due to the fact that 1-pyrenyl substitution causes a stronger electronic coupling between the two aromatic parts relative to that at the 2-position.^87^ Nevertheless, as a rigid and linear linker, the particular long axis along 2- and 7-positions makes pyrene attractive for the synthesis of metal–organic frameworks (MOFs) and covalent organic frameworks (COFs).^92,93^

To address this issue in more depth, this study has examined two different effects: (i) the introduction of two pyrene substituents at two different positions (*i.e.*, 1- vs 2-substitution) and (ii) of two different positions at the 1,10-phenanthroline itself (*i.e.*, 4,7- vs 5,6-position) (Scheme 1). By comparing the resulting three novel ligands and their corresponding copper(I) complexes, the impact of the substitution positions of pyrene and phenanthroline on the photophysical and photocatalytic properties was explored in detail. Moreover, these properties were compared with those of the unsubstituted reference complex **CuNeo**. Cyclic and differential pulse voltammetry, various steady-state and time-resolved spectroscopic techniques, as well as time-dependent density functional theory (TD-DFT) were used to investigate this novel class of heteroleptic copper(I) PS. Finally, all three complexes were successfully applied in the photocatalytic generation of reactive singlet oxygen (^1^O_2_) over several cycles. In addition, the formed ^1^O_2_ was also used for the catalytic photooxidation of 1,5-dihydroxynaphthalene (DHN) to Juglone^94−96^ to examine their catalytic potential and to study structure-activity relationships.

## Results and Discussion

### Synthesis and Structural Characterization

#### Ligand Synthesis (**Pyr1–3)**

The novel diimine ligands **Pyr1**, **Pyr2**, and **Pyr3** were synthesized *via* Suzuki–Miyaura cross-coupling reactions from their respective pyreneboronic acid (and its pinacol esters) utilizing a XPhos-Pd-G2 catalyst.^97^ The required cross-coupling substrates 4,7-dichloro-2,9-dimethyl-1,10-phenanthroline (**Neo-4,7-Cl_2_**)^98^ and 5,6-dibromo-2,9-dimethyl-1,10-phenanthroline (**Neo-5,6-Br_2_,** see Scheme 1)^36^ were synthesized on gram-scale following literature-known procedures.

Subsequent cross-coupling reactions toward the desired ligands **Pyr1**–**3** varied strongly due to the different reactivity of the coupling substrates and reagents. **Pyr1** was synthesized from commercially purchased pyrene-1-boronic acid (**Pyr1BA**) and **Neo-4,7-Cl_2_** in a biphasic mixture of THF and an aqueous 0.5 M K_3_PO_4_ solution, similar to our previously described approach^36^ (see SI Chapter 2 for details). Adjustments had to be made to the work-up procedure due to the polarity and solubility of **Pyr1**, **Pyr1BA**, and the byproduct pyrene: column chromatography was conducted first on basic aluminum oxide to remove the remains of the catalyst and boronic acid. Further chromatography on silica was necessary to remove pyrene as the remaining impurity. Due to the extended work-up procedure, the isolated yield was 55%, although quantitative conversion can be assumed as only the 4,7-disubstituted ligand was isolated from the reaction.

**Pyr2** was synthesized from pyren-2-boronic acid pinacol ester (**Pyr2Pin**), which was prepared directly from pyrene in an Ir-catalyzed C–H activation reaction with bis(pinacolato)diboron according to a known procedure (see SI Chapter 2 for further details).^99^ More importantly, under basic conditions, the reacting species **Pyr2BA** is slowly released from **Pyr2Pin**,^100^ significantly suppressing an assumed deboronation side reaction. **Neo-4,7-Cl_2_** was therefore reacted with **Pyr2Pin** under similar conditions as **Pyr1**. To ensure a constant release of the reactive boronic acid from its pinacol ester, an aqueous 1 M Cs_2_CO_3_ solution was chosen, as an increased solubility in the organic solvent was expected. The reaction was prolonged by a factor of three (48 h vs 16 h). After column chromatography, the product could be recrystallized from toluene in a final isolated yield of 49%.

**Pyr3** was synthesized from **Pyr2Pin** and **Neo-5,6-Br_2_** in a biphasic mixture of water with Cs_2_CO_3_ as the base. When using THF at reflux temperature as described for **Pyr1** and **Pyr2**, the reaction also yields the mono-substituted phenanthroline derivative as a side product, which could not be separated from the crude mixture. Applying the more classical [Ph(PPh_3_)_4_] catalyst expectedly yielded unreacted substrates as the main component after the reaction, highlighting the need for the more reactive XPhos-Pd-G2 catalyst^36^ and more harsh conditions. Therefore, THF was replaced by toluene, which allowed higher temperatures, and the reaction time was increased to 60 h to achieve full conversion without producing the mono substituted byproduct. Owing to its low solubility and unfavorable crystallization behavior, **Pyr3** was purified by preparing its crude homoleptic complex as an intermediate (SI Chapter 2). The ligand **Pyr3** was finally obtained after liberation from the complex using potassium cyanide (please see SI Chapter 2 for details concerning safety) in an isolated yield of 54%.

#### Complex Synthesis

The syntheses of the heteroleptic copper(I) PS **CuPyr1**, **CuPyr2**, and **CuPyr3** were conducted following a well-known one-pot two-step procedure (Scheme 1) starting from [Cu(MeCN)_4_]PF_6_ (MeCN = acetonitrile).^7,20,34,36,69,70^ To ensure a slow and precise addition of the diimine ligand to the reaction mixture, an automatic syringe pump was utilized for the preparation of **CuPyr1** and **CuPyr3**. In contrast, due to its low solubility in CH_2_Cl_2_, **Pyr2** was added directly as a solid under inert conditions at −20 °C for the synthesis of **CuPyr2**.

Contrary to earlier studies,^7,20,34,36,69,70^ the novel copper(I) complexes could not be precipitated as solids by adding *n*-hexane. Instead, the compounds form a viscous deep red oil even at −20 °C, possibly due to competing intermolecular π–π interactions induced by the pyrenyl substituents.^101^ After decanting the remaining solution and treating the oil with an excess of *n*-hexane in an ultrasonic bath, the compounds were carefully dried to obtain a solid. Isolated yields for **CuPyr1**, **CuPyr2**, and **CuPyr3** were 71, 41, and 61%, respectively.

The compounds were then fully characterized by nuclear magnetic resonance (NMR) spectroscopy (^1^H, ^13^C, ^31^P) and high-resolution mass spectrometry (HRMS) (SI Chapters 2, 3, and 4). Attempts to grow single crystals for X-ray crystallography with various methods did not result in any crystal formation so far.

### Molecular Structure Analysis

**Figure 1:**
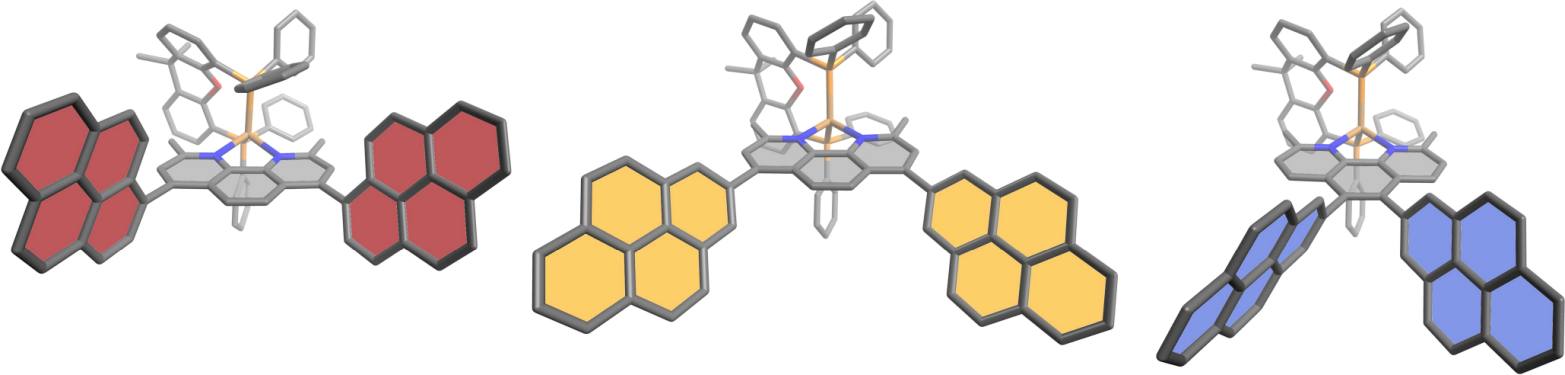
Molecular structures of the complexes **CuPyr1**, **CuPyr2**, and **CuPyr3** (left to right) predicted by DFT calculations simulated in acetonitrile (PBE0-D3(BJ)/def2-tzvp, CPCM). **CuPyr1** bears pyren-1-yl substituents in the 4,7-position, **CuPyr2** bears pyren-2-yl substitution in the 4,7-position, and **CuPyr3** bears pyren-2-yl substitution in the 5,6-position.

As the growth of single crystals was not successful, the structural analysis is based on theoretically predicted structures obtained by TD-DFT calculations (see Figure 1). Selected parameters of **CuPyr1–3** and the reference **CuNeo** are gathered in Table 1, along with experimental results for **CuNeo** for comparison.

**Table 1:** Selected Bond Lengths (pm), Pyrene-to-Pyrene Distances Pyr–Pyr (pm), Bite Angles (°), and Torsion Angles between the Substituent and Phenanthroline τ_Sub_ (°) of the Complexes **CuPyr1–3** and **CuNeo** as Predicted from DFT Calculations^a^^,^^b^

	**CuPyr1**	**CuPyr2**	**CuPyr3**	**CuNeo**	**CuNeo**(exp)^c^
Cu–P	226.8	226.1	226.4	226.3	226.26(11)
	229.4	229.1	231.1	229.1	228.63(13)
Cu–N	210.8	209.4	211.5	211.0	208.4(3)
	211.1	211.4	212.1	211.8	211.2(3)
C–C	148.0	147.4	148.3		
	148.1	147.6	148.3		
Pyr–Pyr	1070.1	1231.1	636.0		
N–Cu–N	79.3	79.4	79.3	79.8	80.53(13)
P–Cu–P	117.3	119.2	118.3	118.8	112.93(4)^d^
τ_Sub_GS	84.7	–50.3	72.8		
	71.1	51.6	69.0		
τ_Sub_ES	60.1	–49.5	68.2		
	64.9	41.8	68.1		
Δτ_Sub_(GS-ES)	24.6	0.8	4.6		
	6.2	9.8	1.0		

a Torsion angles τ_Sub_ are given for the electronic ground state (GS) and optimized lowest triplet state (ES) including the corresponding differences Δτ_Sub_. Experimental crystallographic data for **CuNeo** are also presented for comparison (see **CuNeo**(exp), taken from ref (39)).

b Values given in parentheses for **CuNeo**(exp) are the estimated standard deviation of the measurement.

c Published earlier by ref (39).

d Please note that the calculated P–Cu–P bite angles fit well within the range of experimentally determined angles of closely related compounds.^36^

The Cu–P and Cu–N bonds lengths as well as the N–Cu–N and P–Cu–P angles are well described by theory and are mostly uniform within the computed structures. C–C bond lengths of the bond between the pyrenyl substituent and the phenanthroline moiety do also not differ significantly. They are predicted to be slightly shorter when compared to a related phenyl substituted complex (149.(5) pm in [Cu(2,9-dimethyl-4,7-diphenyl-1,10-phenanthroline)(xantphos)]^+^.^102^

It is noteworthy that the average distances between the two pyrene substituents vary between 636 pm (**CuPyr3**) and 1231 pm (for **CuPyr2** measured between the centers of each substituent), indicating a possible stronger pyrene–pyrene interaction for **CuPyr3** with the substituents in the 5,6-position. This can include, for example, interactions *via* intramolecular π–π interactions in the ground state or in an excited state.

Significant differences can also be found when comparing the torsion angles around the new C–C bond τ_Sub_GS in the singlet ground state. Here, **CuPyr2** tends to exhibit a more coplanar structure (−50°) compared to **CuPyr1** and **CuPyr3** (85° and 73°, respectively), most likely due to less steric hindrance. Considering the same parameter but in the lowest triplet state τ_Sub_ES, the difference Δτ_Sub_ between these angles (*i.e*., Δτ_Sub_ = τ_Sub_GS – τ_Sub_ES) is the largest for **CuPyr1** and the smallest for **CuPyr3**. This suggests that especially in **CuPyr1**, a possible triplet state may be partially delocalized over the phenanthroline and the pyrenyl moiety, which could be due to the increased orbital overlap. In contrast, **CuPyr3** is therefore suggested to have the weakest orbital overlap. However, the small difference could also be due to greater repulsion between the two pyrenyl-substituents and/or possible intramolecular π–π interactions preventing stronger rotation.

### Electrochemical Studies

**Figure 2:**
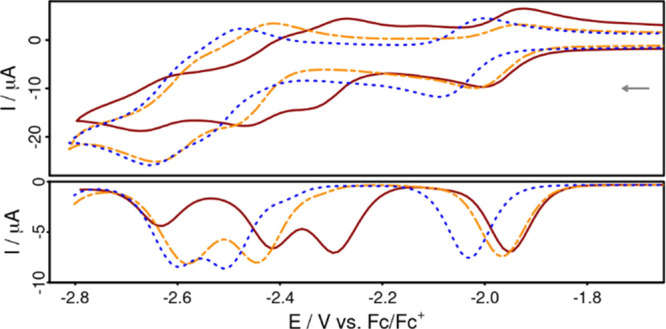
Cyclic voltammograms (top) and differential pulse voltammograms (bottom) of **CuPyr1** (red, solid), **CuPyr2** (orange, dashed), and **CuPyr3** (blue, dotted) in dimethylformamide. Conditions: complex concentration of 1 mM, scan rate of 100 mV s^–1^, 0.1 M [Bu_4_N][PF_6_] as the supporting electrolyte.

The electrochemical properties of **CuPyr1–3** and the corresponding ligands **Pyr1–3**, as well as of pyrene as a reference, were determined by cyclic and differential pulse voltammetry (CV and DPV, Figures 2 and S30–S34, Table 2) in dimethylformamide solution containing 0.1 M [Bu_4_N][PF_6_] as the electrolyte. Based on scan-rate-dependent CV measurements (Figure S34), **CuPyr1–3** show three reversible cathodic events. Compared to the only singly reducible **CuNeo**, the first reversible reduction waves of **CuPyr1**, **CuPyr2**, and **CuPyr3** (−1.97, −2.02, and −2.10 V, respectively) can be assigned to the reduction of the phenanthroline moiety. Interestingly, the first reduction potential of **CuPyr3** is identical with that of the reference **CuNeo** (both −2.10 V), which lacks pyrene substituents. Contrarily, the reduction waves of **CuPyr1** and **CuPyr2** are shifted anodically by 130 and 80 mV, respectively. This indicates that the pyrenyl substitutions at the 4,7-position of the phenanthroline have a greater impact on the electrochemical behavior than those at the 5,6-position.

**Table 2:** Summary of the Photophysical Properties of all Pyrene-Based Ligands (**Pyr1–3**), Complexes (**CuPyr1–3**), and of Selected Reference Compounds (Pyrene, **CuNeo**) in Acetonitrile and Dichloromethane Solution as well as the Electrochemical Data in Deaerated Dimethylformamide Referenced vs the Ferrocene/Ferricenium (Fc/Fc^+^) Couple^a^

	λ_abs_ [nm]	λ_em_ [nm]^b^		τ_em_^c^ [ns]	τ_excited state_^c^ [μs]		ϕ_1O2_^d^	*E*_1/2,red_ [V]
	aerated MeCN (ε [10^3^ M^–1^ cm^–1^])	MeCN (ϕ)	CH_2_Cl_2_ (ϕ)	MeCN (aerated MeCN)	MeCN	CH_2_Cl_2_	aerated MeCN	
pyrene	335 (48.4)	371 (0.62)^f^	371 (0.38)^f^	e	e	1.23^f^	e	–2.58
**CuNeo**	378 (3.1)^f^	564^f^ (0.86)^f^	560	204^f^ (64)^f^	0.20	4.12	0.20	–2.10^f^
**Pyr1**	344 (32.98)	430 (0.69)	420, 494 (0.50)	<10 (<10)	16.03	26.43	e	–2.30
								–2.49
								–2.51
**CuPry1**	343 (46.05), 375 (16.38)	431 (0.32)	400, 500 (0.01)	<10 (<10)	22.42	35.95	0.96	–1.97
								–2.32
								–2.42
								–2.64^g^
**Pyr2**	340 (58.51)	416 (0.13)	405 (0.17)	28.6 (11.8)	3.71	9.86	e	–2.60
**CuPyr2**	340(62.48), 377(3.13)	415 (0.09)	400, 505 (<0.01)	30.3 (12.2)	17.70	25.02	0.83	–2.02
								–2.46
								–2.54
**Pyr3**	326 (34.47)	400, 480 (0.03)	398, 498 (0.03)	29.7 (11.2)	8.47	h	e	–2.59
**CuPyr3**	327 (65.74), 375 (5.96)	484 (0.001)	400, 515 (<0.01)	11.0 (<10)	1.15, 5.81	1.19, 2.62	0.66	–2.10, −2.54, −2.67

a All data were obtained under inert conditions at room temperature unless otherwise noted.

b Excited at 334 nm.

c Excited at 355 nm.

d Excited at 407 nm.

e Not determined.

f Data were taken from refs (36, 39, 104−106).

g Irreversible.

h Signal with a lot of noise because the sample is not stable.

A comparison with uncoordinated pyrene (−2.58 V) reveals that the second and third reversible reductions in **CuPyr1–3** can be attributed to the reduction of both pyrenyl substituents (−2.32/–2.42, −2.46/–2.54, and −2.54/–2.67 V, see Table 2). Only **CuPyr1** has a fourth reduction, which might indicate an even doubly reduced pyrenyl substituent. Furthermore, a maximum difference of ∼200 mV was observed between these sets of reductions for **CuPyr1–3**, with **CuPyr1** being the easiest (−2.32 V) and **CuPyr3** being the most difficult (−2.54 V) to reduce. These findings suggest that the electronic communication between the substituent and the phenanthroline is most pronounced in the pyrene-1-yl-substituted **CuPyr1**. In contrast, pyrene-2-yl substitution in the 5,6-position (**CuPyr3**) is expected to induce barely any electronic interaction as the phenanthroline- and pyrene-based reductions are almost identical to those of the two unsubstituted building blocks (*i.e.*, phenanthroline and pyrene).

Comparing the ligands with their complexes, potential differences of 123, 90, and 40 mV were found for the reduction of the pyrenyl substituents for **CuPyr1**, **CuPyr2**, and **CuPyr3**, respectively. The fact that the largest potential difference is observed for **CuPyr1** is again in line with the argument that the strongest electronic interaction between phenanthroline and the pyrene moieties is present in the 1-pyrenyl system.

The three complexes **CuPyr1–3** show an irreversible oxidation event, which can be attributed to the literature-known oxidation of the Cu–P bond at about +0.9 V (Figures S31 and 32)^39,69^ and to the irreversible oxidation of pyrenes at similar potentials.^85^

### Photophysical Properties

**Figure 3:**
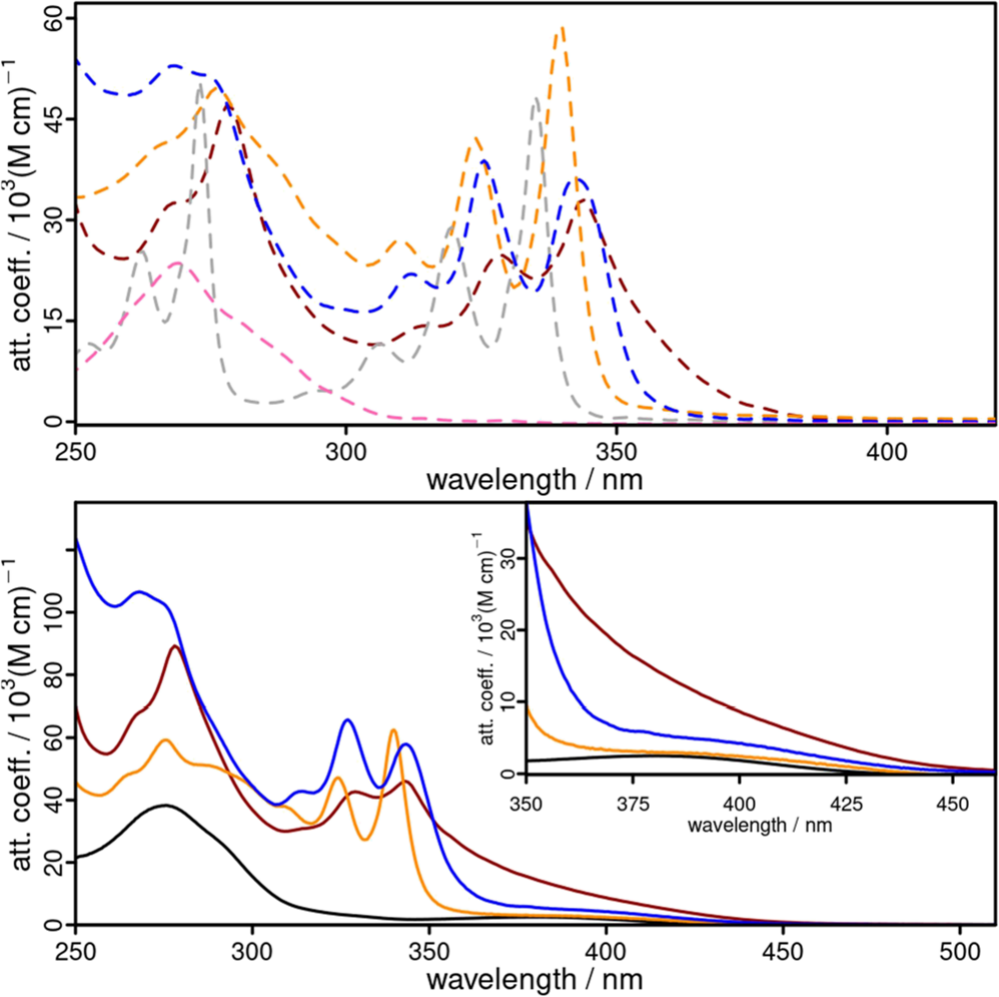
Experimental UV/vis absorption spectra in acetonitrile solution. Top: **Pyr1** (dark red, dashed), **Pyr2** (orange, dashed), **Pyr3** (blue, dashed), **Neo** (pink, dashed), and pyrene (gray, dashed); bottom: **CuPyr1** (dark red), **CuPyr2** (orange), **CuPyr3** (blue), and **CuNeo** (black).

The UV/vis absorption spectra of **Pyr1**–**3** (Figure 3, top) show strong features between 250 and 370 nm in acetonitrile solution, with **Pyr1** having the most red-shifted absorption profile. Based on the spectra of pure pyrene and unsubstituted phenanthroline, the bands of **Pyr1**–**3** between 250 and 300 nm can be assigned to a mixture of (i) ligand-centered (LC) π–π* transitions at the phenanthroline core as well as (ii) LC transition from S_0_ to S_3_ at the pyrenyl substituent.^101^ In the range from 300 to 360 nm, the spectra of the pyrenyl substituted ligands are dominated by intense and clearly structured vibrational bands of the S_0_ to S_2_ LC transition at the pyrenyl moiety.^103,104^ The redshift of **Pyr2** compared to pure pyrene is 4 nm, while 9 nm is observed for **Pyr1** and **Pyr3**. Overall, the impact of pyrene substituents on the absorption of the phenanthroline moiety is not significant, in contrast to 5,5′-bis(pyren-1-yl)-2,2′-bipyridine, where a strong and broad absorption band between 320 and 420 nm was observed.^79^

The absorption features of the bichromophoric **CuPyr1–3** complexes possess similar features compared to those of their corresponding pyrenyl-substituted ligands **Pyr1**–**3** (Figure S42). Especially between **CuPyr2** and **Pyr2**, the difference in the attenuation coefficients is rather small (*i.e.*, Δ_340 nm_ = 4 × 10^3^ Lmol^–1^ cm^–1^, cf. Figure S42 left). In strong contrast, **CuPyr1** and **CuPyr3** show more pronounced absorptivity compared to their respective ligands (Δ_343 nm_ = 13 × 10^3^ Lmol^–1^ cm^–1^ for **CuPyr1** vs **Pyr1** and Δ_327 nm_ = 31 × 10^3^ Lmol^–1^ cm^–1^ for **CuPyr3** vs **Pyr3**; Figure S42 right).

As known from the literature, the absorption of **CuNeo** features a weak broad band between 360 and 450 nm attributed to singlet metal-to-ligand charge transfer (^1^MLCT) transitions from d(Cu) to π*(phen).^35,39^ In this spectral region, the absorption profile of **CuPyr2** is very similar to that of **CuNeo**, with almost identical attenuation coefficients. Together with the fact that **CuPyr2** has the lowest redshift compared to **CuPyr1** and **CuPyr3**, it is suggested that there is only a small orbital overlap between the pyrenyl substituent and the phenanthroline core. It can be concluded that **CuPyr2** has the lowest electronic communication among the complexes **CuPyr1–3**.

In contrast to the pyren-2-yl substitution in 4- and 7-positions (as in **CuPyr2**), such a substitution in 5- and 6-positions in **CuPyr3** almost doubles the attenuation coefficient of the ^1^MLCT band and simultaneously shifts the S_2_ ← S_0_ transition (Figure 3). A stronger electronic interaction between phenanthroline and pyrene is therefore likely in **CuPyr3** compared to **CuPyr2**.

The absorption of **CuPyr1** in the ^1^MLCT region (*i.e.*, around 350–450 nm) exhibits a greater ability to absorb visible light compared to pyren-2-yl substituted systems, as already seen for the **Pyr1** ligand, where the broadest low energy band stretches to longer wavelengths. DFT calculations also indicate that the LUMO+1, which is a dominant part of the ^1^MLCT transition of **CuPyr1**, consists of a mixture of π*_phen_ and π*_pyr_ localized orbitals (Table S1 transition 2). This is quite different in **CuPyr2** and **CuPyr3**, where significant transitions into the π*_pyr_ occur only at higher energies (382 nm for **CuPyr1** vs 324 and 344 nm for **CuPyr2** and **CuPyr3**, respectively; see Tables S1–S3). The predicted trend that pyrene-based transitions requiring the highest energy are present in **CuPyr2** does correlate well with the experimental data (Figures 3, S41, and Tables S1–S3). Taken together, the observed improvement in the light-harvesting properties of **CuPyr1** is attributed to the supposed strong electronic interaction between the two moieties, which is in agreement with the literature.^89^

**Figure 4:**
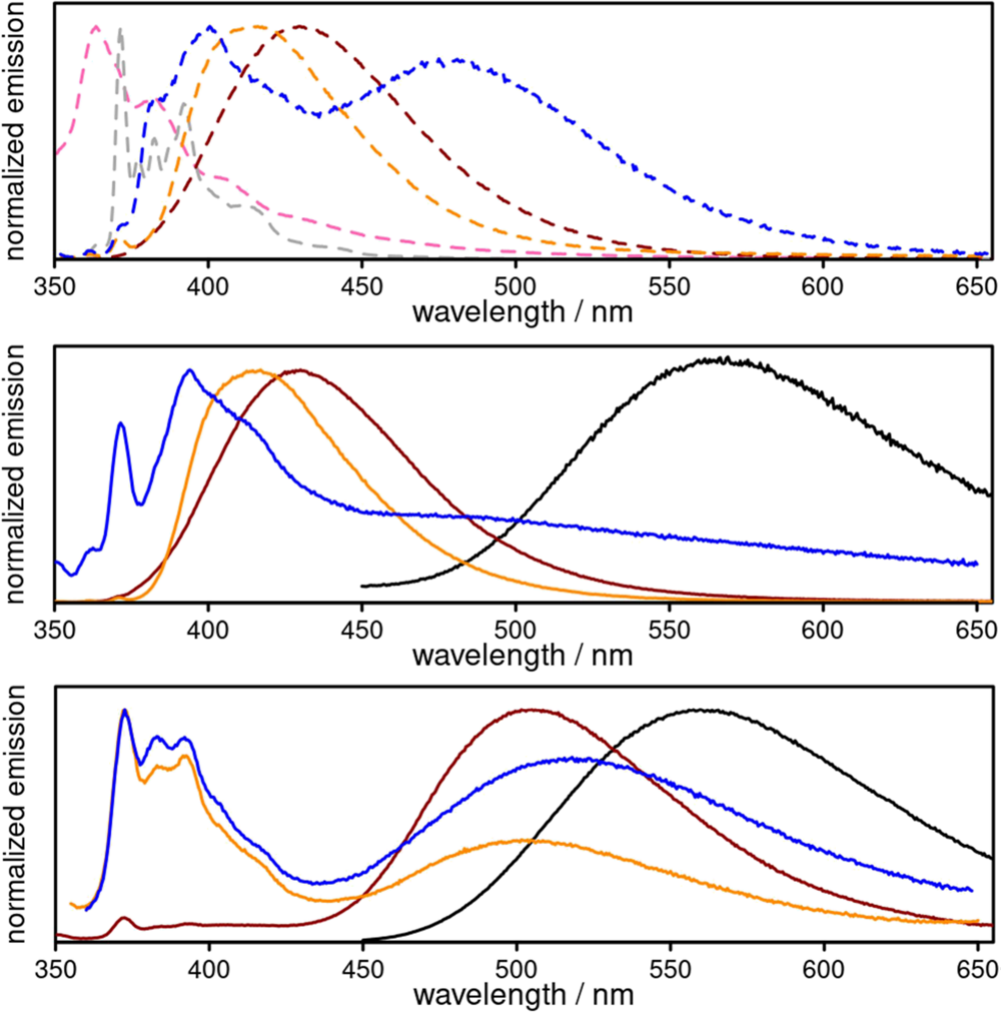
Emission spectra of the ligands (top, dashed, in acetonitrile) and copper(I) complexes (solid) in acetonitrile (middle) and dichloromethane (bottom), respectively, excited at 334 nm under inert conditions. Top: **Neo** (pink), pyrene (gray), **Pyr1** (dark red), **Pyr2** (orange), and **Pyr3** (blue). Middle and bottom: pyrene (gray), **CuNeo** (black), **CuPyr1** (red), **CuPyr2** (orange), and **CuPyr3** (blue).

Emission measurements were then conducted to gain a better understanding of the excited states. Pyrene and **Neo** exhibit structured emissions in the range of 350 to 450 nm (Figure 4 top),^104,107^ whereas the novel ligands **Pyr1–3** show a much broader emission spectrum, extending from 370 to 600 nm. **Pyr1** and **Pyr2** have broad, barley-structured emission bands in acetonitrile, with maxima that are red-shifted relative to the emission of pyrene (59 and 45 nm, respectively) and **Neo** (67 and 53 nm, respectively; Figure 4 top and Table S4). **Pyr3** exhibits a broad emission, consisting of two bands at 400 and 480 nm.

**Figure 5:**
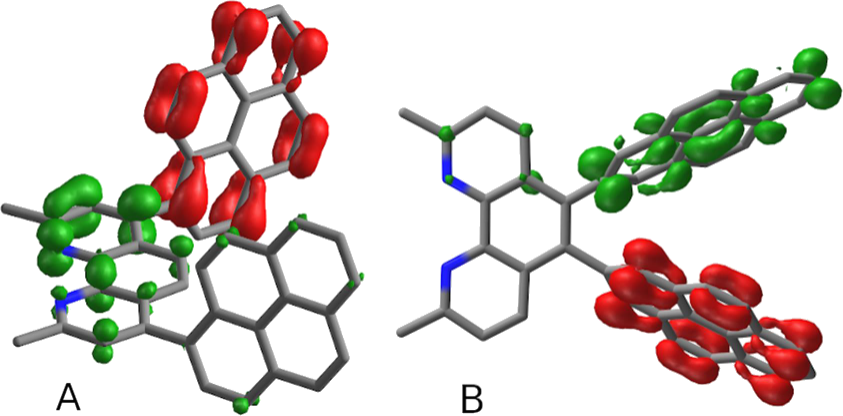
Selected electron difference density plots visualizing the predicted singlet transitions ^1^ILCT_pyr,phen_ (A, **Pyr1**, ca. 350 nm) and ^1^ILCT_pyr,pyr_ (B, **Pyr3**, ca. 280 nm). Decreasing and increasing electron density is shown in red and green (for details, see the SI).

The emission profiles of **Pyr1–3** show considerable differences when compared in dichloromethane and acetonitrile because two bands appear in dichloromethane (Figure S45). This emission behavior has been confirmed by TD-DFT calculations, which show that intraligand charge transfer transitions from π_pyr_ to π*_phen_ (^1^ILCT_pyr,phen_) and transitions from one pyrenyl substituent to the other (^1^ILCT_pyr,pyr_, Figure 5) are possible. Furthermore, the intensity of the bands depends on the excitation wavelength (SI for further details) in the experiment.

To prove the involvement of the phenanthroline moiety in the assumed^1^ILCT_pyr,phen_ transition, protonation experiments with trifluoroacetic acid (TFA, 100 equiv.) were conducted on **Pyr1**–**3** (Figures S46, 47 and Table S4). Upon protonation in acetonitrile, the emission of **Pyr2** displayed a characteristic pyrene-based ligand-centered emission at 375 nm and a red-shifted (Δ = 269 nm) emission band originating from the protonated phenanthroline-1-ium to pyrene at 685 nm. The emission band of **Pyr1** exhibits a redshift of 217 nm, while the second emission band of **Pyr3** has a redshift of 121 nm. Similar results with TFA were also reported by Constable et al.^79^ Interestingly, in dichloromethane, the second emission from **Pyr1** and **Pyr3** (Figure S46) does not change significantly as a result of protonation, indicating the^1^ILCT_pyr,pyr_ emission. A similar interaction between two pyrenyl substituents is referred to in the literature as excimer emission.^74,107^

The complexes **CuPyr1** and **CuPyr2** show identical emission bands in acetonitrile to their corresponding ligands (Figure 4 middle), when excited at 334 nm. These bands are invariant to the excitation wavelength (in the range between 334–355 nm, Figure S48) and can be assigned to a decay of an ILCT_phen,pyr_ state. The emission quantum yield of **CuPyr1** is 32%, much higher than that of **CuPyr2** (9%), which again indicates a significantly stronger electronic interaction between the two chromophores of **CuPyr1**. In contrast to **CuPyr1** and **CuPyr2** in acetonitrile, **CuPyr3** displays a pyrene-structured emission centered at 400 nm and a very broad but weak emission attributed to an ILCT_phen,pyr_. This latter emission is analogous to **Pyr3**.

**CuPyr1** is the only emissive compound in acetonitrile when excited at 407 nm, with an emission peak at 539 nm, attributed to ^3^MLCT from d(Cu) to π*(phen). In contrast, the emission upon excitation at 387 nm appears to originate from a mixture of ILCT_pyr,pyr_ and MLCT_Cu,phen_ (Figure S49). This assignment is supported by the absorption analysis and TD-DFT calculations.

The emission of **CuPyr1** in dichloromethane, excited at 334 nm, is very weak and red-shifted compared to its emission in acetonitrile. **CuPyr2** and **CuPyr3** do not really emit and the two emission bands, one based on LC_pyr_ (at about 375 nm) and the other centered at 505 and 515 nm, respectively (Figure 4 bottom), are only in the order of Raman scattering.

The excited-state characteristics of **Pyr1–3** and **CuPyr1–3** were further studied by time-resolved spectroscopy excited at 355 nm in deaerated acetonitrile and dichloromethane solutions (Figures S50–S53). The emission lifetimes of **Pyr1** and protonated **Pyr1H** were below 10 ns, while **Pyr2** and **Pyr3** also possess rather short-lived emissive states (28.6 and 29.7 ns, respectively). However, transient absorption spectroscopy revealed the presence of dark, long-lived excited states of **Pyr1–3** in the range of 3.71–26.43 μs (Table S5). Based on similarities with other reports of pyrene-based systems,^71,72^ in which the pyrene-localized triplet states also act as the lowest-lying excited states with extended excited state lifetimes, the observed lifetimes of **Pyr1** can be assigned to ^3^LC_pyr_ (16.03 and 26.43 μs in acetonitrile and dichloromethane, respectively). Compared to the triplet lifetime of pyrene (1.23 μs in dichloromethane),^105^ the excited state lifetime of **Pyr1** in the same solvent is increased 22-fold due to the attached phenanthroline moiety.

In line with the results obtained for **Pyr1**, **CuPyr1** also has a short-lived emission (<10 ns) and a long-lived dark excited state (22.42 and 35.95 μs in acetonitrile and dichloromethane, respectively). Therefore, the lowest excited state in **CuPyr1** can be assigned to a non-emissive pyrene-localized ^3^LC state. However, similar results were obtained for **Pyr2** and the respective copper(I) complex (Table S5). Although the excited state lifetimes of **CuPyr2** are somewhat shorter (*e.g.*, 17.70 (acetonitrile) and 25.02 μs (dichloromethane)), the similar magnitude also suggests a similar pyrene-localized ^3^LC state as the final excited state.

For **Pyr1** and **Pyr2** and their respective complexes **CuPyr1** and **CuPyr2**, the excited state lifetimes generally show an increase by a factor of about 1.5–2.5 when the solvent is changed from acetonitrile to dichloromethane. This is consistent with the expected behavior when a coordinating solvent is replaced by a non-coordinating solvent.^108,109^ This solvent effect in our pyrene-based systems differs from other results regarding the solvent-tuning effect of MLCT triplet energies.^47,110^ In these studies, both MLCT emission and solvatochromic behavior of the MLCT states were observed.^47,110^ Even a switch between both the excited state could be achieved when changing the solvent environment.^110^ In contrast, the small impact on the emission wavelength in **CuNeo** (only 4 nm, Table 2) and the slight increase in the excited state lifetime for **CuPry1–3** in dichloromethane renders a change in the nature of the final excited state unlikely.

Interestingly, **CuPyr3** differs significantly from **CuPyr1** and **CuPyr2**, as a second, slightly shorter decay component with a lifetime of ∼1 μs is observed in both solvents. Within this time, an excited state is populated and subsequently depopulated. Since the lifetime of pure triplet pyrene in dichloromethane is 1.23 μs,^105^ it is possible that a triplet state of pyrene forms initially and interacts with the neighboring pyrene, which is still in the ground state. As the distance between the two pyrenes in **CuPyr3** (see Figure 1) is much shorter than in **CuPyr1** and **CuPyr2**, such an interaction could be present. This could be a reason for the much shorter excited state lifetime of **CuPyr3** compared to **CuPyr1** and **CuPyr2**.

### Singlet Oxygen Measurements

The time-resolved measurements indicate that the lowest triplet excited state changes from a ^3^MLCT state in **CuNeo** to a pyrene-localized ^3^LC state in **CuPyr1–3**, associated with an increase in the excited state lifetime of more than one order of magnitude compared to **CuNeo** (*vide supra*). Therefore, the next step was to test whether these promising photophysical properties can be used to generate reactive singlet oxygen (^1^O_2_) and to determine the individual catalytic activities.

^1^O_2_ is the energetically higher form of triplet oxygen (^3^O_2_) and, due to its singlet electronic configuration, commonly used as a reagent for oxidation reactions. Furthermore, ^1^O_2_ is also used in photodynamic therapy (PDT) in the treatment of cancer, for example. Mechanistically, ^1^O_2_ is generated through energy transfer from ^3^O_2_. The efficiency of this process can generally be evaluated from the characteristic emission band of ^1^O_2_ at about 1276 nm. As a universal standard to quantify this emission, phenalenone (PN) can be used.^111,112^

In aerated acetonitrile, all four copper(I) photosensitizers display a clear ^1^O_2_ emission band when excited at 407 nm (Figure S54). As expected, **CuPyr1**, which has the longest excited state lifetime, provides the highest ^1^O_2_ quantum yield of 0.96 with respect to PN (ϕ_1O2_ = 1).^111−114^ Thus, **CuPyr1** outperforms **CuPyr2** (0.83) and **CuPyr3** (0.66) and is also clearly superior to the reference complex **CuNeo** (0.20). This trend clearly correlates with the excited state lifetimes obtained from the TA measurements.

**Figure 6:**
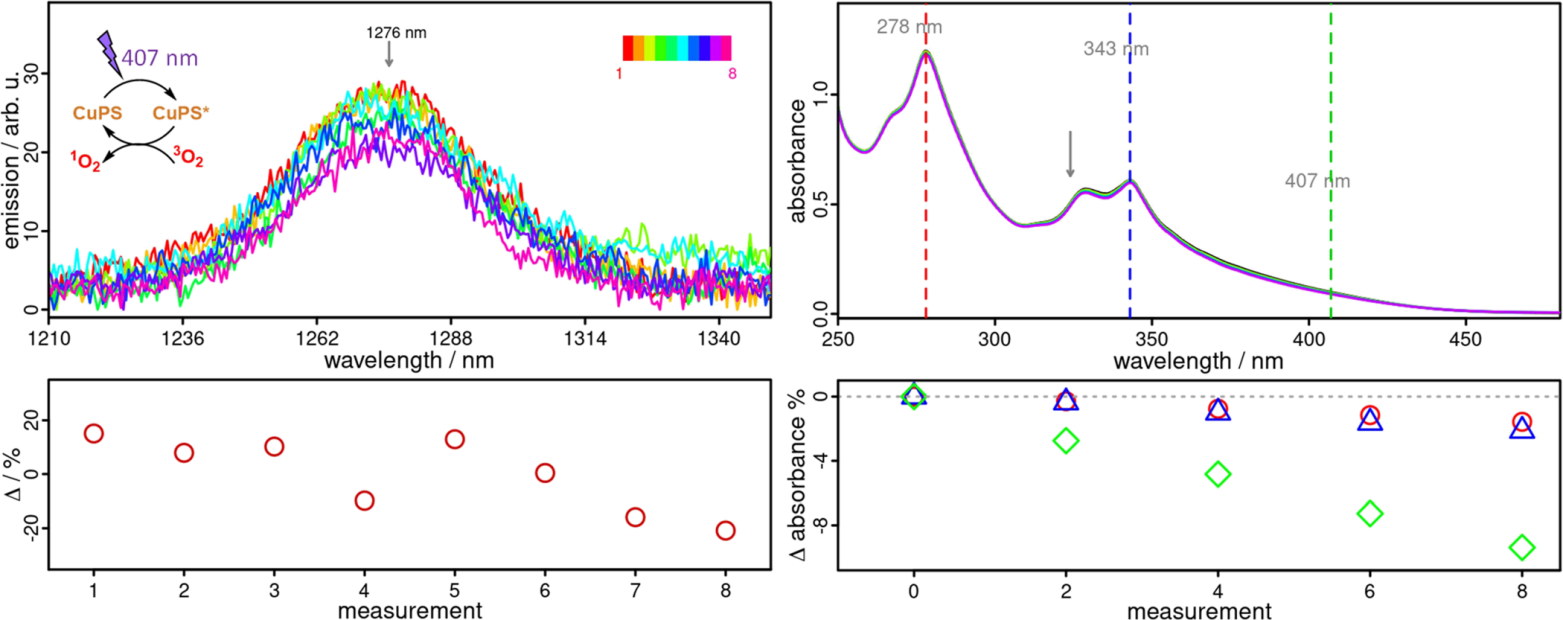
Left Top: Near-infrared emission spectra of **CuPyr1** in aerated acetonitrile solution after excitation at 407 nm showing the characteristic ^1^O_2_ emission at 1276 nm. Left bottom: Relative differences of the recorded ^**1**^O_2_ compared to the averaged value of eight subsequent measurements (see the SI for further explanation). Right top: UV/vis absorption spectra of the same sample after the emission measurements for testing the stability. Right bottom: Relative differences in the absorbance of **CuPyr1** monitored at 278 (red), 343 (blue), and 407 nm (green).

To evaluate the ability of the photosensitizers to produce ^1^O_2_ continuously and to be reusable without significant loss of activity, successive ^1^O_2_ emission spectra of the same sample were recorded in conjunction with subsequent photostability measurements. In addition, the photostability of **CuPyr1–3** was investigated over a period of 3 h in inert acetonitrile and at least 1 h under aerated conditions (Figures S57 and S58). Figure 6 successfully demonstrates the constant activity in ^1^O_2_ generation of **CuPyr1** over the course of 8 measurements, with only a slight decrease of the MLCT band (Δ = 9%). This small depletion is possibly due to photobleaching by in situ generated ^1^O_2_ or photo-induced ligand exchange processes, as known from the literature.^115−117^ Similarly, for **CuPyr2** and **CuPyr3**, the continuity of ^1^O_2_ generation and the subsequent photostability are not affected by the substitution characteristics (compare Figures 6 and S55, S56). Nevertheless, all three bichromophoric complexes exhibit increased photoactivity, highlighting the success of this design strategy.

### Photocatalytic Oxidation of 1,5-Dihydroxy-Naphthalene

**Figure 7:**
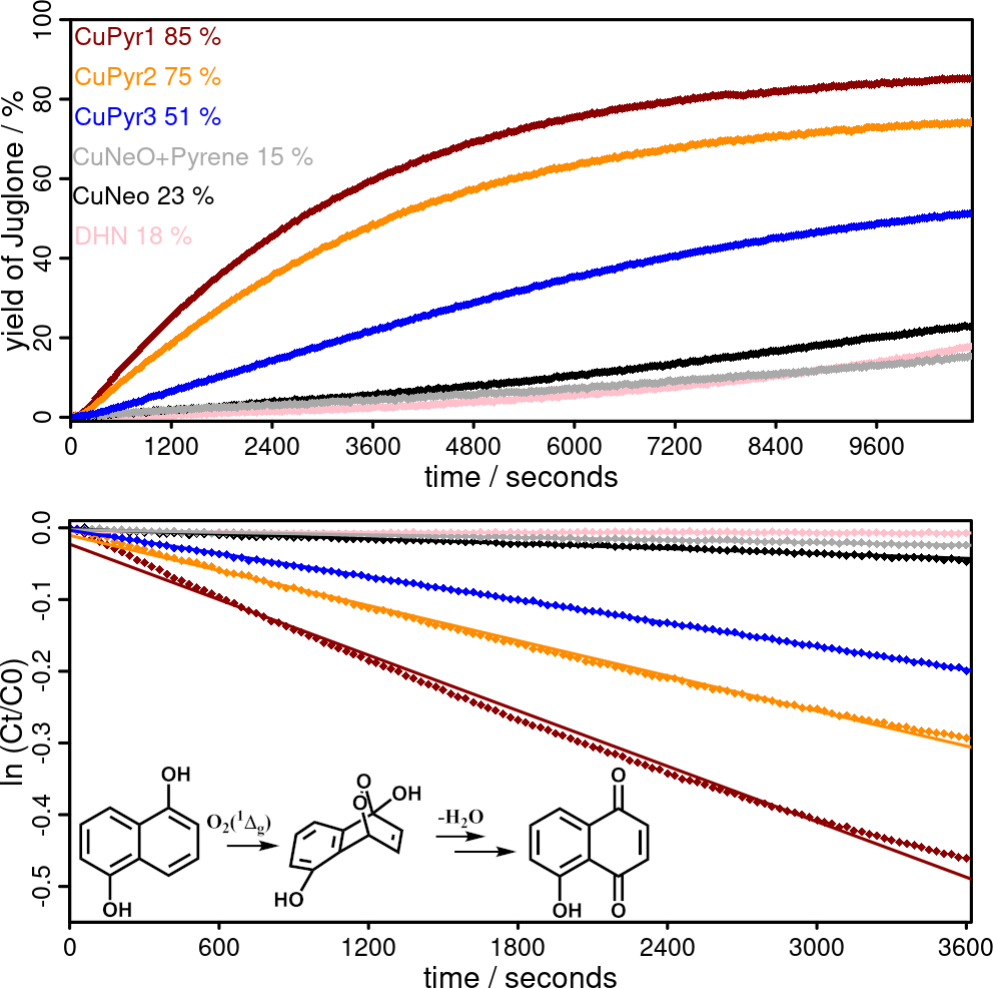
Top: Time traces of the formation of juglone (yield juglone vs reaction time) in the photooxidation reaction of DHN sensitized by the three novel complexes **CuPyr1–3** (red, orange, blue), a 1:2 mixture of **CuNeo** + pyrene (gray) and **CuNeo** (black). The pink trace shows the blank measurement without a photosensitizer. Bottom: Logarithmic plot of decreasing concentration of DHN over time. Dots represent the actual experimental data, which are fitted to a linear relationship assuming the first-order reaction kinetic (solid lines). The inset shows the reaction equation of the photooxidation of 1,5-DHN to juglone by ^1^O_2_.

The pyrene-substituted copper(I) complexes exhibit a promising activity to generate ^1^O_2_ and an excellent photostability, making them attractive for other visible-light photocatalytic applications. 5-Hydroxy-1,4-naphthoquinone, commonly known as juglone, has been demonstrated to have interesting anticancer properties.^118^ Juglone can be derived from 1,5-dihydroxynaphthalene (DHN) by an oxidation reaction utilizing ^1^O_2_ (Figure 7 bottom).^78,96^ The oxidative conversion of DHN to juglone represents a significant increase in value (∼$1/g vs ∼$300/g). Therefore, we aimed to investigate the novel photosensitizers **CuPyr1–3** for the photooxidation of DHN in acetonitrile solution. Main attention was given to the kinetics of the photocatalytic reaction and the estimation of the final yields of juglone. The progress of the photooxidation of DHN was monitored by in situ UV/vis spectroscopy by measuring the changes in the absorption (Figures S59–S62) of DHN at 301 nm and the formation of juglone at 427 nm, as described in previous studies.^94−96^ The measurements (Figure 7) indicate that the catalytic process proceeds via a pseudo first-order reaction, which is in agreement with earlier studies.^78,95,96^ All pyrene-based complexes display larger rate constants *k*_obs_ compared to both the reference system (**CuNeo** alone) and a physical mixture of **CuNeo** and pyrene (Table 3). Among the complexes, **CuPyr1** has a rate constant that is up to ten times larger than **CuNeo**. The yield of juglone obtained with the most efficient copper(I) photosensitizer, **CuPyr1** (85%) is comparable to those achieved with noble metal-based iridium(III) complexes (80–99%).^78^ The observed rate constant for **CuPyr1** (12.9 × 10^–5^ s^–1^) is also in a similar range to those of the precious metal complexes (25–58 × 10^–5^ s^–1^).^78^ Although an exact comparison is difficult due to many parameters affecting the kinetics, the more earth-abundant copper(I) derivatives represent a viable alternative to noble metal-based photosensitizers.

**Table 3:** Results of the Photocatalytic Oxidation of 1,5-Dihydroxynaphthalene (DHN) to Juglone^a^

parameter	*k*_obs_^b^ (10^–5^ s^–1^)	yield (%)
no light	-	<0.01
no PS	<0.1	18
**CuNeo**	1.1	23
**CuNeo** + pyrene	0.6	15
**CuPyr1**	12.9	85
**CuPyr2**	8.2	75
**Cupyr3**	5.4	51

a Conditions: in aerated acetonitrile under Xe lamp irradiation with a 380 nm long pass filter and in the dark (for more detailed information, see the SI).

b Rate constant determined from ln(*C_t_*/*C*_0_) assuming a first-order kinetics.

## Conclusions

A trio of different pyrene-substituted phenanthroline-based ligands **Pyr1–3** and their respective heteroleptic copper(I) photosensitizers **CuPyr1–3** were analyzed concerning two strategies: (i) substitution at different positions on pyrene (*i.e.*, 1-yl vs 2-yl) and (ii) alternation of the position on phenanthroline (*i.e.*, 4,7 vs 5,6). TD-DFT calculations revealed that there is a remarkable difference between the singlet and triplet state of the torsion angles around the newly formed C–C bond Δτ_Sub_(GS-ES), where the substituents in **CuPyr1** are more twisted compared to **CuPyr2** and **CuPyr3**. This already indicates the presence of significant electronic coupling, especially in **CuPyr1**. This was also demonstrated by electrochemistry, as **CuPyr1** showed the most anodically shifted reduction potential among the complexes. In general, the phenanthroline-based reduction potential is determined by the substitution position at the phenanthroline (*i.e.*, 4,7 vs 5,6), irrespective of the substitution pattern at pyrene (1-yl vs 2-yl). The reduction potential of the pyrene moiety, however, is strongly dictated by the substitution pattern at pyrene. Importantly, all novel complexes gained the ability to reversibly store a total of at least three electrons, while the reference complex **CuNeo** can only be reduced once.

The general observation that for **Pyr1** and **CuPyr1** the electronic interaction between the two building blocks is most pronounced (*i.e.*, between phenanthroline and pyrene) is reflected in all the spectroscopic techniques applied (UV/vis, emission, time-resolved luminescence, and transient absorption). For instance, the absorption is more intense and most redshifted, the emission intensities are highest, and the final excited ^3^LC state is the most long-lived (*e.g.*, **Pyr1** 16.03 μs and **CuPyr1** 22.42 μs). Compared to **CuNeo** (0.20 μs), the excited state lifetime is increased by more than two orders of magnitude. This directly correlates with the highest singlet oxygen quantum yield (96%) and the most beneficial catalytic activity in the photocatalytic oxidation of 1,5-dihydroxy-naphthalene to the anti-cancer drug juglone. In this reaction, the observed rate constant is twelve times greater, and the yield four times higher compared to the unsubstituted reference complex **CuNeo**.

In conclusion, all three bichromophoric copper(I) complexes are highly efficient photosensitizers that can rival with noble metal-based competitors. Especially, the fact that a total of three electrons can be reversibly stored on the ligand scaffold in combination with the high driving force of the underlying reduction potentials (*e.g.*, −2.67 V vs Fc/Fc^+^) calls for more demanding catalytic reactions like the dehalogenation of aryl chlorides in the future.

## Supporting Information Available

Experimental and synthetic details, NMR, MS, UV/vis, and emission spectra; electrochemical data; results from DFT calculations and excited state lifetime measurements; and details concerning catalytic experiments (PDF)

## References

[ref1] Nalley S.; LaRose A. Annual Energy Outlook 2021, 2021.

[ref2] Armaroli N.; Balzani V. Solar Electricity and Solar Fuels: Status and Perspectives in the Context of the Energy Transition. Chem. – Eur. J. 2016, 22, 32–57. 10.1002/chem.201503580. 26584653

[ref3] Detz R. J.; Reek J. N. H.; van der Zwaan B. C. C. The future of solar fuels: when could they become competitive?. Energy Environ. Sci. 2018, 11, 1653–1669. 10.1039/C8EE00111A.

[ref4] Gür T. M. Review of electrical energy storage technologies, materials and systems: challenges and prospects for large-scale grid storage. Energy Environ. Sci. 2018, 11, 2696–2767. 10.1039/C8EE01419A.

[ref5] Hammarström L. Catalyst: Chemistry’s Role in Providing Clean and Affordable Energy for All. Chem 2016, 1, 515–518. 10.1016/j.chempr.2016.09.002.

[ref6] Eckenhoff W. T.; Eisenberg R. Molecular systems for light driven hydrogen production. Dalton Trans. 2012, 13004–13021. 10.1039/c2dt30823a. 23014879

[ref7] Luo S.-P.; Mejía E.; Friedrich A.; Pazidis A.; Junge H.; Surkus A.-E.; Jackstell R.; Denurra S.; Gladiali S.; Lochbrunner S.; Beller M. Photocatalytic Water Reduction with Copper-Based Photosensitizers: A Noble-Metal-Free System. Angew. Chem., Int. Ed. 2013, 52, 419–423. 10.1002/anie.201205915. 23047871

[ref8] Khnayzer R. S.; McCusker C. E.; Olaiya B. S.; Castellano F. N. Robust Cuprous Phenanthroline Sensitizer for Solar Hydrogen Photocatalysis. J. Am. Chem. Soc. 2013, 135, 14068–14070. 10.1021/ja407816f. 24028290

[ref9] Pfeffer M. G.; Kowacs T.; Wächtler M.; Guthmuller J.; Dietzek B.; Vos J. G.; Rau S. Optimization of Hydrogen-Evolving Photochemical Molecular Devices. Angew. Chem., Int. Ed. 2015, 54, 6627–6631. 10.1002/anie.201409442. 25858688

[ref10] Kim J.; Whang D. R.; Park S. Y. Designing Highly Efficient Cu^I^ Photosensitizers for Photocatalytic H_2_ Evolution from Water. ChemSusChem 2017, 10, 1883–1886. 10.1002/cssc.201700389. 28332772

[ref11] Yuan Y.-J.; Yu Z.-T.; Chen D.-Q.; Zou Z.-G. Metal-complex chromophores for solar hydrogen generation. Chem. Soc. Rev. 2017, 46, 603–631. 10.1039/C6CS00436A. 27808300

[ref12] Berardi S.; Drouet S.; Francàs L.; Gimbert-Suriñach C.; Guttentag M.; Richmond C.; Stoll T.; Llobet A. Molecular artificial photosynthesis. Chem. Soc. Rev. 2014, 43, 7501–7519. 10.1039/C3CS60405E. 24473472

[ref13] Nakajima T.; Tamaki Y.; Ueno K.; Kato E.; Nishikawa T.; Ohkubo K.; Yamazaki Y.; Morimoto T.; Ishitani O. Photocatalytic Reduction of Low Concentration of CO_2_. J. Am. Chem. Soc. 2016, 138, 13818–13821. 10.1021/jacs.6b08824. 27704819

[ref14] Dalle K.; Warnan J.; Leung J. J.; Reuillard B.; Karmel I. S.; Reisner E. Electro- and Solar-Driven Fuel Synthesis with First Row Transition Metal Complexes. Chem. Rev. 2019, 119, 2752–2875. 10.1021/acs.chemrev.8b00392. 30767519 PMC6396143

[ref15] Steinlechner C.; Roesel A. F.; Oberem E.; Päpcke A.; Rockstroh N.; Gloaguen F.; Lochbrunner S.; Ludwig R.; Spannenberg A.; Junge H.; Francke R.; Beller M. Selective Earth-Abundant System for CO_2_ Reduction: Comparing Photo- and Electrocatalytic Processes. ACS Catal. 2019, 9, 2091–2100. 10.1021/acscatal.8b03548.

[ref16] Call A.; Cibian M.; Yamauchi K.; Sakai K. Visible-light-driven reduction of CO_2_ to CO in fully aqueous media using a water-soluble cobalt porphyrin. Sustainable Energy Fuels 2022, 6, 2160–2164. 10.1039/D2SE00291D.

[ref17] Giereth R.; Lang P.; McQueen E.; Meißsner X.; Braun-Cula B.; Marchfelder C.; Obermeier M.; Schwalbe M.; Tschierlei S. Elucidation of Cooperativity in CO_2_ Reduction Using a Xanthene-Bridged Bimetallic Rhenium(I) Complex. ACS Catal. 2021, 11, 390–403. 10.1021/acscatal.0c04314.

[ref18] Otto S.; Nauth A. M.; Ermilov E.; Scholz N.; Friedrich A.; Resch-Genger U.; Lochbrunner S.; Opatz T.; Heinze K. Photo-Chromium: Sensitizer for Visible-Light-Induced Oxidative C–H Bond Functionalization—Electron or Energy Transfer?. ChemPhotoChem 2017, 1, 344–349. 10.1002/cptc.201700077.

[ref19] Schmid M.-A.; Brückmann J.; Bösking J.; Nauroozi D.; Karnahl M.; Rau S.; Tschierlei S. Merging of a Perylene Moiety Enables a RuII Photosensitizer with Long-Lived Excited States and the Efficient Production of Singlet Oxygen. Chem. – Eur. J. 2022, 28, e202103609 10.1002/chem.202103609. 34767288 PMC9299699

[ref20] Rentschler M.; Boden P. J.; Argüello Cordero M. A.; Steiger S. T.; Schmid M.-A.; Yang Y.; Niedner-Schatteburg G.; Karnahl M.; Lochbrunner S.; Tschierlei S. Unexpected Boost in Activity of a Cu(I) Photosensitizer by Stabilizing a Transient Excited State. Inorg. Chem. 2022, 61, 12249–12261. 10.1021/acs.inorgchem.2c01468. 35877171

[ref21] Day J. I.; Singh K.; Trinh W.; Weaver J. D. I. Visible Light Mediated Generation of trans-Arylcyclohexenes and Their Utilization in the Synthesis of Cyclic Bridged Ethers. J. Am. Chem. Soc. 2018, 140, 9934–9941. 10.1021/jacs.8b04642. 30001489

[ref22] Cruché C.; Neiderer W.; Collins S. K. Heteroleptic Copper-Based Complexes for Energy-Transfer Processes: E → Z Isomerization and Tandem Photocatalytic Sequences. ACS Catal. 2021, 11, 8829–8836. 10.1021/acscatal.1c01983.

[ref23] Neveselý T.; Wienhold M.; Molloy J. J.; Gilmour R. Advances in the E → Z Isomerization of Alkenes Using Small Molecule Photocatalysts. Chem. Rev. 2022, 122, 2650–2694. 10.1021/acs.chemrev.1c00324. 34449198

[ref24] Ratani T. S.; Bachman S.; Fu G. C.; Peters J. C. Photoinduced, Copper-Catalyzed Carbon-Carbon Bond Formation with Alkyl Electrophiles: Cyanation of Unactivated Secondary Alkyl Chlorides at Room Temperature. J. Am. Chem. Soc. 2015, 137, 13902–13907. 10.1021/jacs.5b08452. 26491957 PMC4666296

[ref25] Patel R. I.; Sharma S.; Sharma A. Cyanation: a photochemical approach and applications in organic synthesis. Org. Chem. Front. 2021, 8, 3166–3200. 10.1039/D1QO00162K.

[ref26] Choi G. J.; Zhu Q.; Miller D. C.; Gu C. J.; Knowles R. R. Catalytic alkylation of remote C-H bonds enabled by proton-coupled electron transfer. Nature 2016, 539, 268–271. 10.1038/nature19811. 27732585 PMC5704892

[ref27] Treacy S. M.; Rovis T. Copper Catalyzed C(sp3)-H Bond Alkylation via Photoinduced Ligand-to-Metal Charge Transfer. J. Am. Chem. Soc. 2021, 143, 2729–2735. 10.1021/jacs.1c00687. 33576606 PMC8608032

[ref28] Cuthbertson J. D.; MacMillan D. W. C. The direct arylation of allylic sp3 C-H bonds via organic and photoredox catalysis. Nature 2015, 519, 74–77. 10.1038/nature14255. 25739630 PMC4378681

[ref29] Das S.; Murugesan K.; Villegas Rodríguez G. J.; Kaur J.; Barham J. P.; Savateev A.; Antonietti M.; König B. Photocatalytic (Het)arylation of C(sp3)-H Bonds with Carbon Nitride. ACS Catal. 2021, 11, 1593–1603. 10.1021/acscatal.0c05694.

[ref30] Prier C. K.; Rankic D. A.; MacMillan D. W. C. Visible Light Photoredox Catalysis with Transition Metal Complexes: Applications in Organic Synthesis. Chem. Rev. 2013, 113, 5322–5363. 10.1021/cr300503r. 23509883 PMC4028850

[ref31] Schultz D. M.; Yoon T. P. Solar Synthesis: Prospects in Visible Light Photocatalysis. Science 2014, 343, 1239176 10.1126/science.1239176. 24578578 PMC4547527

[ref32] Hockin B. M.; Li C.; Robertson N.; Zysman-Colman E. Photoredox catalysts based on earth-abundant metal complexes. Catal. Sci. Technol. 2019, 9, 889–915. 10.1039/C8CY02336K.

[ref33] Beaudelot J.; Oger S.; Peruško S.; Phan T.-A.; Teunens T.; Moucheron C.; Evano G. Photoactive Copper Complexes: Properties and Applications. Chem. Rev. 2022, 122, 16365–16609. 10.1021/acs.chemrev.2c00033. 36350324

[ref34] Mejía E.; Luo S.-P.; Karnahl M.; Friedrich A.; Tschierlei S.; Surkus A.-E.; Junge H.; Gladiali S.; Lochbrunner S.; Beller M. A Noble-Metal-Free System for Photocatalytic Hydrogen Production from Water. Chem. – Eur. J. 2013, 19, 15972–15978. 10.1002/chem.201302091. 24123302

[ref35] Zhang Y.; Schulz M.; Wächtler M.; Karnahl M.; Dietzek B. Heteroleptic diimine-diphosphine Cu(I) complexes as an alternative towards noble-metal based photosensitizers: Design strategies, photophysical properties and perspective applications. Coord. Chem. Rev. 2018, 356, 127–146. 10.1016/j.ccr.2017.10.016.

[ref36] Doettinger F.; Yang Y.; Schmid M.-A.; Frey W.; Karnahl M.; Tschierlei S. Cross-Coupled Phenyl- and Alkynyl-Based Phenanthrolines and Their Effect on the Photophysical and Electrochemical Properties of Heteroleptic Cu(I) Photosensitizers. Inorg. Chem. 2021, 60, 5391–5401. 10.1021/acs.inorgchem.1c00416. 33764043

[ref37] Foerster C.; Heinze K. Photophysics and photochemistry with Earth-abundant metals-fundamentals and concepts. Chem. Soc. Rev. 2020, 49, 1057–1070. 10.1039/C9CS00573K. 32025671

[ref38] Wegeberg C.; Wenger O. S. Luminescent First-Row Transition Metal Complexes. JACS Au 2021, 1, 1860–1876. 10.1021/jacsau.1c00353. 34841405 PMC8611671

[ref39] Heberle M.; Tschierlei S.; Rockstroh N.; Ringenberg M.; Frey W.; Junge H.; Beller M.; Lochbrunner S.; Karnahl M. Heteroleptic Copper Photosensitizers: Why an Extended π-System Does Not Automatically Lead to Enhanced Hydrogen Production. Chem. – Eur. J. 2017, 23, 312–319. 10.1002/chem.201604005. 27768809

[ref40] Zhang Y.; Luo Q.; Zheng W.; Wang Z.; Lin Y.; Zhang E.; Lü S.; Xiang J.; Zhao Y.; Wang F. Luminescent cyclometallated platinum (II) complexes: highly promising EGFR/DNA probes and dual-targeting anticancer agents. Inorg. Chem. Front. 2018, 5, 413–424. 10.1039/C7QI00346C.

[ref41] Garbe S.; Krause M.; Klimpel A.; Neundorf I.; Lippmann P.; Ott I.; Brünink D.; Strassert C. A.; Doltsinis N. L.; Klein A. Cyclometalated Pt Complexes of CNC Pincer Ligands: Luminescence and Cytotoxic Evaluation. Organometallics 2020, 39, 746–756. 10.1021/acs.organomet.0c00015.

[ref42] Arias-Rotondo D. M.; McCusker J. K. The photophysics of photoredox catalysis: a roadmap for catalyst design. Chem. Soc. Rev. 2016, 45, 5803–5820. 10.1039/C6CS00526H. 27711624

[ref43] Yarnell J. E.; Deaton J. C.; McCusker C. E.; Castellano F. N. Bidirectional “Ping-Pong” Energy Transfer and 3000-Fold Lifetime Enhancement in a Re(I) Charge Transfer Complex. Inorg. Chem. 2011, 50, 7820–7830. 10.1021/ic200974h. 21761837

[ref44] Wells K. A.; Yarnell J. E.; Sheykhi S.; Palmer J. R.; Yonemoto D. T.; Joyce R.; Garakyaraghi S.; Castellano F. N. Accessing the triplet manifold of naphthalene benzimidazole-phenanthroline in rhenium(i) bichromophores. Dalton Trans. 2021, 50, 13086–13095. 10.1039/D1DT02329B. 34581368

[ref45] Büldt L. A.; Wenger O. S. Chromium complexes for luminescence, solar cells, photoredox catalysis, upconversion, and phototriggered NO release. Chem. Sci. 2017, 8, 7359–7367. 10.1039/C7SC03372A. 29163886 PMC5672834

[ref46] Treiling S.; Wang C.; Förster C.; Reichenauer F.; Kalmbach J.; Boden P.; Harris J. P.; Carrella L. M.; Rentschler E.; Resch-Genger U.; Reber C.; Seitz M.; Gerhards M.; Heinze K. Luminescence and Light-Driven Energy and Electron Transfer from an Exceptionally Long-Lived Excited State of a Non-Innocent Chromium(III) Complex. Angew. Chem., Int. Ed. 2019, 58, 18075–18085. 10.1002/anie.201909325. PMC6916301 31600421

[ref47] Wegeberg C.; Häussinger D.; Wenger O. S. Pyrene-Decoration of a Chromium(0) Tris(diisocyanide) Enhances Excited State Delocalization: A Strategy to Improve the Photoluminescence of 3d6 Metal Complexes. J. Am. Chem. Soc. 2021, 143, 15800–15811. 10.1021/jacs.1c07345. 34516734

[ref48] Reichenauer F.; Wang C.; Förster C.; Boden P.; Ugur N.; Báez-Cruz R.; Kalmbach J.; Carrella L. M.; Rentschler E.; Ramanan C.; Niedner-Schatteburg G.; Gerhards M.; Seitz M.; Resch-Genger U.; Heinze K. Strongly Red-Emissive Molecular Ruby [Cr(bpmp)2]3+ Surpasses [Ru(bpy)3]2+. J. Am. Chem. Soc. 2021, 143, 11843–11855. 10.1021/jacs.1c05971. 34296865

[ref49] Wenger O. S. Is Iron the New Ruthenium?. Chem. – Eur. J. 2019, 25, 6043–6052. 10.1002/chem.201806148. 30615242

[ref50] Kjæer K. S.; Kaul N.; Prakash O.; Chábera P.; Rosemann N. W.; Honarfar A.; Gordivska O.; Fredin L. A.; Bergquist K.-E.; Häggström L.; Ericsson T.; Lindh L.; Yartsev A.; Styring S.; Huang P.; Uhlig J.; Bendix J.; Strand D.; Sundström V.; Persson P.; Lomoth R.; Wärnmark K. Luminescence and reactivity of a charge-transfer excited iron complex with nanosecond lifetime. Science 2019, 363, 249 10.1126/science.aau7160. 30498167

[ref51] Obermeier M.; Beckmann F.; Schaer R. S.; Wenger O. S.; Schwalbe M. Sensitized Photocatalytic CO_2_ Reduction With Earth Abundant 3d Metal Complexes Possessing Dipicolyl-Triazacyclononane Derivatives. Front. Chem. 2021, 9, 751716 10.3389/fchem.2021.751716. 34660540 PMC8514774

[ref52] Leis W.; Argüello Cordero M. A.; Lochbrunner S.; Schubert H.; Berkefeld A. A Photoreactive Iron(II) Complex Luminophore. J. Am. Chem. Soc. 2022, 144, 1169–1173. 10.1021/jacs.1c13083. 35025493

[ref53] Sandroni M.; Pellegrin Y.; Odobel F. Heteroleptic bis-diimine copper(I) complexes for applications in solar energy conversion. C. R. Chim. 2016, 19, 79–93. 10.1016/j.crci.2015.06.008.

[ref54] Leoni E.; Mohanraj J.; Holler M.; Mohankumar M.; Nierengarten I.; Monti F.; Sournia-Saquet A.; Delavaux-Nicot B.; Nierengarten J.; Armaroli N. Heteroleptic Copper(I) Complexes Prepared from Phenanthroline and Bis-Phosphine Ligands: Rationalization of the Photophysical and Electrochemical Properties. Inorg. Chem. 2018, 57, 15537–15549. 10.1021/acs.inorgchem.8b02879. 30481016

[ref55] Forero Cortés P. A.; Marx M.; Trose M.; Beller M. Heteroleptic copper complexes with nitrogen and phosphorus ligands in photocatalysis: Overview and perspectives. Chem. Catal. 2021, 1, 298–338. 10.1016/j.checat.2021.05.005.

[ref56] Gernert M.; Balles-Wolf L.; Kerner F.; Müller U.; Schmiedel A.; Holzapfel M.; Marian C. M.; Pflaum J.; Lambert C.; Steffen A. Cyclic (Amino)(aryl)carbenes Enter the Field of Chromophore Ligands: Expanded π System Leads to Unusually Deep Red Emitting Cu^I^ Compounds. J. Am. Chem. Soc. 2020, 142, 8897–8909. 10.1021/jacs.0c02234. 32302135

[ref57] Schulz M.; Hagmeyer N.; Wehmeyer F.; Lowe G.; Rosenkranz M.; Seidler B.; Popov A.; Streb C.; Vos J. G.; Dietzek B. Photoinduced Charge Accumulation and Prolonged Multielectron Storage for the Separation of Light and Dark Reaction. J. Am. Chem. Soc. 2020, 142, 15722–15728. 10.1021/jacs.0c03779. 32830491

[ref58] Rosko M. C.; Wells K. A.; Hauke C. E.; Castellano F. N. Next Generation Cuprous Phenanthroline MLCT Photosensitizer Featuring Cyclohexyl Substituents. Inorg. Chem. 2021, 60, 8394–8403. 10.1021/acs.inorgchem.1c01242. 34097407

[ref59] Sandoval-Pauker C.; Molina-Aguirre G.; Pinter B. Status report on copper (I) complexes in photoredox catalysis; photophysical and electrochemical properties and future prospects. Polyhedron 2021, 199, 115105 10.1016/j.poly.2021.115105.

[ref60] Armaroli N. Photoactive mono- and polynuclear Cu(I)-phenanthrolines. A viable alternative to Ru(II)-polypyridines?. Chem. Soc. Rev. 2001, 30, 113–124. 10.1039/b000703j.

[ref61] Kuang S.-M.; Cuttell D. G.; McMillin D. R.; Fanwick P. E.; Walton R. A. Synthesis and Structural Characterization of Cu(I) and Ni(II) Complexes that Contain the Bis[2-(diphenylphosphino)phenyl]ether Ligand. Novel Emission Properties for the Cu(I) Species. Inorg. Chem. 2002, 41, 3313–3322. 10.1021/ic0201809. 12055011

[ref62] Lazorski M. S.; Castellano F. N. Advances in the light conversion properties of Cu(I)-based photosensitizers. Polyhedron 2014, 82, 57–70. 10.1016/j.poly.2014.04.060.

[ref63] Paria S.; Reiser O. Copper in Photocatalysis. ChemCatChem 2014, 6, 2477–2483. 10.1002/cctc.201402237.

[ref64] Tsubomura T.; Kimura K.; Nishikawa M.; Tsukuda T. Structures and photophysical properties of copper(i) complexes bearing diphenylphenanthroline and bis(diphenylphosphino)alkane: the effect of phenyl groups on the phenanthroline ligand. Dalton Trans. 2015, 44, 7554–7562. 10.1039/C5DT00835B. 25804312

[ref65] Hernandez-Perez A. C.; Vlassova A.; Collins S. K. Toward a Visible Light Mediated Photocyclization: Cu-Based Sensitizers for the Synthesis of [5]Helicene. Org. Lett. 2012, 14, 2988–2991. 10.1021/ol300983b. 22642645

[ref66] Bao H.; Zhou B.; Luo S.-P.; Xu Z.; Jin H.; Liu Y. P/N Heteroleptic Cu(I)-Photosensitizer-Catalyzed Deoxygenative Radical Alkylation of Aromatic Alkynes with Alkyl Aldehydes Using Dipropylamine as a Traceless Linker Agent. ACS Catal. 2020, 10, 7563–7572. 10.1021/acscatal.0c02454.

[ref67] Hossain A.; Bhattacharyya A.; Reiser O. Copper’s rapid ascent in visible-light photoredox catalysis. Science 2019, 364, eaav9713 10.1126/science.aav9713. 31048464

[ref68] Zhang Y.; Traber P.; Zedler L.; Kupfer S.; Gräfe S.; Schulz M.; Frey W.; Karnahl M.; Dietzek B. Cu(i) vs. Ru(ii) photosensitizers: elucidation of electron transfer processes within a series of structurally related complexes containing an extended π-system. Phys. Chem. Chem. Phys. 2018, 20, 24843–24857. 10.1039/C8CP04595J. 30230487

[ref69] Giereth R.; Reim I.; Frey W.; Junge H.; Tschierlei S.; Karnahl M. Remarkably long-lived excited states of copper photosensitizers containing an extended π-system based on an anthracene moiety. Sustainable Energy Fuels 2019, 3, 692–700. 10.1039/C8SE00521D.

[ref70] Argüello Cordero M. A.; Boden P. J.; Rentschler M.; Di Martino-Fumo P.; Frey W.; Yang Y.; Gerhards M.; Karnahl M.; Lochbrunner S.; Tschierlei S. Comprehensive Picture of the Excited State Dynamics of Cu(I)- and Ru(II)-Based Photosensitizers with Long-Lived Triplet States. Inorg. Chem. 2022, 61, 214–226. 10.1021/acs.inorgchem.1c02771. 34908410

[ref71] Wang L.; Monro S.; Cui P.; Yin H.; Liu B.; Cameron C. G.; Xu W.; Hetu M.; Fuller A.; Kilina S.; McFarland S. A.; Sun W. Heteroleptic Ir(III)N6 Complexes with Long-Lived Triplet Excited States and in Vitro Photobiological Activities. ACS Appl. Mater. Interfaces 2019, 11, 3629–3644. 10.1021/acsami.8b14744. 30608121 PMC6355354

[ref72] Wang P.; Dong R.; Guo S.; Zhao J.; Zhang Z.-M.; Lu T.-B. Improving photosensitization for photochemical CO_2_-to-CO conversion. Natl. Sci. Rev. 2020, 7, 1459–1467. 10.1093/nsr/nwaa112. 34691542 PMC8288749

[ref73] Crawford A. G.; Dwyer A. D.; Liu Z.; Steffen A.; Beeby A.; Pålsson L.-O.; Tozer D. J.; Marder T. B. Experimental and Theoretical Studies of the Photophysical Properties of 2- and 2,7-Functionalized Pyrene Derivatives. J. Am. Chem. Soc. 2011, 133, 13349–13362. 10.1021/ja2006862. 21751803

[ref74] Karuppannan S.; Chambron J.-C. Supramolecular Chemical Sensors Based on Pyrene Monomer-Excimer Dual Luminescence. Chem. – Asian J. 2011, 6, 964–984. 10.1002/asia.201000724. 21271681

[ref75] Figueira-Duarte T. M.; Müllen K. Pyrene-Based Materials for Organic Electronics. Chem. Rev. 2011, 111, 7260–7314. 10.1021/cr100428a. 21740071

[ref76] Chercka D.; Yoo S.-J.; Baumgarten M.; Kim J.-J.; Müllen K. Pyrene based materials for exceptionally deep blue OLEDs. J. Mater. Chem. C 2014, 2, 9083–9086. 10.1039/C4TC01801J.

[ref77] Salunke J. K.; Wong F. L.; Feron K.; Manzhos S.; Lo M. F.; Shinde D.; Patil A.; Lee C. S.; Roy V. A. L.; Sonar P.; Wadgaonkar P. P. Phenothiazine and carbazole substituted pyrene based electroluminescent organic semiconductors for OLED devices. J. Mater. Chem. C 2016, 4, 1009–1018. 10.1039/C5TC03690A.

[ref78] Hallett A. J.; White N.; Wu W.; Cui X.; Horton P. N.; Coles S. J.; Zhao J.; Pope S. J. A. Enhanced photooxidation sensitizers: the first examples of cyclometalated pyrene complexes of iridium(iii). Chem. Commun. 2012, 48, 10838–10840. 10.1039/c2cc35907c. 23026926

[ref79] Constable E. C.; Neuburger M.; Rösel P.; Schneider G. E.; Zampese J. A.; Housecroft C. E.; Monti F.; Armaroli N.; Costa R. D.; Ortí E. Ligand-Based Charge-Transfer Luminescence in Ionic Cyclometalated Iridium(III) Complexes Bearing a Pyrene-Functionalized Bipyridine Ligand: A Joint Theoretical and Experimental Study. Inorg. Chem. 2013, 52, 885–897. 10.1021/ic302026f. 23268720

[ref80] Howarth A. J.; Davies D. L.; Lelj F.; Wolf M. O.; Patrick B. O. Tuning the Emission Lifetime in Bis-cyclometalated Iridium(III) Complexes Bearing Iminopyrene Ligands. Inorg. Chem. 2014, 53, 11882–11889. 10.1021/ic501032t. 25347609

[ref81] Seth S. K.; Purkayastha P. Unusually Large Singlet Oxygen (^1^O_2_) Production by Very Weakly Emissive Pyrene-Functionalized Iridium(III) Complex: Interplay between Excited 3ILCT/3IL and 3MLCT States. Eur. J. Inorg. Chem. 2020, 2020, 2990–2997. 10.1002/ejic.202000442.

[ref82] Wang J.-W.; Huang H.-H.; Wang P.; Yang G.; Kupfer S.; Huang Y.; Li Z.; Ke Z.; Ouyang G. Co-facial π-π Interaction Expedites Sensitizer-to-Catalyst Electron Transfer for High-Performance CO_2_ Photoreduction. JACS Au 2022, 2, 1359–1374. 10.1021/jacsau.2c00073. 35783182 PMC9241016

[ref83] Goze C.; Kozlov D. V.; Tyson D. S.; Ziessel R.; Castellano F. N. Synthesis and photophysics of ruthenium(ii) complexes with multiple pyrenylethynylene subunits. New J. Chem. 2003, 27, 1679–1683. 10.1039/b307327k.

[ref84] Lincoln R.; Kohler L.; Monro S.; Yin H.; Stephenson M.; Zong R.; Chouai A.; Dorsey C.; Hennigar R.; Thummel R. P.; McFarland S. A. Exploitation of Long-Lived 3IL Excited States for Metal-Organic Photodynamic Therapy: Verification in a Metastatic Melanoma Model. J. Am. Chem. Soc. 2013, 135, 17161–17175. 10.1021/ja408426z. 24127659

[ref85] Dierks P.; Päpcke A.; Bokareva O. S.; Altenburger B.; Reuter T.; Heinze K.; Kühn O.; Lochbrunner S.; Bauer M. Ground- and Excited-State Properties of Iron(II) Complexes Linked to Organic Chromophores. Inorg. Chem. 2020, 59, 14746–14761. 10.1021/acs.inorgchem.0c02039. 32935979

[ref86] Wu S.; Huang Z.; Liu S.; Chung P. K. A Pyrene-based Highly Selective Turn-On Fluorescent Sensor for Copper(II) Ion and its Application in Live Cell Imaging. J. Fluoresc. 2012, 22, 253–259. 10.1007/s10895-011-0955-7. 21870075

[ref87] McClenaghan N. D.; Leydet Y.; Maubert B.; Indelli M. T.; Campagna S. Excited-state equilibration: a process leading to long-lived metal-to-ligand charge transfer luminescence in supramolecular system. Coord. Chem. Rev. 2005, 249, 1336–1350. 10.1016/j.ccr.2004.12.017.

[ref88] Sell A. C.; Wetzel J. C.; Schmitz M.; Maijenburg A. W.; Woltersdorf G.; Naumann R.; Kerzig C. Water-soluble ruthenium complex-pyrene dyads with extended triplet lifetimes for efficient energy transfer applications. Dalton Trans. 2022, 51, 10799 10.1039/D2DT01157C. 35788236

[ref89] Wanninger-Weißs C.; Wagenknecht H.-A. Synthesis of 5-(2-Pyrenyl)-2′-deoxyuridine as a DNA Modification for Electron-Transfer Studies: The Critical Role of the Position of the Chromophore Attachment. Eur. J. Org. Chem. 2008, 2008, 64–71. 10.1002/ejoc.200700818.

[ref90] Crawford A. G.; Liu Z.; Mkhalid I. A. I.; Thibault M.-H.; Schwarz N.; Alcaraz G.; Steffen A.; Collings J. C.; Batsanov A. S.; Howard J. A. K.; Marder T. B. Synthesis of 2- and 2,7-Functionalized Pyrene Derivatives: An Application of Selective C–H Borylation. Chem. – Eur. J. 2012, 18, 5022–5035. 10.1002/chem.201103774. 22415854

[ref91] Islam M. M.; Hu Z.; Wang Q.; Redshaw C.; Feng X. Pyrene-based aggregation-induced emission luminogens and their applications. Mater. Chem. Front. 2019, 3, 762–781. 10.1039/C9QM00090A.

[ref92] Rowsell J. L. C.; Yaghi O. M. Effects of Functionalization, Catenation, and Variation of the Metal Oxide and Organic Linking Units on the Low-Pressure Hydrogen Adsorption Properties of Metal–Organic Frameworks. J. Am. Chem. Soc. 2006, 128, 1304–1315. 10.1021/ja056639q. 16433549

[ref93] Wan S.; Guo J.; Kim J.; Ihee H.; Jiang D. A Belt-Shaped, Blue Luminescent, and Semiconducting Covalent Organic Framework. Angew. Chem., Int. Ed. 2008, 47, 8826–8830. 10.1002/anie.200803826. 18830952

[ref94] Sun J.; Zhao J.; Guo H.; Wu W. Visible-light harvesting iridium complexes as singlet oxygen sensitizers for photooxidation of 1,5-dihydroxynaphthalene. Chem. Commun. 2012, 48, 4169–4171. 10.1039/c2cc16690a. 22301532

[ref95] Manav N.; Kesavan P. E.; Ishida M.; Mori S.; Yasutake Y.; Fukatsu S.; Furuta H.; Gupta I. Phosphorescent rhenium-dipyrrinates: efficient photosensitizers for singlet oxygen generation. Dalton Trans. 2019, 48, 2467–2478. 10.1039/C8DT04540B. 30694280

[ref96] Tanaka S.; Enoki T.; Imoto H.; Ooyama Y.; Ohshita J.; Kato T.; Naka K. Highly Efficient Singlet Oxygen Generation and High Oxidation Resistance Enhanced by Arsole-Polymer-Based Photosensitizer: Application as a Recyclable Photooxidation Catalyst. Macromolecules 2020, 53, 2006–2013. 10.1021/acs.macromol.9b02620.

[ref97] Kinzel T.; Zhang Y.; Buchwald S. L. A New Palladium Precatalyst Allows for the Fast Suzuki–Miyaura Coupling Reactions of Unstable Polyfluorophenyl and 2-Heteroaryl Boronic Acids. J. Am. Chem. Soc. 2010, 132, 14073–14075. 10.1021/ja1073799. 20858009 PMC2953245

[ref98] Nycz J. E.; Wantulok J.; Sokolova R.; Pajchel L.; Stankevič M.; Szala M.; Malecki J. G.; Swoboda D. Synthesis and Electrochemical and Spectroscopic Characterization of 4,7-diamino-1,10-phenanthrolines and Their Precursors. Molecules 2019, 24, 4102 10.3390/molecules24224102. 31766294 PMC6891714

[ref99] Auerhammer N.; Schulz A.; Schmiedel A.; Holzapfel M.; Hoche J.; Röhr M. I. S.; Mitric R.; Lambert C. Dynamic exciton localisation in a pyrene-BODIPY-pyrene dye conjugate. Phys. Chem. Chem. Phys. 2019, 21, 9013–9025. 10.1039/C9CP00908F. 30931442

[ref100] Lennox A. J. J.; Lloyd-Jones G. C. Selection of boron reagents for Suzuki-Miyaura coupling. Chem. Soc. Rev. 2014, 43, 412–443. 10.1039/C3CS60197H. 24091429

[ref101] Haedler A. T.; Misslitz H.; Buehlmeyer C.; Albuquerque R. Q.; Köhler A.; Schmidt H.-W. Controlling the π-Stacking Behavior of Pyrene Derivatives: Influence of H-Bonding and Steric Effects in Different States of Aggregation. ChemPhysChem 2013, 14, 1818–1829. 10.1002/cphc.201300242. 23619937

[ref102] Giereth R.; Frey W.; Junge H.; Tschierlei S.; Karnahl M. Copper Photosensitizers Containing P^N Ligands and Their Influence on Photoactivity and Stability. Chem. – Eur. J. 2017, 23, 17432–17437. 10.1002/chem.201703672. 29024115

[ref103] Jones C. M.; Asher S. A. Ultraviolet resonance Raman study of the pyrene S4, S3, and S2 excited electronic states. J. Chem. Phys. 1988, 89, 2649–2661. 10.1063/1.455015.

[ref104] Karpovich D. S.; Blanchard G. J. Relating the polarity-dependent fluorescence response of pyrene to vibronic coupling. Achieving a fundamental understanding of the py polarity scale. J. Phys. Chem. 1995, 99, 3951–3958. 10.1021/j100012a014.

[ref105] Aguilera-Sigalat J.; Sanchez-SanMartín J.; Agudelo-Morales C. E.; Zaballos E.; Galian R. E.; Pérez-Prieto J. Further Insight into the Photostability of the Pyrene Fluorophore in Halogenated Solvents. ChemPhysChem 2012, 13, 835–844. 10.1002/cphc.201100843. 22271708

[ref106] Shirdel J.; Penzkofer A.; Procházka R.; Shen Z.; Strauss J.; Daub J. Absorption and emission spectroscopic characterisation of a pyrene-flavin dyad. Chem. Phys. 2007, 331, 427–437. 10.1016/j.chemphys.2006.11.014.

[ref107] Bains G. K.; Kim S. H.; Sorin E. J.; Narayanaswami V. The Extent of Pyrene Excimer Fluorescence Emission Is a Reflector of Distance and Flexibility: Analysis of the Segment Linking the LDL Receptor-Binding and Tetramerization Domains of Apolipoprotein E3. Biochemistry 2012, 51, 6207–6219. 10.1021/bi3005285. 22779734 PMC3448802

[ref108] Cuttell D. G.; Kuang S.-M.; Fanwick P. E.; McMillin D. R.; Walton R. A. Simple Cu(I) Complexes with Unprecedented Excited-State Lifetimes. J. Am. Chem. Soc. 2002, 124, 6–7. 10.1021/ja012247h. 11772046

[ref109] Yang Y.; Doettinger F.; Kleeberg C.; Frey W.; Karnahl M.; Tschierlei S. How the Way a Naphthalimide Unit is Implemented Affects the Photophysical and -catalytic Properties of Cu(I) Photosensitizers. Front. Chem. 2022, 10, 936863 10.3389/fchem.2022.936863. 35783217 PMC9247301

[ref110] Indelli M. T.; Ghirotti M.; Prodi A.; Chiorboli C.; Scandola F.; McClenaghan N. D.; Puntoriero F.; Campagna S. Solvent Switching of Intramolecular Energy Transfer in Bichromophoric Systems: Photophysics of (2,2′-Bipyridine)tetracyanoruthenate(II)/ Pyrenyl Complexes. Inorg. Chem. 2003, 42, 5489–5497. 10.1021/ic034185x. 12950195

[ref111] Epelde-Elezcano N.; Martínez-Martínez V.; Peña-Cabrera E.; Gómez-Durán C. F. A.; Arbeloa I. L.; Lacombe S. Modulation of singlet oxygen generation in halogenated BODIPY dyes by substitution at their meso position: towards a solvent-independent standard in the vis region. RSC Adv. 2016, 6, 41991–41998. 10.1039/C6RA05820E.

[ref112] Godard J.; Brégier F.; Arnoux P.; Myrzakhmetov B.; Champavier Y.; Frochot C.; Sol V. New Phenalenone Derivatives: Synthesis and Evaluation of Their Singlet Oxygen Quantum Yield. ACS Omega 2020, 5, 28264–28272. 10.1021/acsomega.0c04172. 33163810 PMC7643266

[ref113] Schmidt R.; Tanielian C.; Dunsbach R.; Wolff C. Phenalenone, a universal reference compound for the determination of quantum yields of singlet oxygen O_2_(^1^Δ_g_) sensitization. J. Photochem. Photobiol., A 1994, 79, 11–17. 10.1016/1010-6030(93)03746-4.

[ref114] Gallavardin T.; Armagnat C.; Maury O.; Baldeck P. L.; Lindgren M.; Monnereau C.; Andraud C. An improved singlet oxygen sensitizer with two-photon absorption and emission in the biological transparency window as a result of ground state symmetry-breaking. Chem. Commun. 2012, 48, 1689–1691. 10.1039/C2CC15904J. 22182988

[ref115] Kaeser A.; Mohankumar M.; Mohanraj J.; Monti F.; Holler M.; Cid J.-J.; Moudam O.; Nierengarten I.; Karmazin-Brelot L.; Duhayon C.; Delavaux-Nicot B.; Armaroli N.; Nierengarten J.-F. Heteroleptic Copper(I) Complexes Prepared from Phenanthroline and Bis-Phosphine Ligands. Inorg. Chem. 2013, 52, 12140–12151. 10.1021/ic4020042. 24083360

[ref116] Fischer S.; Hollmann D.; Tschierlei S.; Karnahl M.; Rockstroh N.; Barsch E.; Schwarzbach P.; Luo S.-P.; Junge H.; Beller M.; Lochbrunner S.; Ludwig R.; Brückner A. Death and Rebirth: Photocatalytic Hydrogen Production by a Self-Organizing Copper-Iron System. ACS Catal. 2014, 4, 1845–1849. 10.1021/cs500387e.

[ref117] Lennox A. J. J.; Fischer S.; Jurrat M.; Luo S.-P.; Rockstroh N.; Junge H.; Ludwig R.; Beller M. Copper-Based Photosensitisers in Water Reduction: A More Efficient In Situ Formed System and Improved Mechanistic Understanding. Chem. – Eur. J. 2016, 22, 1233–1238. 10.1002/chem.201503812. 26691442

[ref118] Zhang Y.-Y.; Ni Z.-J.; Elam E.; Zhang F.; Thakur K.; Wang S.; Zhang J.-G.; Wei Z.-J. Juglone, a novel activator of ferroptosis, induces cell death in endometrial carcinoma Ishikawa cells. Food Funct. 2021, 12, 4947–4959. 10.1039/D1FO00790D. 34100505

