# Supplementary material for: Bichromophoric Photosensitizers: How and Where to Attach Pyrene Moieties to Phenanthroline to Generate Copper(I) Complexes
Source: Inorg Chem. 2023 May 18;62(21):8166–78. doi: 10.1021/acs.inorgchem.3c00482 (PMC10230506; doi:10.1021/acs.inorgchem.3c00482)
Supplement: Supplementary file 1 — ic3c00482_si_001.pdf [file ic3c00482_si_001.pdf]

Supporting Information

Bichromophoric Photosensitizers: How and Where to Attach Pyrene Moieties to Phenanthroline to Generate Copper(I) Complexes

Florian Doettinger[1†], Yingya Yang[1†], Michael Karnahl[1], Stefanie Tschierlei[1*]*

[1] Department of Energy Conversion, Institute of Physical and Theoretical Chemistry, Technische Universität Brauschweig, Rebenring 31, 38106 Braunschweig, Germany

† These authors have contributed equally to this work and share first authorship.
*Correspondence:

Dr. Michael Karnahl: michael.karnahl@tu-bs.de
Prof. Dr. Stefanie Tschierlei: s.tschierlei@tu-bs.de

Electronic Supplementary Information - Table of Contents

1	Experimental Details	page	S1
2	Synthetic Details	page	S4
3	NMR Spectra	page	S12
4	MS Spectra	page	S23
5	Electrochemical Data	page	S29
6	(Time-Dependent) Density Functional Theory (TD-)DFT	page	S33
7	UV/vis Absorption	page	S41
8	Steady-State Emission	page	S42
9	Transient Absorption	page	S47
10	Singlet Oxygen Generation	page	S50
11	Photostability	page	S52
12	Photooxidation of 1,5-Dihydroxynaphthalene	page	S53
	References	page	S55

1 Experimental Details

NMR spectroscopy. Nuclear magnetic resonance (NMR) measurements were performed by the analytical service of the Institute of Inorganic and Analytical Chemistry at the Technische Universität Braunschweig at 298 K with a Bruker Avance IIIHD 500 spectrometer operating at frequencies of 500 MHz (^{1}H), 126 MHz (^{13}C) and 203 MHz (^{31}P). The spectra were then processed using the TopSpin software (version 4.1.1). The solvent used for each measurement is indicated at the corresponding NMR data. All spectra are referenced against the deuterated solvent as internal standard. The chemical shifts δ are denoted in ppm relative to the residual solvent signal of the deuterated solvent. NMR multiplicities are denoted as: *s* (singlet), *d* (doublet), *t* (triplet), *q* (quartet), *m* (multiplet). Coupling constants *J* are given in Hz.

Mass spectrometry. Mass spectrometric (MS) measurements were performed by the analytical service of the Institute of Inorganic and Analytical Chemistry at the Technische Universität Braunschweig. High resolution mass spectra have been measured using electrospray ionization (ESI) on a LTQ-Orbitrap Velos orbitrap mass spectrometer from ThermoFisher Scientific. Samples were dissolved in methanol spiked with 0.1 mg/mL tetradecyltrimethylammonium bromide. MS values are given as m/z.

Electrochemistry. Cyclic voltammetry was measured in ambient dimethylformamide with 0.1 M Bu_4NPF_6 as the supporting electrolyte. Measurements were performed on an Autolab potentiostat PGSTAT204 from Metrohm using a three-electrode configuration with a glassy carbon disc with 3 mm diameter stick working electrode, a platinum wire counter electrode, and a non-aqueous Ag/Ag^+ reference electrode (with 0.01 M $AgNO_3$ in acetonitrile). All data are referenced against the ferrocene/ferricenium (Fc/Fc^+) couple, by adding ferrocene to the solution after each measurement. The scan rate usually was 100 mV/s unless stated otherwise.

Steady-state UV/vis absorption. Spectra were measured with a JASCO V-770 spectrophotometer. To that, the compounds were dissolved in spectroscopic grade acetonitrile and the spectra recorded applying a standard 10 mm fluorescence quartz glass cuvette.

Steady-state emission. Spectra were recorded with a Horiba Jobin-Yvon FluoroMax Plus-C emission spectrometer. All samples were measured in spectroscopic grade acetonitrile and dichloromethane solution under inert conditions (unless denoted otherwise) using a sealed 10 mm fluorescence quartz glass cuvette. Optical densities (OD) were around 0.1 (or below) at the respective excitation wavelength. The emission quantum yield of the compounds were calculated with pyrene (φ = 0.62) in inert acetonitrile and pyrene (φ = 0.38) in inert dichloromethane with excitation wavelength at 334 nm as reference.[1,2]

Photostability tests. UV/vis absorption spectra were acquired with an Avantes AvaSpec-ULS2048CL spectrophotometer. A 150 W xenon lamp (LOT-QuantumDesign GmbH, LSE140/160.25C) was used as light source and 0.5 OD filter was introduced for the measurements. The measurements were carried out under oxygen free conditions by using dry acetonitrile and dichloromethane and under aerated conditions. The samples were prepared using the same method as for the absorption and emission measurements applying sealed quartz glass cuvettes with a path length of 10 mm.

Emission lifetime. Emission lifetimes were acquired using a Q-switched pulsed Nd:YAG laser system. The excitation pulses were centered at 355 nm with a pulse duration of approximately 6 ns with a beam power of approximately 1.0 mJ per pulse directly at the sample holder. Emission was detected using a photo multiplier tube at the Edinburgh Instruments LP980 spectrometer at the respective emission maximum of the sample. All emission lifetimes were recorded either under oxygen free conditions in dry acetonitrile and dichloromethane in 10 mm fluorescence quartz glass cuvettes at room temperature. The respective solutions had an optical density of approximately 0.1 at the excitation wavelength.

Nanosecond transient absorption spectroscopy. Excitation pulses were generated using a Q-switched pulsed Nd:YAG laser (Q-smart 450mJ, Quantel laser) with an output centered at 355 nm (approx. 6 ns pulse duration, repetition rate of 10 Hz). The pulses were passed through a laser line filter (CWL = 355 ± 2 nm, FWHM = 10 ± 2 nm) to ensure that the samples were only excited by 355 nm light. The power of the pump beam was about 3 mJ per pulse at the sample. The stability of the sample was verified by means of UV/vis spectra before and after each measurement. The spectrometer used was a LP980-K spectrometer from Edinburgh Instruments, where the pump and probe beams spatially overlapped at the sample position in a perpendicular beam setup. The probe lamp was operated in flash mode (150 W ozone-free xenon arc lamp, 30 A). After passing the sample the probe light was recorded using a photo multiplier tube (Hamamatsu R928P). A standard fused silica cuvette with a layer thickness of 10 mm and a sample OD of 0.2~0.3 at the pump wavelength was used in this setup. The compounds were dissolved in dry acetonitrile and dichloromethane under inert conditions.

Singlet oxygen measurement. For evaluation of the singlet oxygen quantum yield, the phosphorescence of $^1O_2$ at approximately 1276 nm was detected with a Horiba Jobin-Yvon FluoroMax Plus-C spectrofluorometer equipped with a 150 W Xe arc excitation lamp, a liquid-nitrogen cooled DSS-IGA020L InGaAs photodiode detector (800- 1550 nm) and Czerny-Turner monochromators with NIR grating blazed at 1000 nm. Absorption spectroscopy was measured with a JASCO Spectrometer V-770 and was done before and after each singlet oxygen measurement. Thus, stability of the respective complex on the timescale of the measurements was ensured. For each sample, emission spectra were recorded upon excitation at 407 nm. As a reference the whole procedure was repeated for the known standard phenalenone. The detected singlet oxygen emissions were baseline corrected at 1350 nm. The area below the signal was integrated. The respective singlet oxygen quantum yield $\varphi(^1O_2)$ was calculated and referenced against the literature reported value for phenalenone of Φ = 1 in acetonitrile.[2,3] The following equation was used:

$$\Phi_c = \Phi_R \left(\frac{A_R}{A_C}\right)\left(\frac{I_C}{I_R}\right)$$

In this equation, Φ_C is the $^1O_2$ quantum yield of the compound and Φ_R is the $^1O_2$ quantum yield of reference (phenalenone), A_C the absorbance of compound and A_R the absorbance of reference, I_C the integral of the singlet oxygen emission of the compound and I_R the integral of the singlet oxygen emission of the reference. This was done for all two different optical densities and the final quantum yield was calculated as the average.[3,4,5,6]

Continuous measurement of $^1O_2$ production was carried out in a 10 mm quartz cuvette with only half-filled acetonitrile solution, taking care to ensure that sufficient air/O_2 was present inside. Between every record of the characteristic emission of $^1O_2$ the covered cuvette was vigorously shaken three times and the absorption spectra of the sample were obtained to

ensure its photostability. For better comparison between the individual measurements, the emission spectra were fitted with a Gaussian function and then the area below the signal was integrated (Figure S1.1).

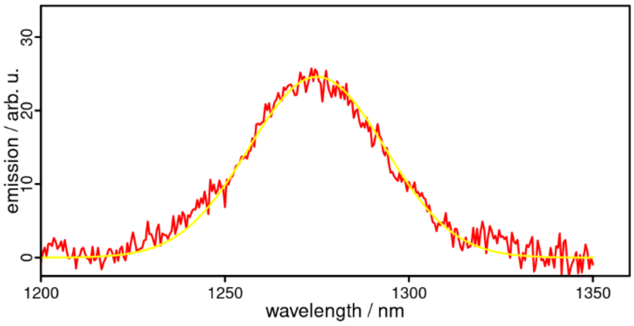

Figure S1. $^{1}O_2$ emission spectrum (rot) after baseline correlation and its fitting with a Gaussian function (yellow).

Photooxidation Measurements. The samples were prepared with an acetonitrile solution containing 1,5-dihydroxynaphthalene (DHN) (1.0×10^{-4} M) and a photosensitizer (1.0×10^{-5} M). Under irradiation (with a 150 W xenon lamp (LOT-QuantumDesign GmbH, LSE140/160.25C) with a 0.5 OD filter and a 380 nm long pass filter between the sample and the lamp) the absorption spectra were recorded with an Avantes AvaSpec-ULS2048CL spectrophotometer every 30 seconds for 3 hours. The decrease in the absorption at 301 nm was monitored for the DHN consumption and the increase in the absorption at 427 nm for the production of juglone. The concentration of DHN was calculated by using its attenuation coefficient (ε = 7664 M^{-1} cm^{-1}) at 301 nm and the concentration of juglone was calculated by using its attenuation coefficient (ε = 3811 M^{-1} cm^{-1}). The yield of juglone was obtained by dividing the concentration of juglone with the initial concentration of DHN.[7,8]

DFT calculations. Quantum chemical calculations at the density functional theory (DFT) level were performed using the ORCA program package (Version 5.0.3).[9] Geometry optimizations of the electronic ground state were conducted using the BP86[10] exchange-correlation functional for pre-optimization. Then, the PBE0[11] hybrid functional was used for final optimization and for TD-DFT calculations concerning theoretical UV/vis spectra and difference density plots. To account for dispersion effects, the D3 correction by S. Grimme including the Becke-Johnson (BJ) damping[12,13] was used. As basis sets the Karlsruhe's valence triple-zeta polarization functions basis sets (def2-TZVP) were applied.[14,15] Solvation effects were accounted for by the conductor-like polarizable continuum model, CPCM, for acetonitrile and dichloromethane.[16]
Optimized geometries were verified as minima on the potential energy surface by frequency calculations (analytical, PBE0-D3(BJ)/def2-tzvp, CPCM). Visualizations of the B3LYP molecular orbitals and of the electron difference density plots were evoked using the Chemcraft software package (Version 1.8).[17]

2 Synthetic Details

Chemicals were acquired from commercial suppliers (e.g. Sigma-Aldrich, VWR, Acros Organics or ABCR) and directly used as received, if not specified otherwise. Precursors, catalysts, ligands and complexes were synthesized according to the procedures described herein or in the cited literature.
Solvents were purified and dried according to standard procedures. Dry dichloromethane (DCM) used for complexation and dry *n*-hexane used for synthesis were purified by distillation over CaH_2 under argon atmosphere. Oxygen free tetrahydrofurane (THF), *n*-hexane and water (H_2O) were prepared by bubbling through with argon (2 h for 50 mL while stirring – for THF and *n*-hexane additionally cooling to 0 °C).
Reaction with oxygen- and/or water-sensitive compounds were carried out in dried glassware and under argon atmosphere. Glassware was vacuum dried while heated with a heat gun at 500 °C for several minutes and flushed with argon three times.
Pyrene used for synthesis and as reference standard for spectroscopic measurements was purified by filtering a concentrated DCM solution through a plug of celite at ambient conditions and evaporating the solvent subsequently.

Synthesis of 4,4,5,5-tetramethyl-2-pyren-2-yl-1,3,2-dioxaborolane (Pyr2Pin)

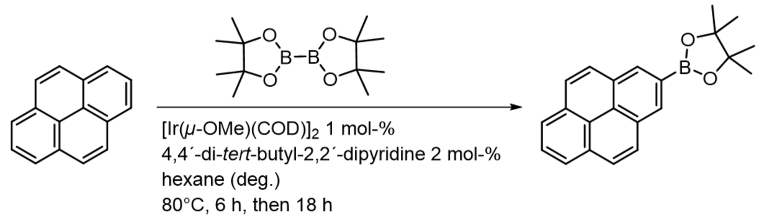

In a Schlenk flask Pyrene (2.22 g, 11.0 mmol), [{Ir(μ-OMe)(cod)}$_2$] (72.8 mg, 0.11 mmol) and 4,4′-di-tert-butyl-2,2′-dipyridine (58.9 mg, 0.22 mmol) were suspended in degassed hexane (80 ml) and the mixture heated to 80 °C subsequently (*mixture turns darker when heating*).
A solution of bis(pinacolato)diboron (2.65 g, 10.4 mmol) in degassed hexane (80 ml) was added dropwise at 80 °C while stirring vigorously during 6 h (*an automatic syringe pump was used at 13.3 ml/h*). The reaction mixture was reacted for another 18 h at 80 °C and then allowed to cool to room temperature (*precipitation occurs from a dark reddish solution*).
The solvent was removed and the mixture dissolved in DCM to pass the solution through a plug of celite. The solvent was removed again and the remaining solid was purified by flash column chromatography (Silica, DCM:hexane = 1:1). (*The fractions can be visualized using a UV lamp. The second (from two) fluorescing fraction (blue-green) is product*)
Yield: 1.02 g (28.1%, 3.1 mmol) of a white solid.

^{1}H-NMR: δ [ppm] = 8.64 (*s*, 2H), 8.17 (*d*, *J* = 7.57 Hz, 2H), 8.11 (*d*, *J* =9.10 Hz, 2H), 8.06 (*d*, *J* = 8.98 Hz, 2H), 8.02 (*dd*, *J* = 8.20, *J* = 7.15 Hz, 1H), 1.47 (*s*, 12H).

Comment: It should be noted that the synthesis of **Pyr2Pin** proceeded with significantly lower yields compared to the literature (30%, 29% and 27% *vs.* 58%).
Although two new badges of dimeric Ir-catalyst [{Ir(μ-OMe)(cod)}$_2$] or dry and degassed *n*-hexane (instead of degassed *n*-hexane) were used, no improvement could be invoked. Utilizing an automatic syringe pump for precisely controlled addition of the bis(pinacolato)diboron solution over six hours did also not change the outcome significantly.

Analysis of the three fractions obtained after column chromatography revealed a ratio of 2:2:3 between pyrene, **Pyr2Pin** and the side product substituted at the 2- and 7-position. Still the described procedure successfully yielded the desired **Pyr2Pin** in acceptable yield and in high purity in a straight forward approach.

Synthesis of 2,9-Dimethyl-4,7-di(pyren-1-yl)-1,10-phenanthroline (Pyr1)

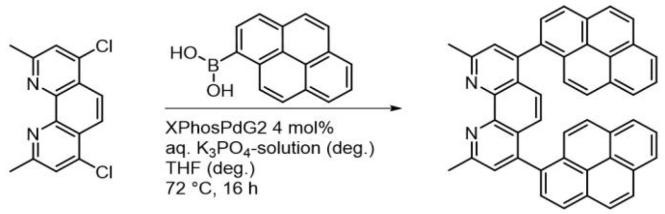

A 50 ml round bottomed flask equipped with a magnetic stir bar and XPhos-Pd-G2 precatalyst (20 mg, 0.026 mmol, 4 mol-%), **Neo-4,7-Cl$_2$** (180 mg, 0.65 mmol, 1 eq.) and **Pyr1BA** (479 mg, 1.95 mmol, 3 eq.). The vessel was attached to a reflux cooler and was evacuated and backfilled with argon subsequently (this was repeated two times further).
Degassed THF (10 mL) was added via syringe. Then, degassed 0.5 M aqueous K_3PO_4 solution (12 mL) was added via syringe and the reaction was vigorously stirred at 72 °C for 16 hours.
THF was evaporated at a rotary evaporator and DCM (10 ml) was added and the mixture stirred for 5 minutes. The phases were separated, the organic layer washed with NaOH solution (1 M) two times, treated with brine and finally dried over $MgSO_4$. The solvent was evaporated and the remaining red-brown oil purified via chromatography (basic aluminum oxide, DCM).
Remaining impurities of pyrene were removed by conducting additional chromatography (Silica, DCM then DCM + 4% MeOH) (*the product which appears yellow-orange on silica elutes with the additional MeOH*).
The solvent was evaporated and the product dried in *vacuo* at 40 °C to yield 218 mg (0.35 mmol, 55%) of pure compound **Pyr1**.

^{1}H-NMR: δ [ppm] = 8.23 (*d*, *J* = 7.9 Hz, 2H), 8.20 (*ddd*, *J* = 3.1, 7.6 Hz, 0.9 Hz, 2H), 8.10 (*m*, 6 H), 7.96 (*m*, 6), 7.68 (*s*, 2H), 7.59 (*dd*, *J* = 37.5 Hz, 9.2 Hz), 7.13 (*d*, *J* = 17.4 Hz, 2H), 3.16 (*s*, 6H). **^{13}C-NMR**: δ [ppm] = 159.0, 131.4, 131.4, 131.3, 130.8, 130.8, 129.2, 128.2, 128.0, 127.5, 127.3, 126.5, 126.3, 125.6, 125.4, 124.8, 124.7, 124.6, 124.5, 124.5, 124.4, 123.8, 26.0. **DEPT-NMR**: δ [ppm] = 128.2, 128.0, 127.5, 127.3, 126.3, 125.6, 125.4, 124.8, 124.5, 124.4, 123.8, 26.0. **HRMS** (ESI) *m/z*: calcd. for $[C_{46}H_{28}N_2H]^+$: 609.2325, found: 609.2238.

Synthesis of 2,9-Dimethyl-4,7-di(pyren-2-yl)-1,10-phenanthroline (Pyr2)

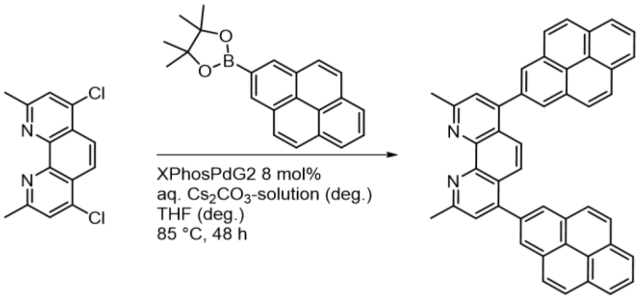

A 50 ml round bottomed flask equipped with a magnetic stir bar and XPhos-Pd-G2 precatalyst (33 mg, 0.043 mmol, 8 mol-%), **Neo-4,7-Cl₂** (150 mg, 0.54 mmol, 1 eq.), **Pyr2Pin** (621 mg, 1.89 mmol, 3.5 eq.) and Cs_2CO_3 (3.5 g, 10.8 mmol, 20 eq.). The vessel was attached to a reflux cooler and was evacuated and backfilled with argon subsequently (this was repeated two times further).

Degassed THF (12 mL) and degassed water (10 mL) were added via syringe and the reaction was stirred vigorously at 85 °C for 48 hours.

Then, THF was evaporated at a rotary evaporator and the remaining aqueous phase was extracted with $CHCl_3$ to give a clear red organic phase. The solution was passed through small column fill with basic aluminium oxide (eluent: $CHCl_3$ + 1% MeOH) to yield a mildly orange solution which was subsequently washed with water three times and finally with brine.

The crude product was slowly recrystallized from boiling toluene to yield a white-grey solid. The product was finally dissolved in $CHCl_3$ and filtered through wool to obtain 160 mg (0.26 mmol, 49%) of pure compound **Pyr2**.

^{1}H-NMR: δ [ppm] = 8.34 (*s*, 4H), 8.23 (*d*, *J* = 7.6, 4H), 8.15 (*d*, *J* = 9.1, 4H), 8.12 (*d*, *J* = 9.1, 2H), 8.05 (*t*, *J* = 7.6 Hz, 2H), 7.88, (*s*, 2H) 7.73 (*s*, 2H), 3.15 (*s*, 6H). **^{13}C-NMR**: δ [ppm] = 158.9, 131.3, 131.2, 128.4, 127.2, 126.4, 125.8, 125.5, 125.3, 124.4, 123.6, 25.8. **DEPT-NMR**: δ [ppm] = 128.4, 127.2, 126.4, 125.8, 125.5, 123.6, 25.8. **HRMS** (ESI) *m/z*: calcd. for $[C_{46}H_{28}N_2H]^+$: 609.2325, found: 609.2236.

Synthesis of 2,9-Dimethyl-5,6-di(pyren-2-yl)-1,10-phenanthroline (Pyr 3)

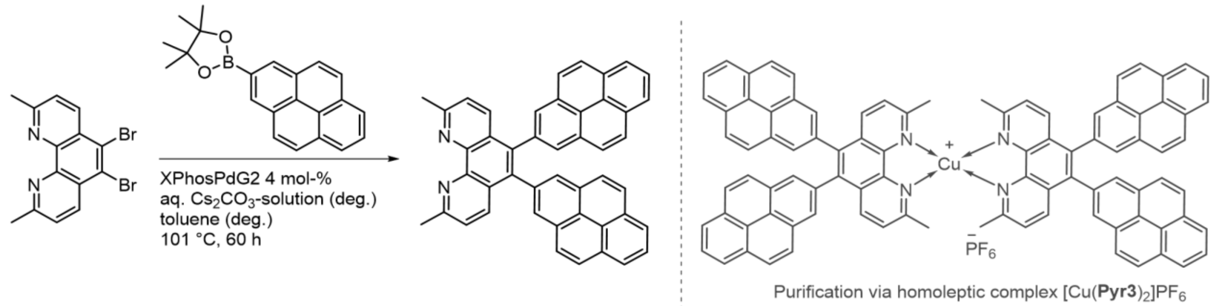

A 50 ml round bottomed flask equipped with a magnetic stir bar and XPhos-Pd-G2 precatalyst (22 mg, 0.028 mmol, 8 mol-%), **Neo-5,6-Br₂** (130 mg, 0.36 mmol, 1 eq.), **Pyr2Pin** (466 mg, 1.42 mmol, 4 eq.) and Cs_2CO_3 (2.31 g, 7.10 mmol, 20 eq.). The vessel was attached to a reflux cooler and was evacuated and backfilled with argon subsequently (this was repeated two times further).

Degassed toluene (16 mL) was added via syringe. Then, degassed water (8 mL) was added via syringe and the reaction was stirred vigorously at 101 °C for 60 hours.

The phases were separated and the aqueous phase extracted with toluene two times (5 mL each). The organic phases were combined and the solvent was evaporated at a rotary evaporator. The remaining oil was dissolved in $CHCl_3$ and the solution was washed two times with HCl solution (1 M) and saturated Na_2CO_3 solution, subsequently. The organic phase was dried with brine and the solvent was evaporated.

The crude product was again dissolved in $CHCl_3$ (*red, brown turbid solution*) and passed through a short plug of basic aluminum oxide to obtain a clear colourless solution. The solvent was evaporated to yield 240 mg of pale brown foamy solid in already acceptable purity.

To further improve the compounds purity, under inert atmosphere, the compound (230 mg, 0.36 mmol, 2 eq.) was added to solid $[(CH_3CN)_4Cu]PF_6$ (67 mg, 0.18 mmol, 1 eq.) and the mixture was dissolved in degassed CH_2Cl_2 (20 mL). The red solution was stirred at 40 °C for 1 hour and the solution allowed to cool.

The crude target complex was precipitated by adding *n*-hexane, filtered off and dried. The deep red crude complex was purified via column chromatography (silica, CH_2Cl_2, then CH_2Cl_2 + 1.5 % MeOH) (*impurities elute first, the red complex fraction elutes when adding MeOH*).

The solvent was evaporated and the complex dissolved in CH_2Cl_2 (15 mL) and then treated with a basic KCN solution (100 mg KCN, 1.44 mmol, 4 eq., 0.1 M NaOH aqueous solution, 40 mL) while stirring vigorously for 2 hours (*the colour of the organic phase gradually changes from deep red to a slightly pale solution*). The phases were separated.

- - WARNING: POTASSIUM CYANIDE IS EXTREMELY TOXIC! SOLUTIONS MUST BE KEPT BASIC AT ALL TIMES! THE BASIC AQUEOUS SOLUTION WAS QUENCHED WITH H_2O_2 DIRECTLY AFTER WORK-UP - -

The organic phase was washed with water three times (5 mL each), then treated with brine and the solvent evaporated. The resulting product was dried in high vacuum at 50 °C to yield the pale brown solid compound **Pyr3** (119 mg, 0.20 mmol, 55%).

^{1}H-NMR: δ [ppm] = 8.13 (*s*, 4H), 8.07 (*d*, *J* = 7.7 Hz, 4H), 7.94 (*d*, *J* = 8.9 Hz, 4H), 7.90 (*t*, *J* = 7.7 Hz, 4H), 7.89 (*d*, *J* = 9.0 Hz, 2H), 7.72(*d*, *J* = 8.6 Hz, 2H), 7.36 (*d*, *J* = 8.6 Hz, 2H), 2.92 (*s*, 6H). **^{13}C-NMR**: δ [ppm] = 159.0, 145.4, 137.1, 136.4, 136.0, 131.3, 131.0, 128.0, 127.9, 127.8, 127.4, 126.3, 125.4, 124.5, 123.7, 123.6, 25.6. **DEPT-NMR**: δ [ppm] = 135.8, 127.8, 127.7, 127.2, 126.1, 125.2, 123.5, 25.4. **HRMS** (ESI) *m/z*: calcd. for $[C_{46}H_{28}N_2H]^+$: 609.2325, found: 609.2243.

Syntheses of the Complexes CuPyr1-3

The syntheses of **CuPyr1**, **CuPyr2** and **CuPyr3** were conducted following a well-known procedure similar to previous reports.

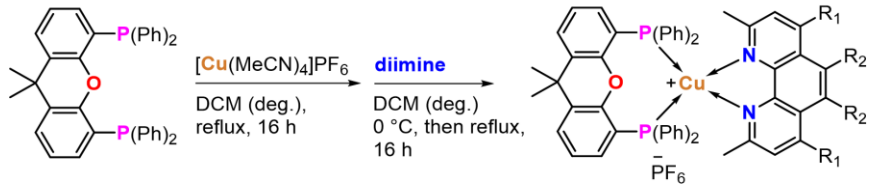

**CuPyr1**		**CuPyr2**		**CuPyr3**	
R_1	R_2	R_1	R_2	R_1	R_2
**1-pyr.**	**H**	**2-pyr.**	**H**	**H**	**2-pyr.**

Notes: Precisely controlled addition of the ligand CH_2Cl_2 solution was realized using an automatic syringe pump. In the reaction towards **CuPyr2**, **Pyr2** was added as a solid under inert conditions at -20 °C due to its low solubility (*vide infra*).

Synthesis of [Cu(Pyr1)(xant)]PF_6 - CuPyr1

A schlenk-tube was equipped with a magnetic stir bar and tetrakis(acetonitrile)copper(I) hexafluorophosphate and xantphos ((9,9-dimethyl-9H-xanthene-4,5-diyl)bis (diphenyl-phosphane)) were added. The vessel was attached to a reflux cooler and the whole apparatus was flushed with argon three times. Dry and degassed dichloromethane (20 mL) was added and the solution was refluxed for 16 hours.

The solution was then cooled to 0 °C and a solution of the respective ligand in dry and degassed dichloromethane (20 mL) was added drop wise very slowly (using a syringe pump at ca. 12 ml/h). After 30 min of stirring at 0 °C the yellow to orange solution was refluxed for additional four hours and then cooled to room temperature.

n-Hexane was slowly added until the point where precipitation initiated. The mixture was then stirred for 30 minutes and another portion of *n*-hexane was added to precipitate more product. The mixture was then stored at -20 °C overnight. After decanting off the clear solution, the red oily precipitate was washed with *n*-hexane and cold diethyl ether and dried under high vacuum to yield 216 mg (77%) of the desired compound.

	m [mg]	M [g/mol)	n [mmol]
[Cu(MeCN)$_4$]PF_6	75.4	372.72	0.200
xantphos	115.7	578.63	0.200
**Pyr1**	121.7	608.74	0.200
Yield **CuPyr1**	216 (77%)	1395.89	0.155

^{1}H-NMR: *δ* [ppm] = 8.35 (*dd*, *J* = 8.0 Hz, 6.4 Hz, 2H), 8.32 (*dd*, *J* = 7.4 Hz, 5.3 Hz, 2H), 8.23 (*m*, 6H), 8.08 (*m*, 4H), 7.98 (*dd*, *J* = 7.9 Hz, 4.2 Hz, 2H), 7.83 (*dd*, *J* = 7.9 Hz, 1.2 Hz, 2H), 7.74 (*s*, 2H), 7.51 (*dd*, *J* = 9.2 Hz, 2.7 Hz, 2H), 7.35 (*m*, 24H), 7.07 (*d*, *J* = 8.3 Hz, 2H), 2.47 (*s*, 6H), 1.79 (*s*, 6H). **^{13}C-NMR**: *δ* [ppm] = 159.5, 156.0, 150.2, 144.5, 135.1, 134.5, 134.2, 134.0, 132.8, 132.3, 131.7, 131.3, 131.1, 129.9, 129.7, 129.3, 129.3, 129.0, 128.6, 128.3, 128.1, 127.8, 127.1, 126.7, 126.5, 125.8, 125.4, 125.3, 125.1, 124.9, 37.0, 28.9, 28.0. **DEPT-NMR**: 134.5, 134.2, 134.0, 131.4, 131.1, 129.9, 129.8, 129.7, 129.4, 129.3, 129.0, 128.6, 128.3, 127.8, 127.1, 126.7, 126.6, 125.9, 125.4, 125.4, 124.9, 28.9, 28.0. **^{31}P-NMR**: *δ* [ppm] = -13.29. **HRMS** (ESI) *m/z*: calcd. for $[C_{85}H_{60}CuN_2OP_2]^+$: 1249.3471, found: 1249.3330.

Synthesis of [Cu(Pyr2)(xant)]PF_6 - CuPyr2

A schlenk-tube was equipped with a magnetic stir bar and tetrakis(acetonitrile)copper(I) hexafluorophosphate and xantphos ((9,9-dimethyl-9H-xanthene-4,5-diyl)bis (diphenylphosphane)) were added. The vessel was attached to a reflux cooler and the whole apparatus was flushed with argon three times. Dry and degassed dichloromethane (25 mL) was added and the solution cooled to -20 °C.
The respective ligand was added as a solid under inert atmosphere and the mixture stirred at -20 °C for 2 h. The solution was allowed to warm to 0 °C and stirred for 1 h. The solution was allowed to for to room temperature while stirring and dry and degassed CH_2Cl_2 (10 mL) was added. The mixture was finally refluxed at 45 °C for 2 h and then cooled to room temperature. *n*-Hexane was slowly added until the point where precipitation initiated. The white precipitate was filtered off to obtain a clear red solution which was again treated with *n*-hexane until the formation of an oil was observed. The mixture was stored at 0 °C overnight. After decanting off the clear solution, the red oily precipitate was washed with *n*-hexane and dried under high vacuum to yield 41 mg (42%) of the desired compound.

	m [mg]	M [g/mol)	n [mmol]
[Cu(MeCN)$_4$]PF_6	26.1	372.72	0.070
xantphos	40.5	578.63	0.070
**Pyr2**	42.6	608.74	0.070
Yield **CuPyr2**	41 (42%)	1395.89	0.029

^{1}H-NMR: *δ* [ppm] = 8.24 (*d*, *J* = 7.7 Hz, 4H), 8.05 (*m*, 6H), 7.94 (*s*, 4H), 7.79 (*d*, *J* = 9.0 Hz, 4H), 7.71 (*dd*, *J* = 7.8 Hz, 1,3 Hz, 2H), 7.41 (*s*, 2), 7.25 (*m*, 8H), 7.09 (*m*, 16H), 7.02 (*m*, 2H), 2.00 (*s*, 6H), 1.65 (*s*, 6H). **^{13}C-NMR**: *δ* [ppm] = 159.1, 155.9, 155.8, 155.8, 150.6, 144.6, 135.0, 134.9, 134.1, 134.1, 134.0, 132.8, 132.6, 132.5, 132.3, 131.4, 131.2, 129.8, 129.8, 129.7, 129.6, 129.1, 128.1, 128.0, 127.4, 126.9, 126.8, 126.6, 125.2, 124.9, 124.4, 122.7, 122.6, 122.5, 37.0, 29.1, 27.8. **DEPT-NMR**: *δ* [ppm] = 134.1, 134.1, 134.0, 131.4, 131.2, 129.8, 129.8, 129.7, 129.6, 129.1, 128.1, 128.0, 127.4, 126.9, 126.8, 126.6, 124.4, 29.1, 27.8. **^{31}P-NMR**: *δ* [ppm] = -12.95. **HRMS** (ESI) *m/z*: calcd. for $[C_{85}H_{60}CuN_2OP_2]^+$: 1249.3471, found: 1249.3481.

Synthesis of [Cu(Pyr3)(xant)]PF_6 - CuPyr3

A schlenk-tube was equipped with a magnetic stir bar and tetrakis(acetonitrile)copper(I) hexafluorophosphate and xantphos ((9,9-dimethyl-9H-xanthene-4,5-diyl)bis (diphenyl-phosphane)) were added. The vessel was attached to a reflux cooler and the whole apparatus was flushed with argon three times. Dry and degassed dichloromethane (8 mL) was added and the solution was refluxed for 16 hours.
The solution was then cooled to 0 °C and a solution of the respective ligand in dry and degassed dichloromethane (8 mL) was added drop wise very slowly (using a syringe pump at ca. 8ml/h). After 30 min of stirring at 0 °C the yellow to orange solution was refluxed for additional four hours and then cooled to room temperature.
n-Hexane was slowly added until the point where precipitation initiated. The mixture was then stirred for 30 minutes and another portion of *n*-hexane was added to precipitate more product. The mixture was then stored at -20 °C overnight. After decanting off the clear solution, the red oily precipitate was washed with *n*-hexane and cold diethyl ether and dried under high vacuum to yield 43 mg (61%) of the desired compound.

	m [mg]	M [g/mol)	n [mmol]
[Cu(MeCN)$_4$]PF_6	18.8	372.72	0.051
xantphos	29.2	578.63	0.051
**Pyr3**	30.7	608.74	0.051
Yield **CuPyr3**	43 (61%)	1395.89	0.031

^{1}H-NMR: δ [ppm] = 8.12 (*s*, 4H), 8.11 (*d*, *J* = 7.5 Hz, 4H), 8.98 (*dd*, *J* = 16.6 Hz, 8.1 Hz, 8H), 7.94 (*t*, *J* = 7.8 Hz, 2H), 7.78 (*d*, *J* = 8.6 Hz, 4H), 7.47 (*m*, 4H), 7.36 (*d*, *J* = 8.6 Hz, 2), 7.28 (*t*, *J* = 7.7 Hz, 2H), 7.20 (*m*, 16H), 7.09 (*m*, 2H), 2.33 (*s*, 6H), 1.74 (*s*, 6H). **^{13}C-NMR**: δ [ppm] = 159.3, 155.6, 143.0, 137.7, 137.6, 135.5, 134.6, 133.7, 132.3, 131.5, 131.3, 131.0, 130.8, 129.4, 128.7, 128.7, 128.6, 127.9, 127.6, 127.1, 126.1, 126.1, 124.4, 123.8, 122.3, 36.7, 28.7, 27.5. **DEPT-NMR**: δ [ppm] = 137.6, 133.7, 131.0, 130.8, 129.4, 128.7, 128.6, 127.9, 127.6, 127.1, 126.1, 126.0, 28.7, 27.5. **^{31}P-NMR**: δ [ppm] = -12.68. **HRMS** (ESI) *m/z*: calcd. for $[C_{85}H_{60}CuN_2OP_2]^+$: 1249.3471, found: 1249.3327.

3 NMR spectra

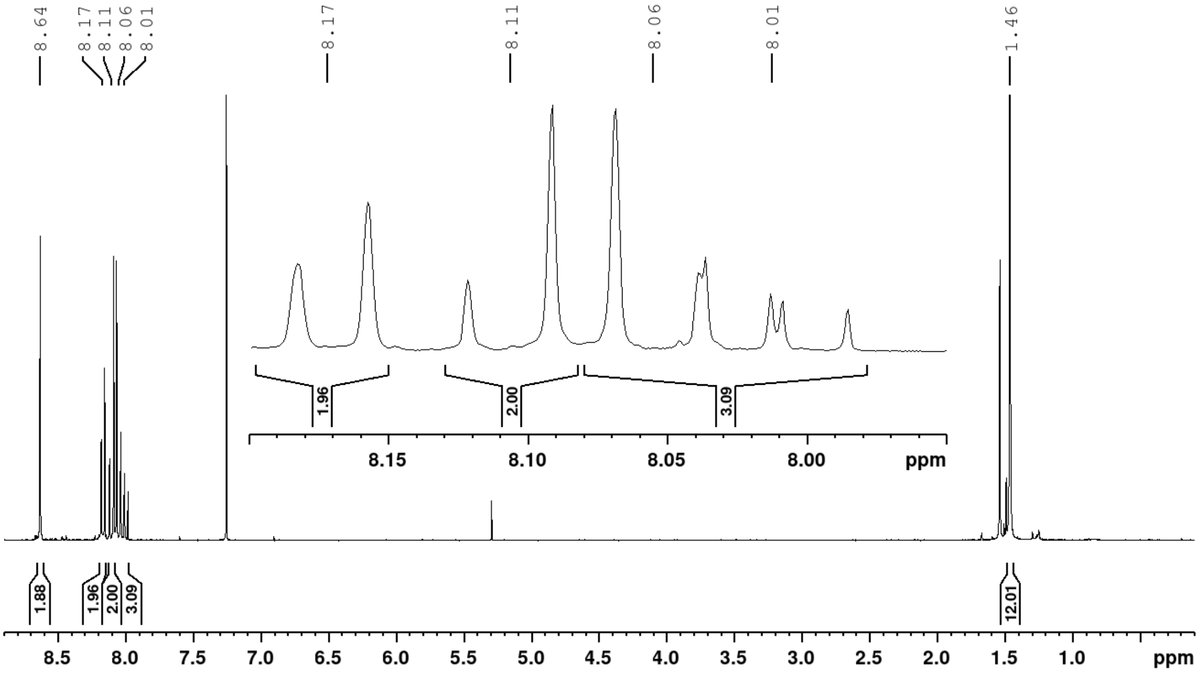

Figure S2. ^{1}H NMR spectrum of 4,4,5,5-tetramethyl-2-pyren-2-yl-1,3,2-dioxaborolane **Pyr2Pin** in $CDCl_3$.

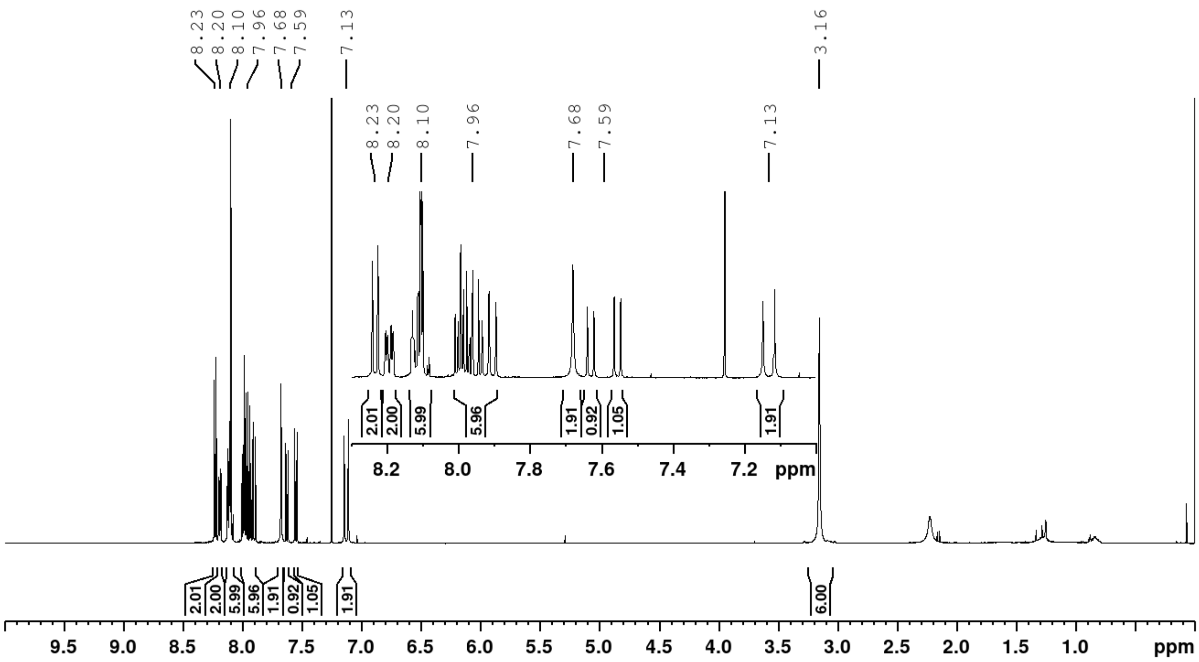

Figure S3. ^{1}H NMR spectrum of the ligand **Pyr1** in $CDCl_3$.

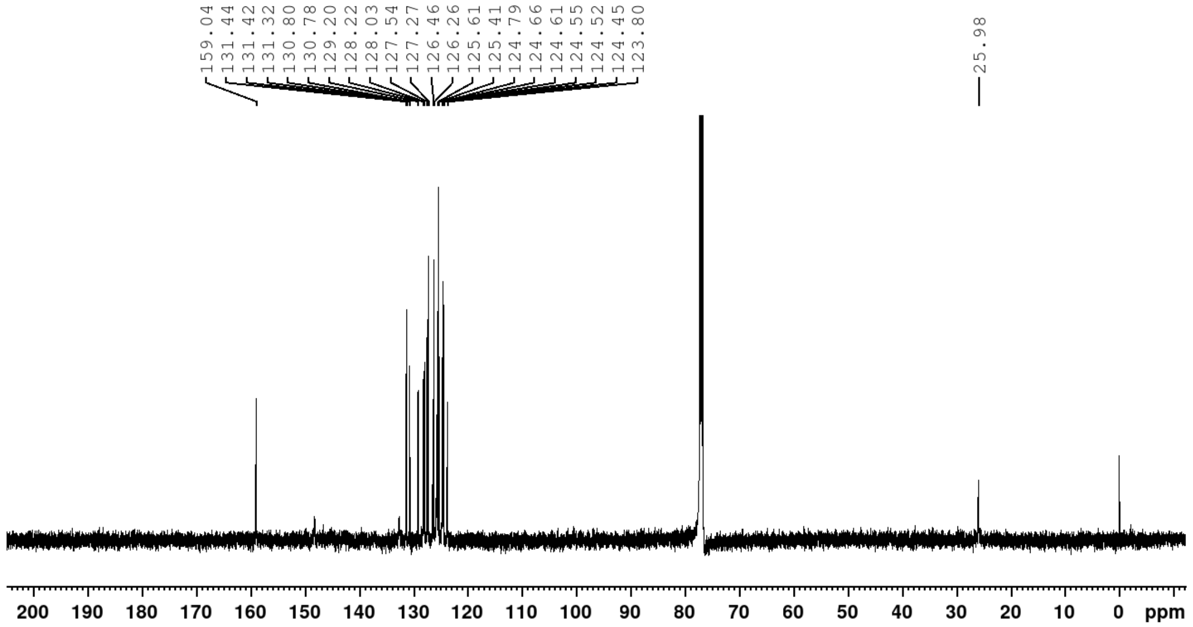

Figure S4. ^{13}C NMR spectrum of the ligand **Pyr1** in $CDCl_3$.

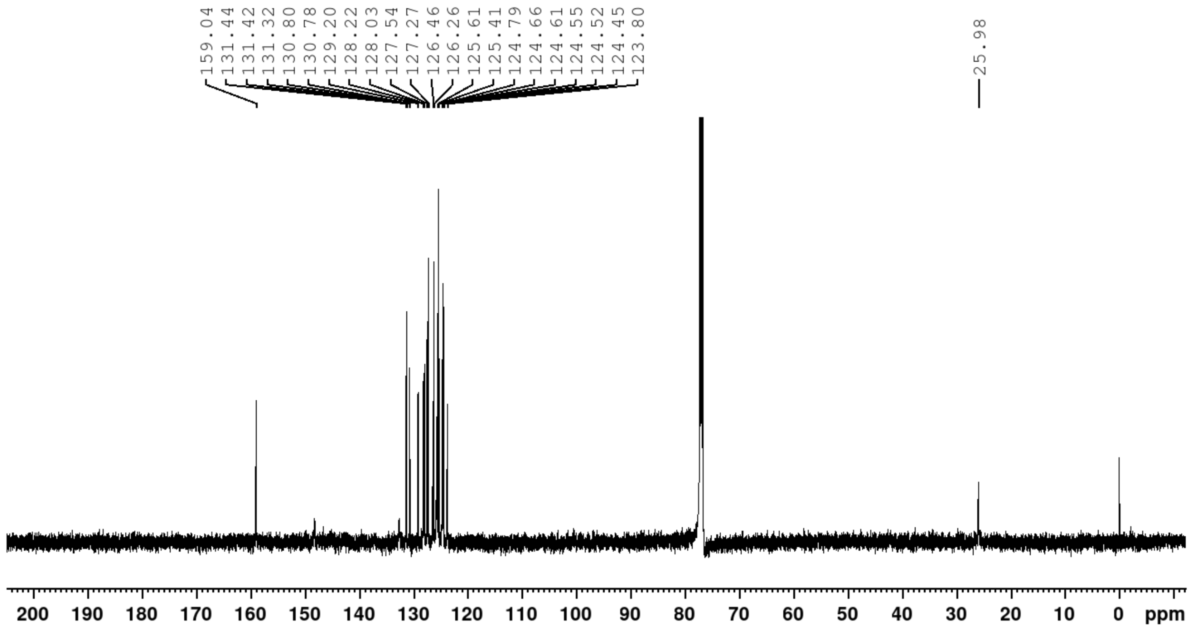

Figure S5. DEPT NMR spectrum of the ligand **Pyr1** in $CDCl_3$.

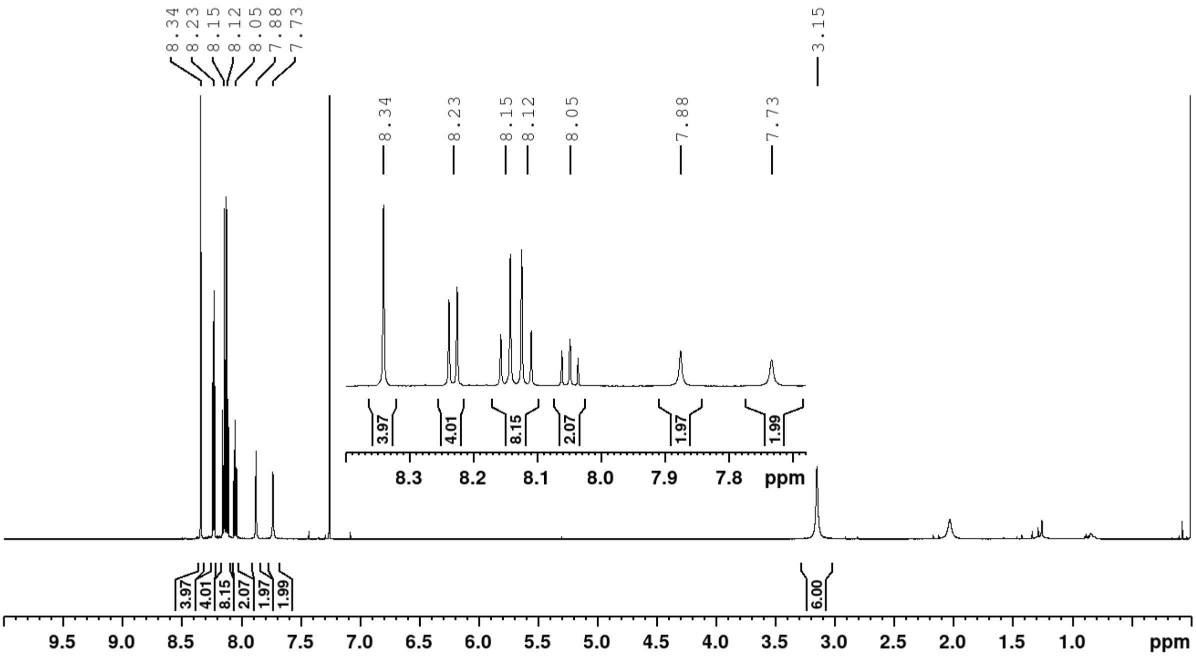

Figure S6. ^{1}H NMR spectrum of the ligand **Pyr2** in $CDCl_3$.

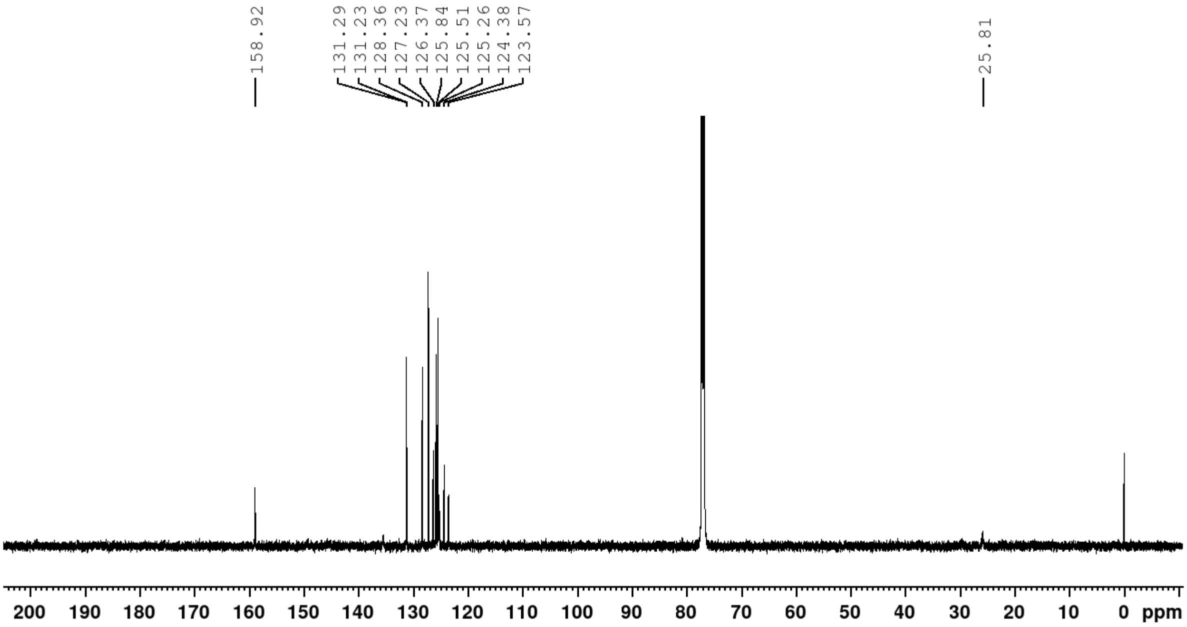

Figure S7. ^{13}C NMR spectrum of the ligand **Pyr2** in $CDCl_3$.

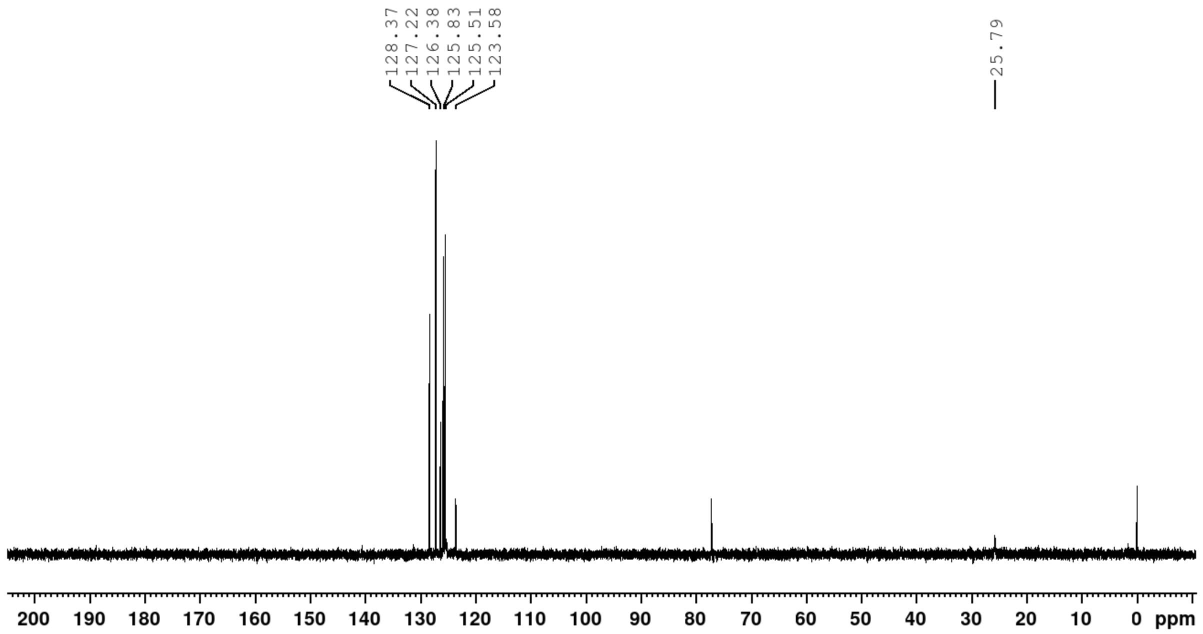

Figure S8. DEPT NMR spectrum of the ligand **Pyr2** in $CDCl_3$.

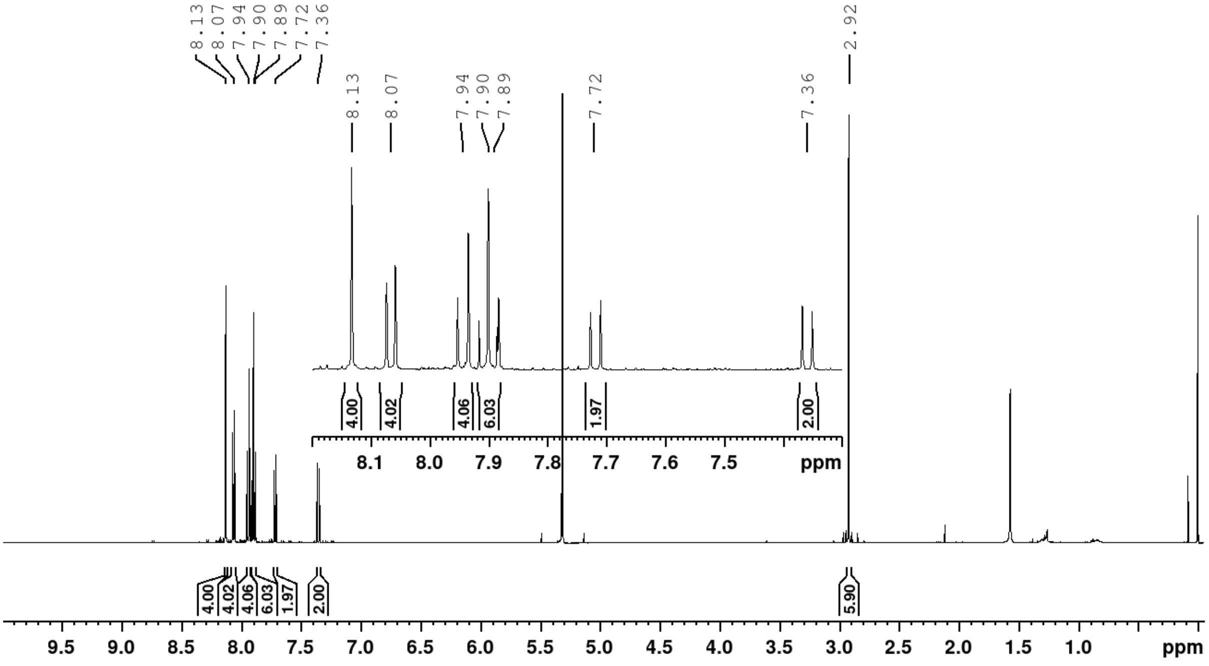

Figure S9. ^{1}H NMR spectrum of the ligand **Pyr3** in CD_2Cl_2 in $CDCl_3$

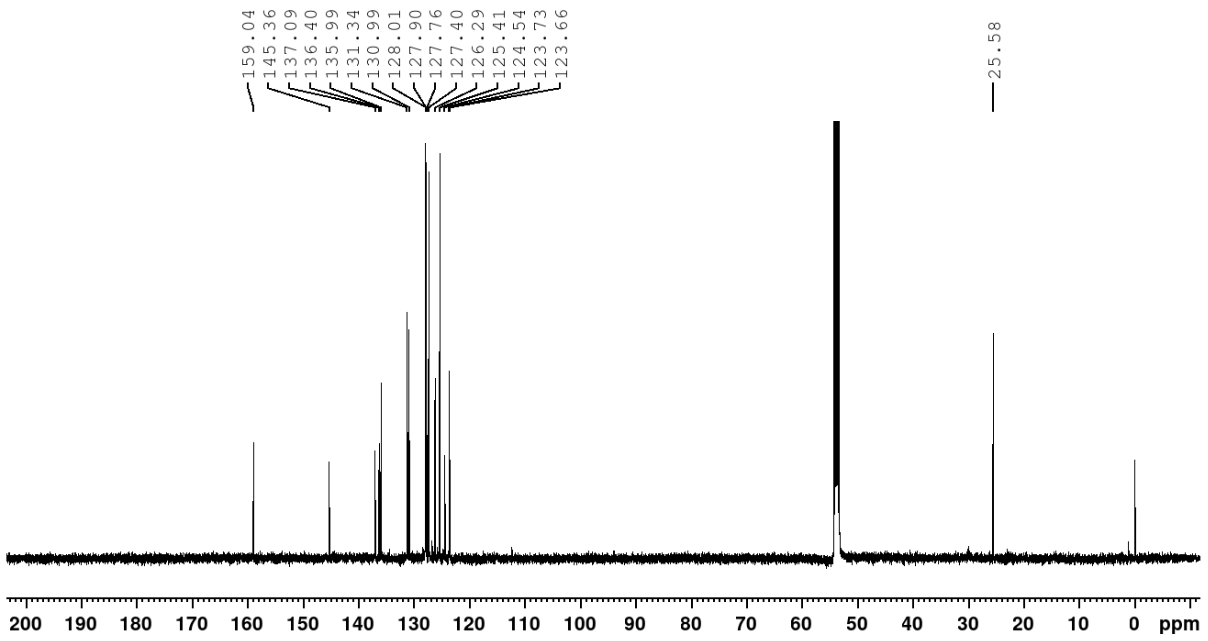

Figure S10. ^{13}C NMR spectrum of the ligand **Pyr3** in CD_2Cl_2.

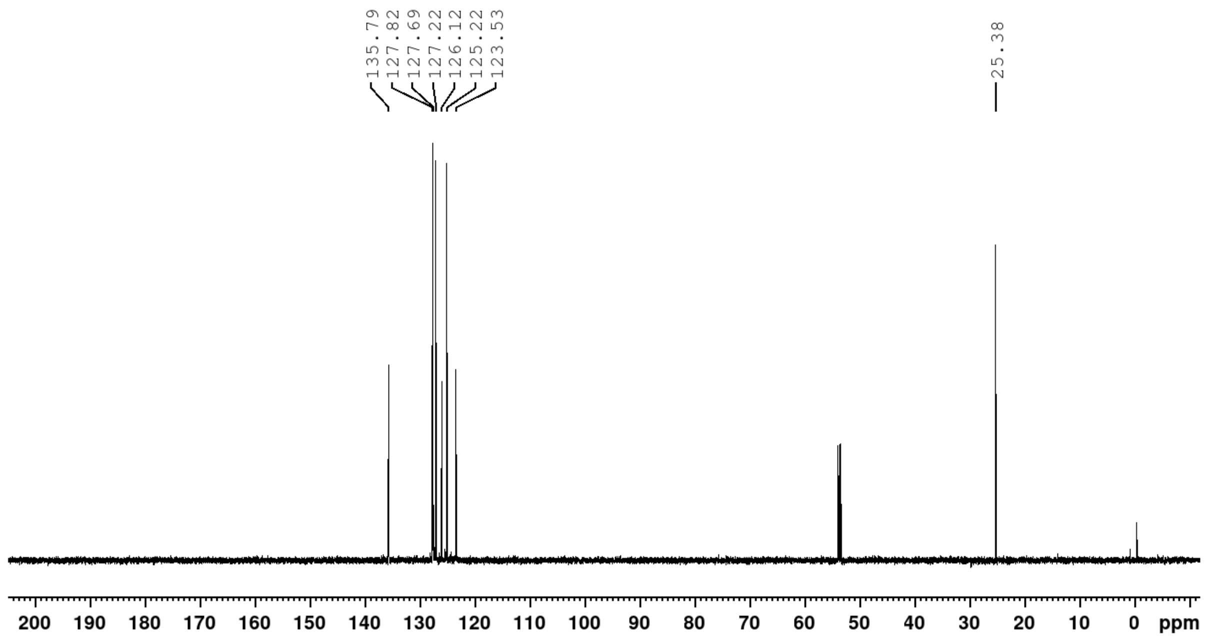

Figure S11. DEPT NMR spectrum of the ligand **Pyr3** in CD_2Cl_2.

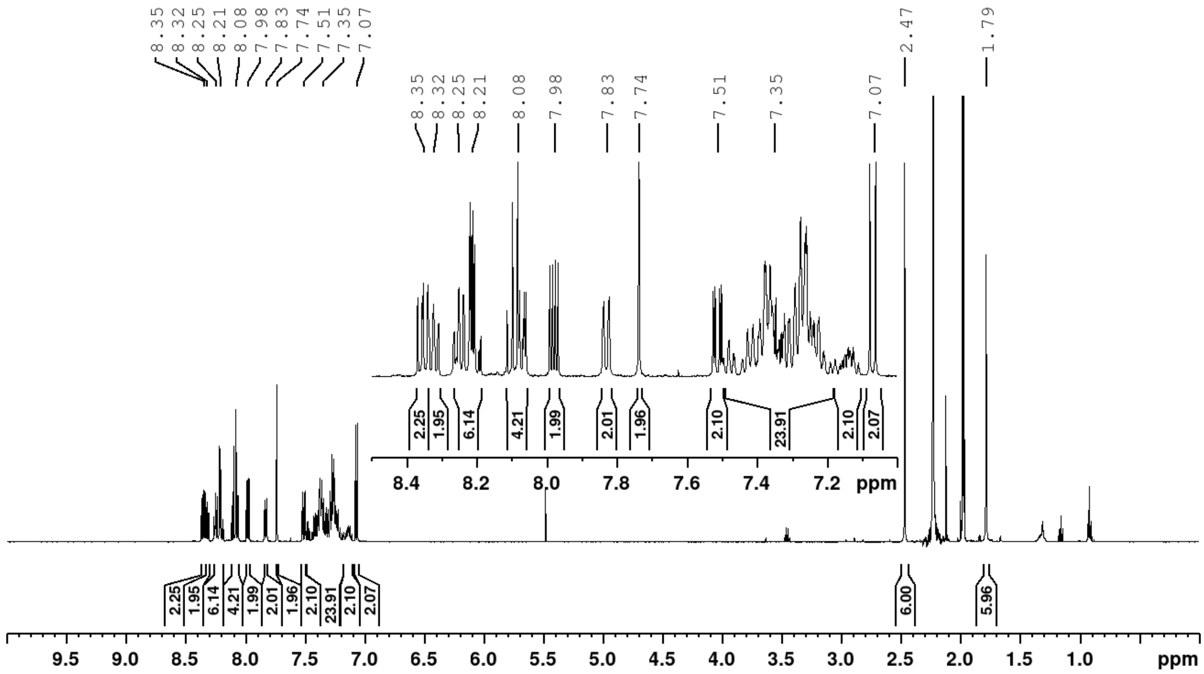

Figure S12. ^{1}H NMR spectrum of the complex [Cu(**Pyr1**)(xant)]PF_6 - **CuPyr1** in CD_3CN.

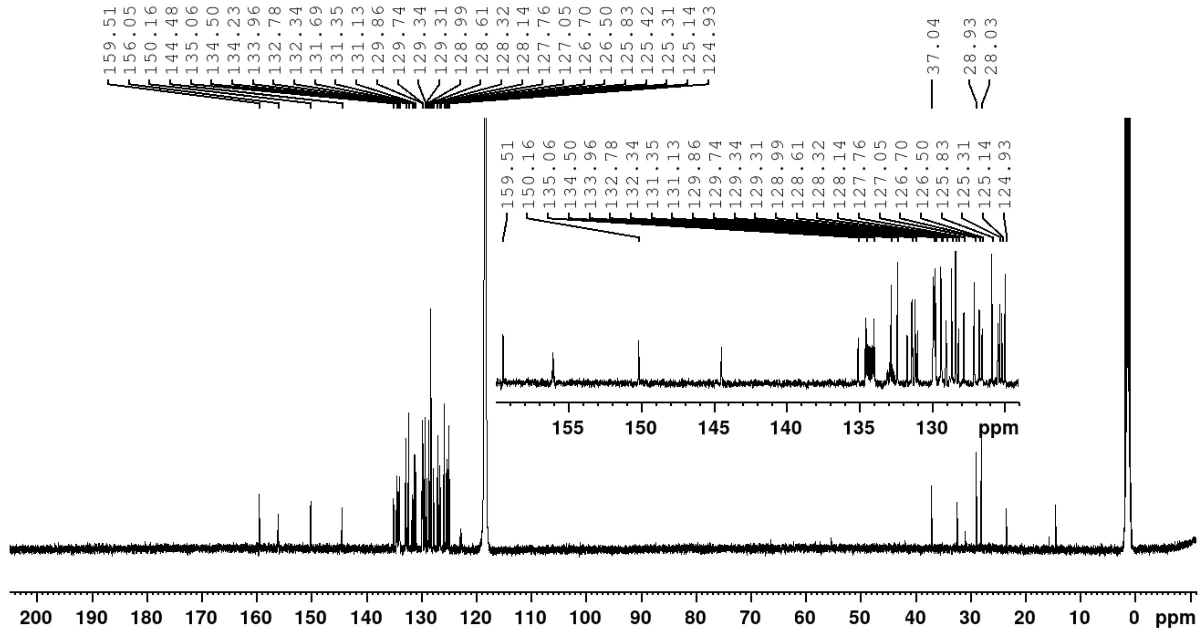

Figure S13. ^{13}C NMR spectrum of the complex [Cu(**Pyr1**)(xant)]PF_6 - **CuPyr1** in CD_3CN.

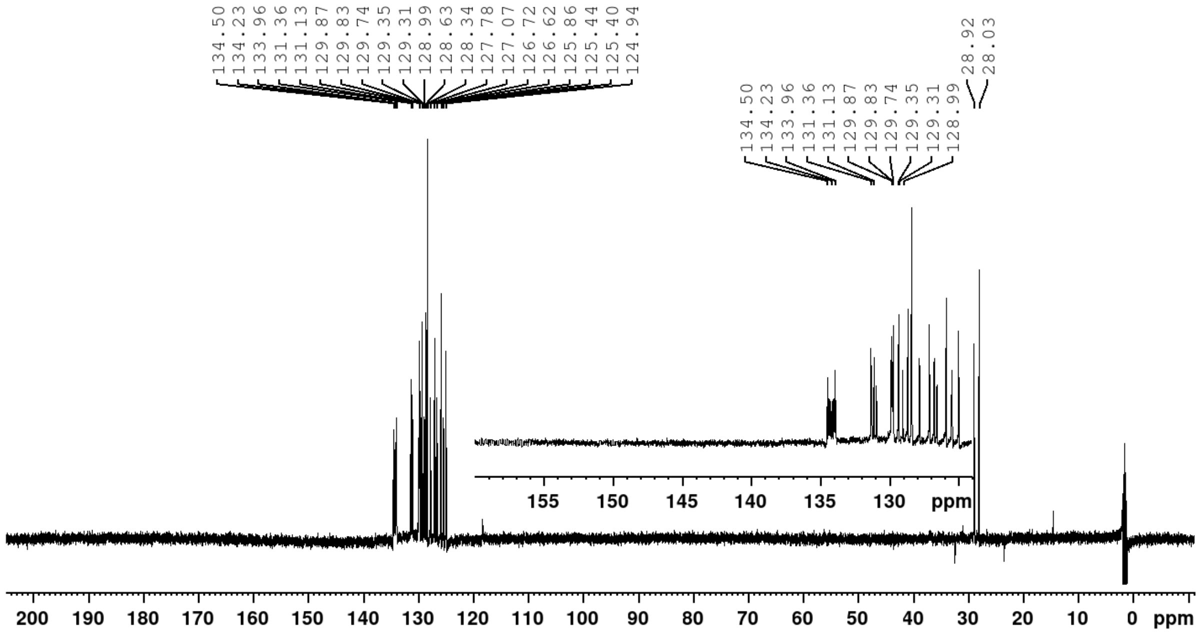

Figure S14. DEPT NMR spectrum of the complex [Cu(**Pyr1**)(xant)]PF_6 - **CuPyr1** in CD_3CN.

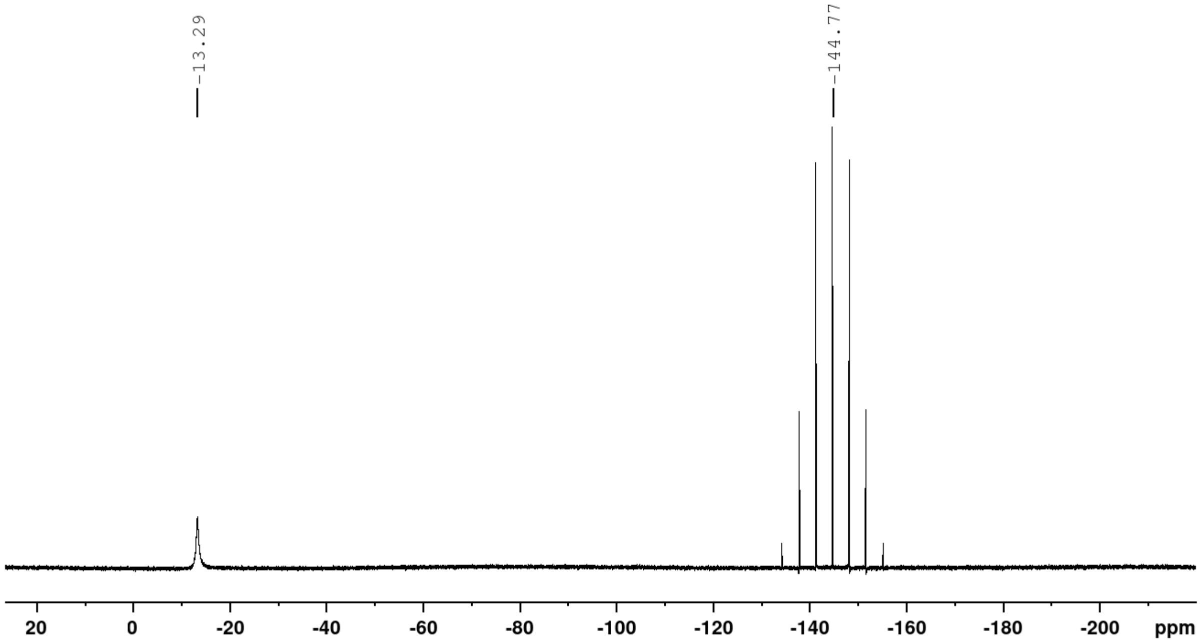

Figure S15. ^{31}P NMR spectrum of the complex [Cu(**Pyr1**)(xant)]PF_6 - **CuPyr1** in CD_3CN.

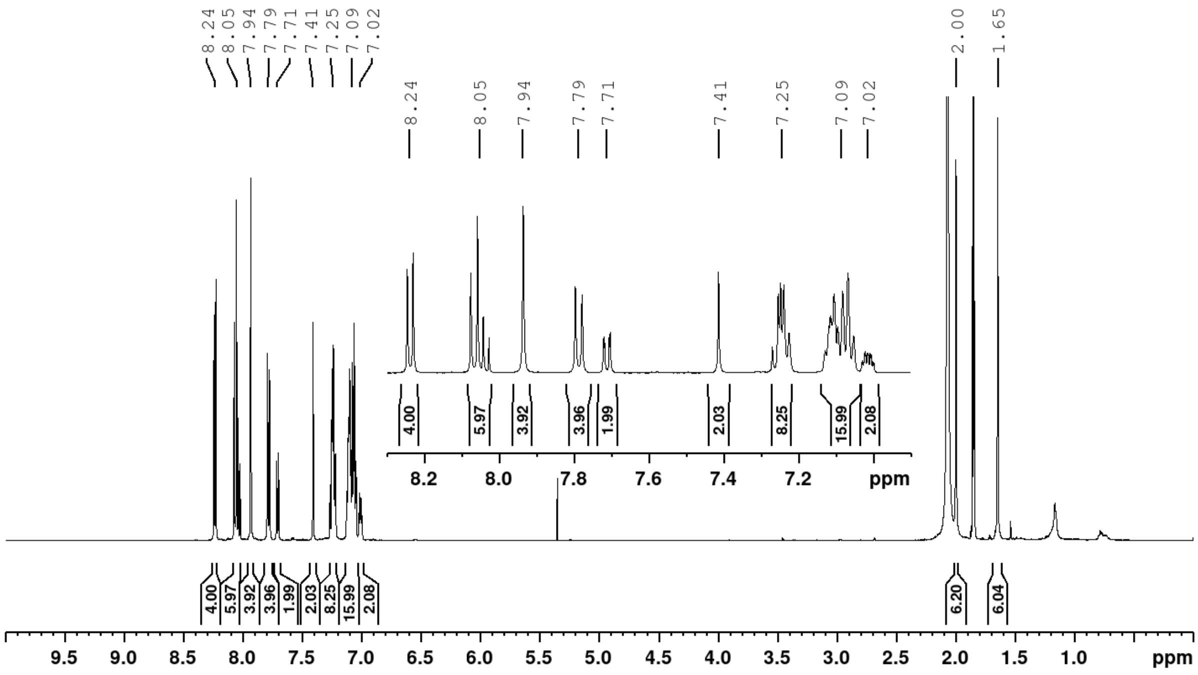

Figure S16. ^{1}H NMR spectrum of the complex $[Cu(\textbf{Pyr2})(xant)]PF_6$ - **CuPyr2** in CD_3CN.

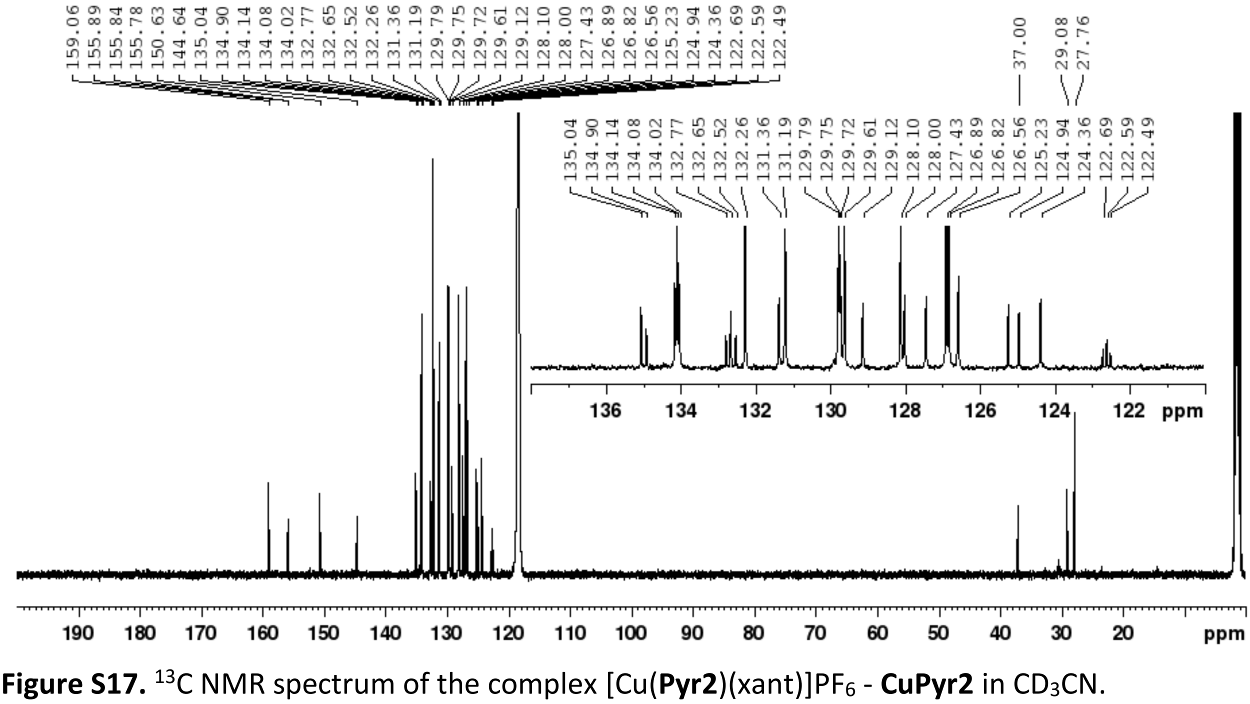

Figure S17. ^{13}C NMR spectrum of the complex $[Cu(\textbf{Pyr2})(xant)]PF_6$ - **CuPyr2** in CD_3CN.

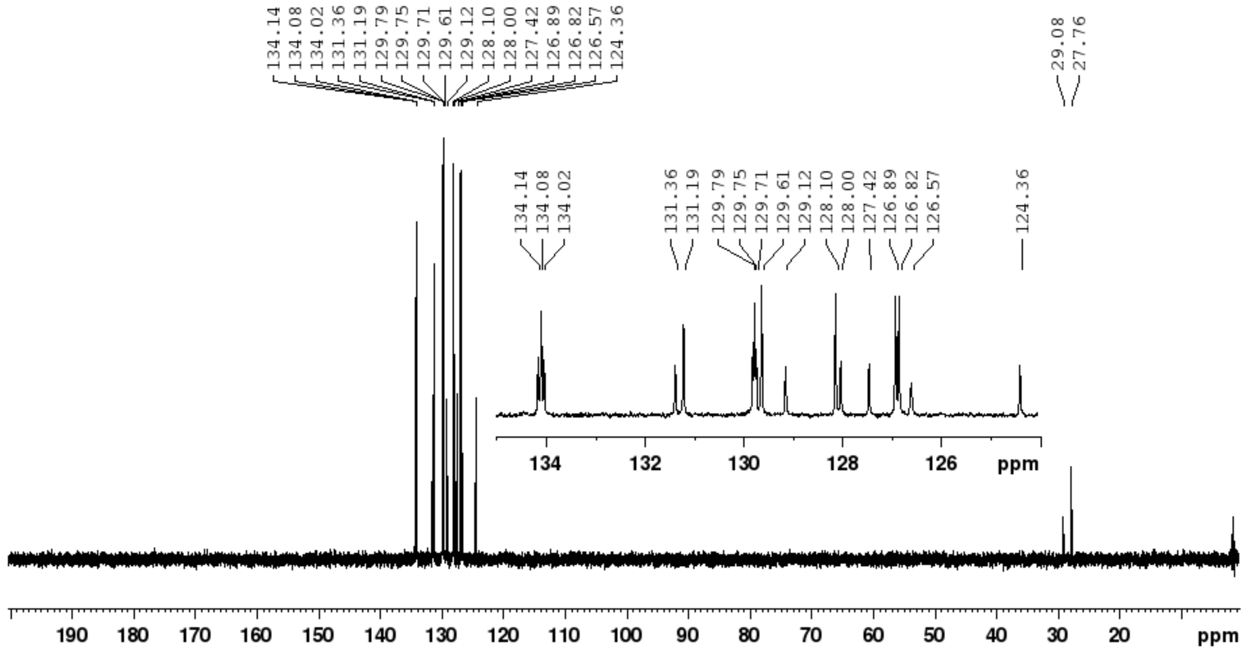

Figure S18. DEPT NMR spectrum of the complex [Cu(**Pyr2**)(xant)]PF_6 - **CuPyr2** in CD_3CN.

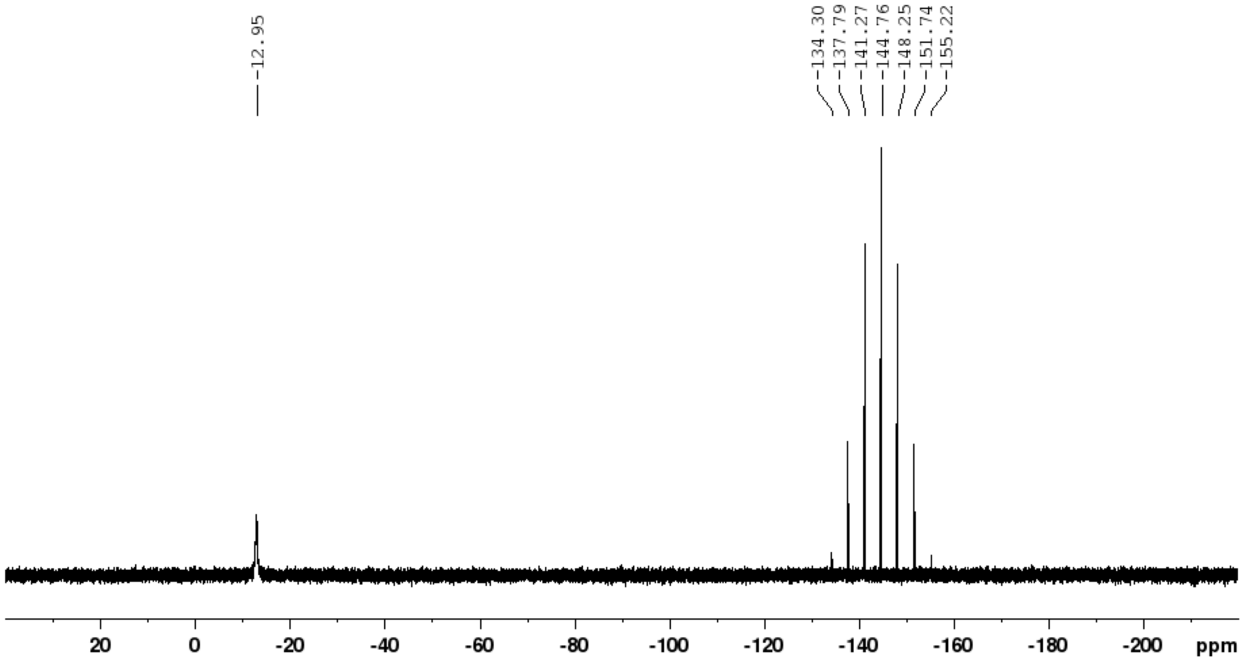

Figure S19. ^{31}P NMR spectrum of the complex [Cu(**Pyr2**)(xant)]PF_6 - **CuPyr2** in CD_3CN.

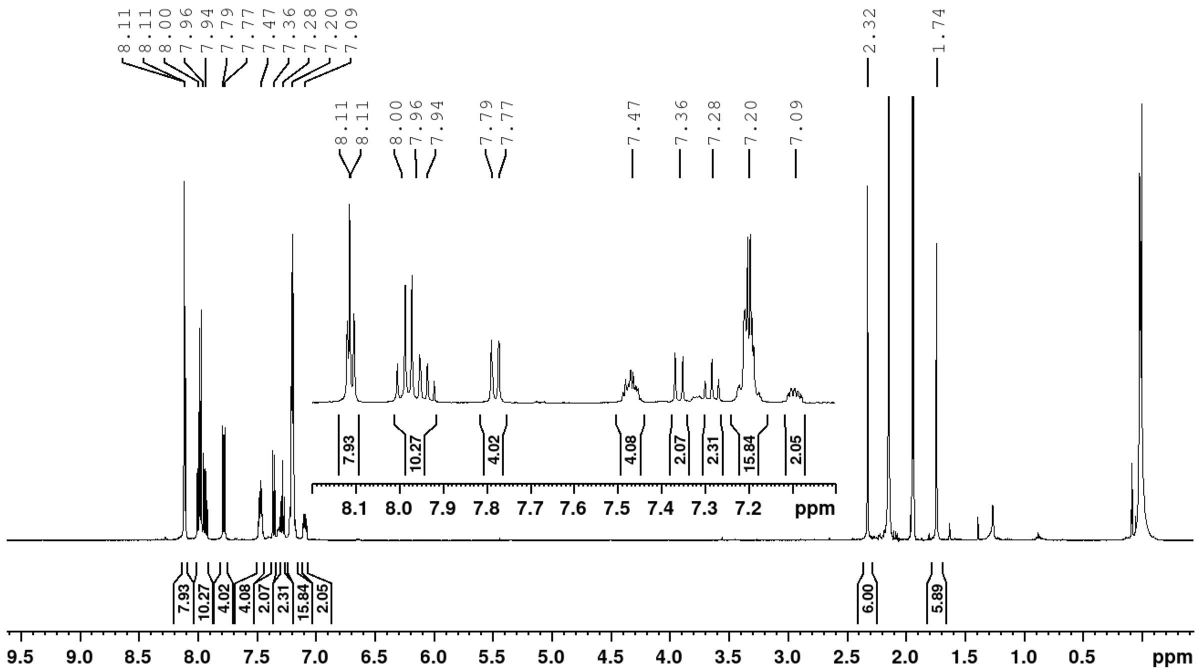

Figure S20. ^{1}H NMR spectrum of the complex [Cu(**Pyr3**)(xant)]PF_6 - **CuPyr3** in CD_3CN.

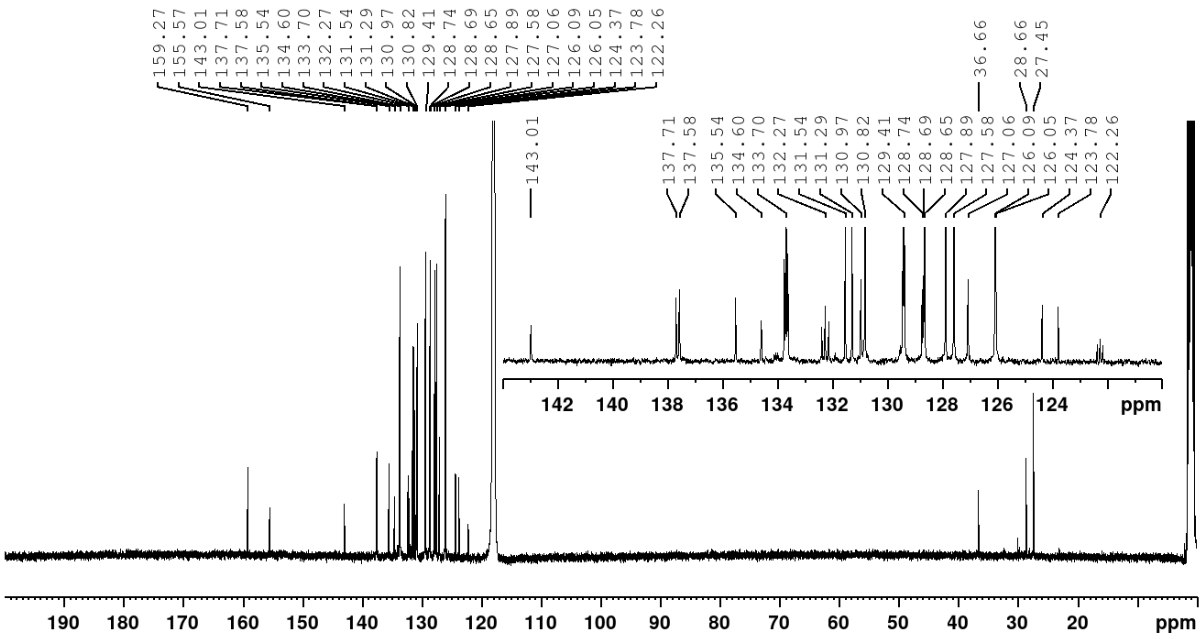

Figure S21. ^{13}C NMR spectrum of the complex [Cu(**Pyr3**)(xant)]PF_6 - **CuPyr3** in CD_3CN.

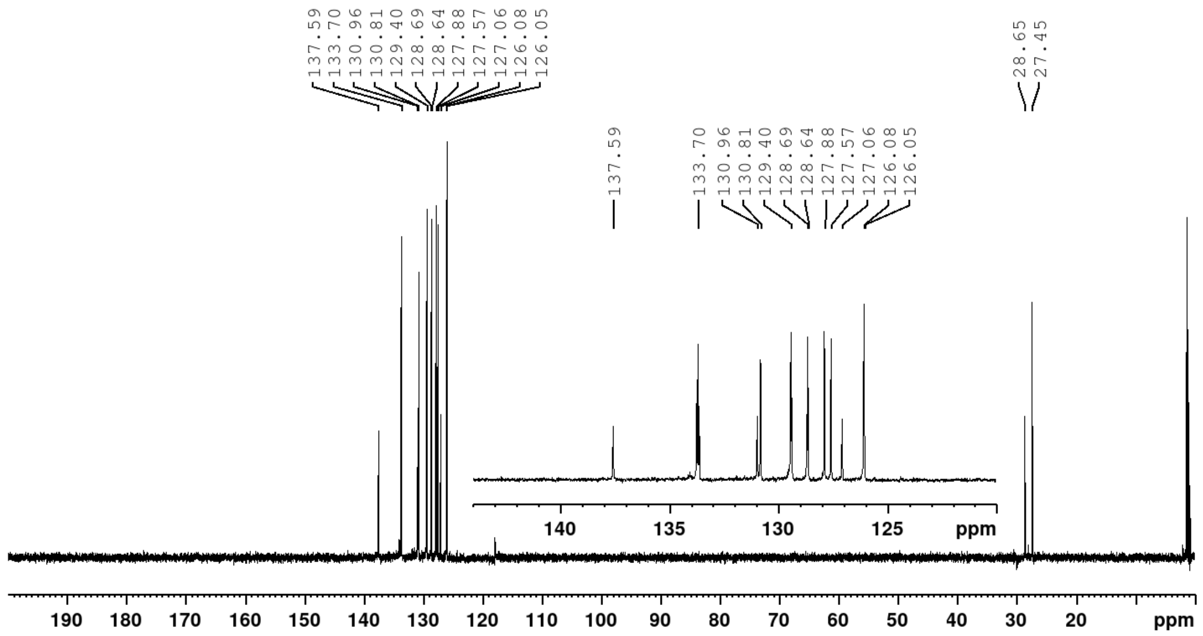

Figure S22. DEPT NMR spectrum of the complex [Cu(**Pyr3**)(xant)]PF_6 - **CuPyr3** in CD_3CN.

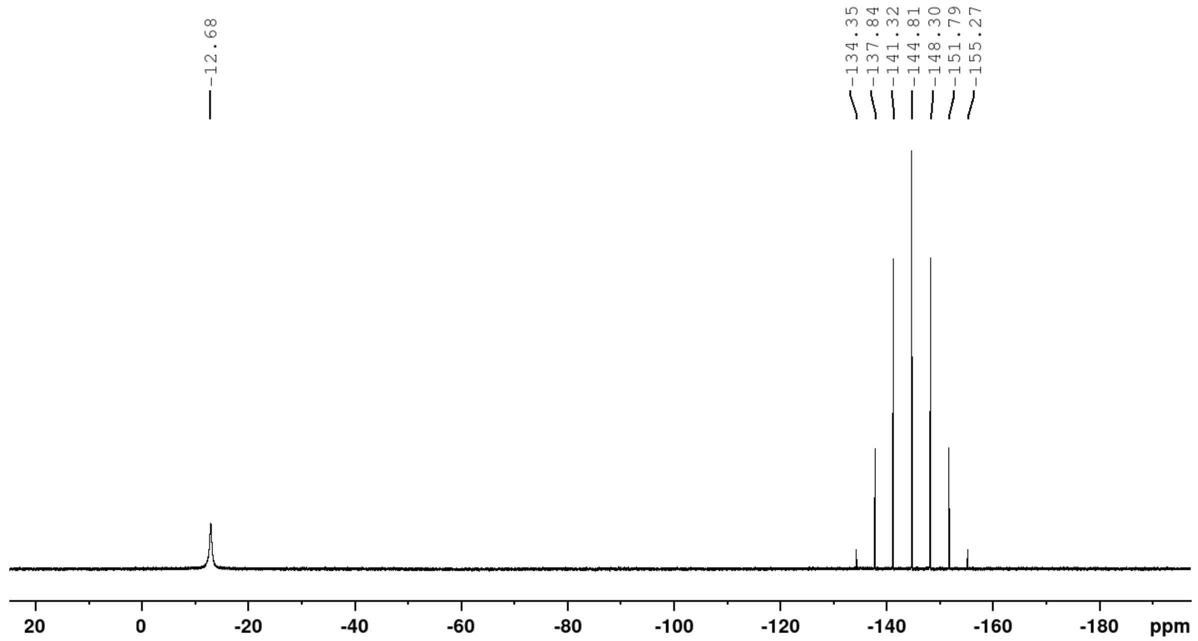

Figure S23. ^{31}P NMR spectrum of the complex [Cu(**Pyr3**)(xant)]PF_6 - **CuPyr3** in CD_3CN.

4 MS spectra

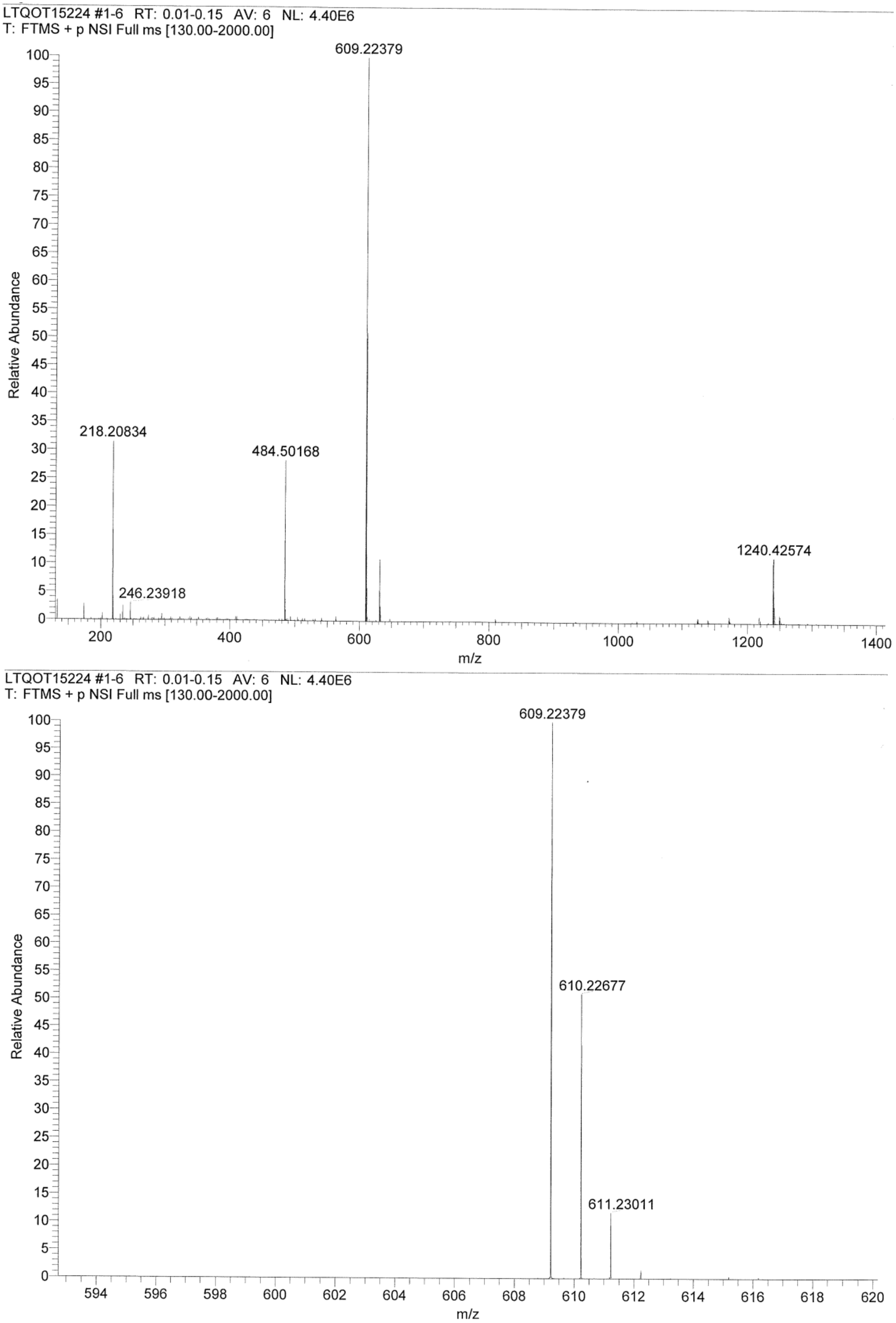

Figure S24. High resolution ESI mass (HRMS) spectrum of **Pyr1**.

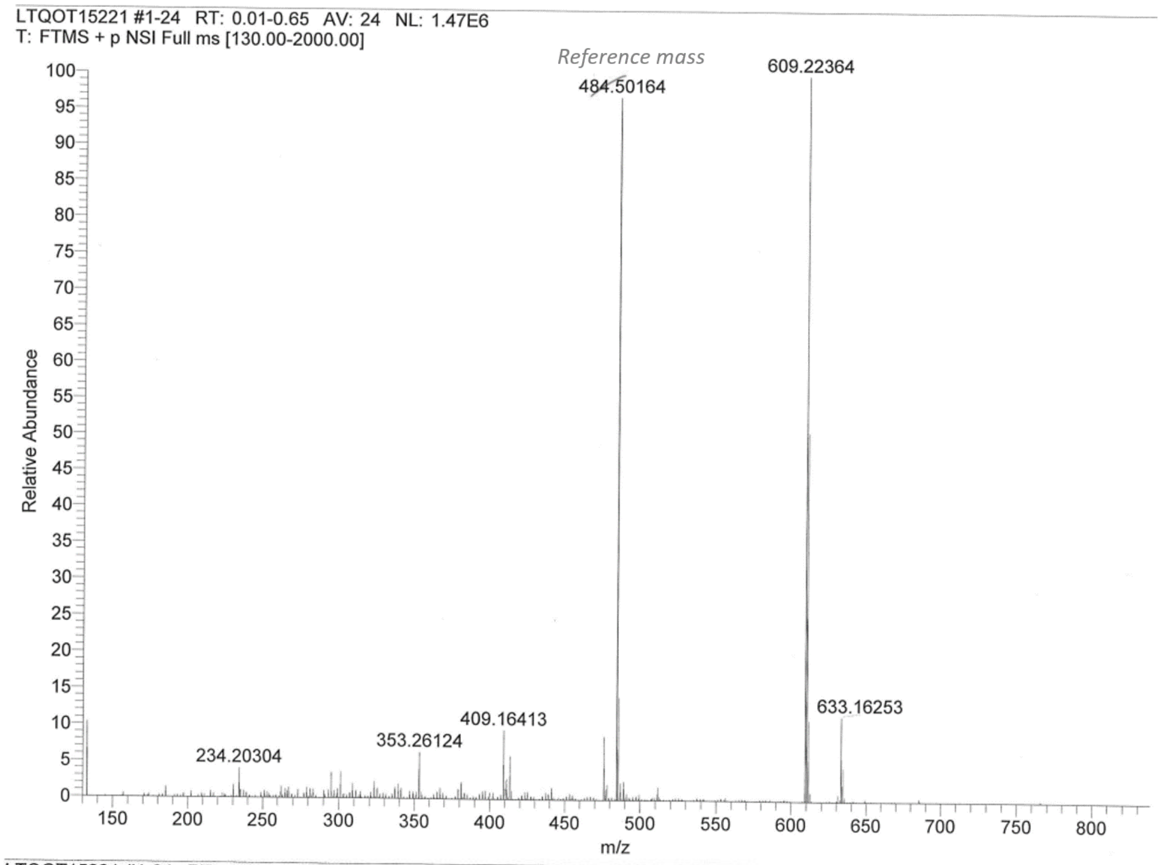

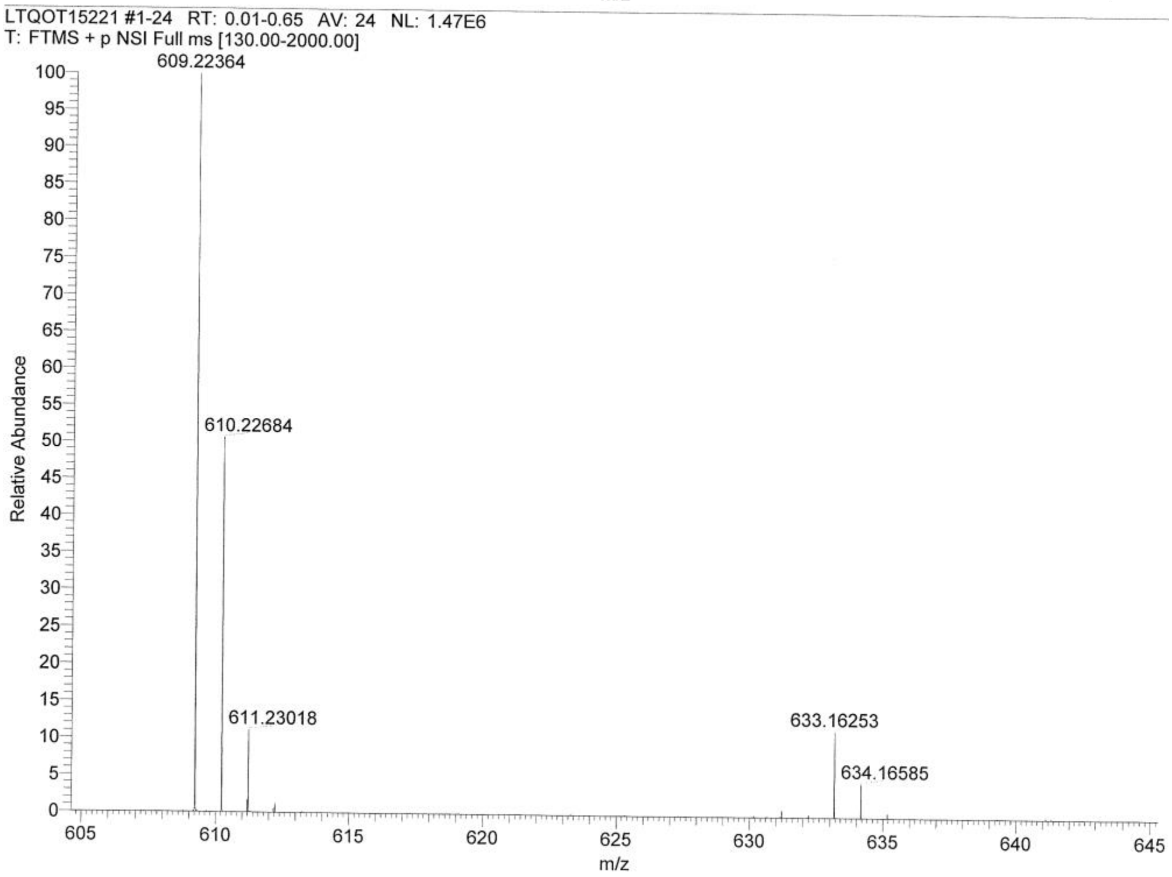

Figure S25. High resolution ESI mass (HRMS) spectrum of **Pyr2**.

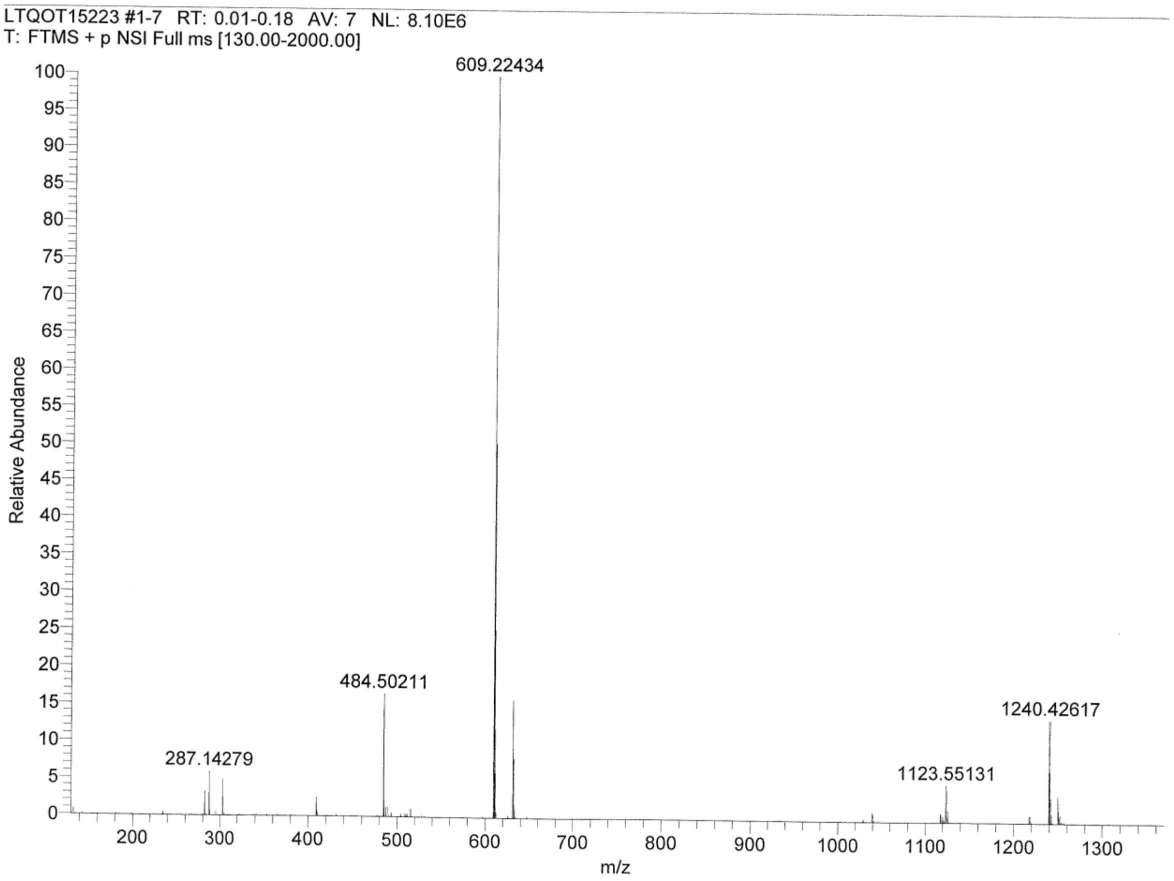

Figure S26. High resolution ESI mass (HRMS) spectrum of **Pyr3**.

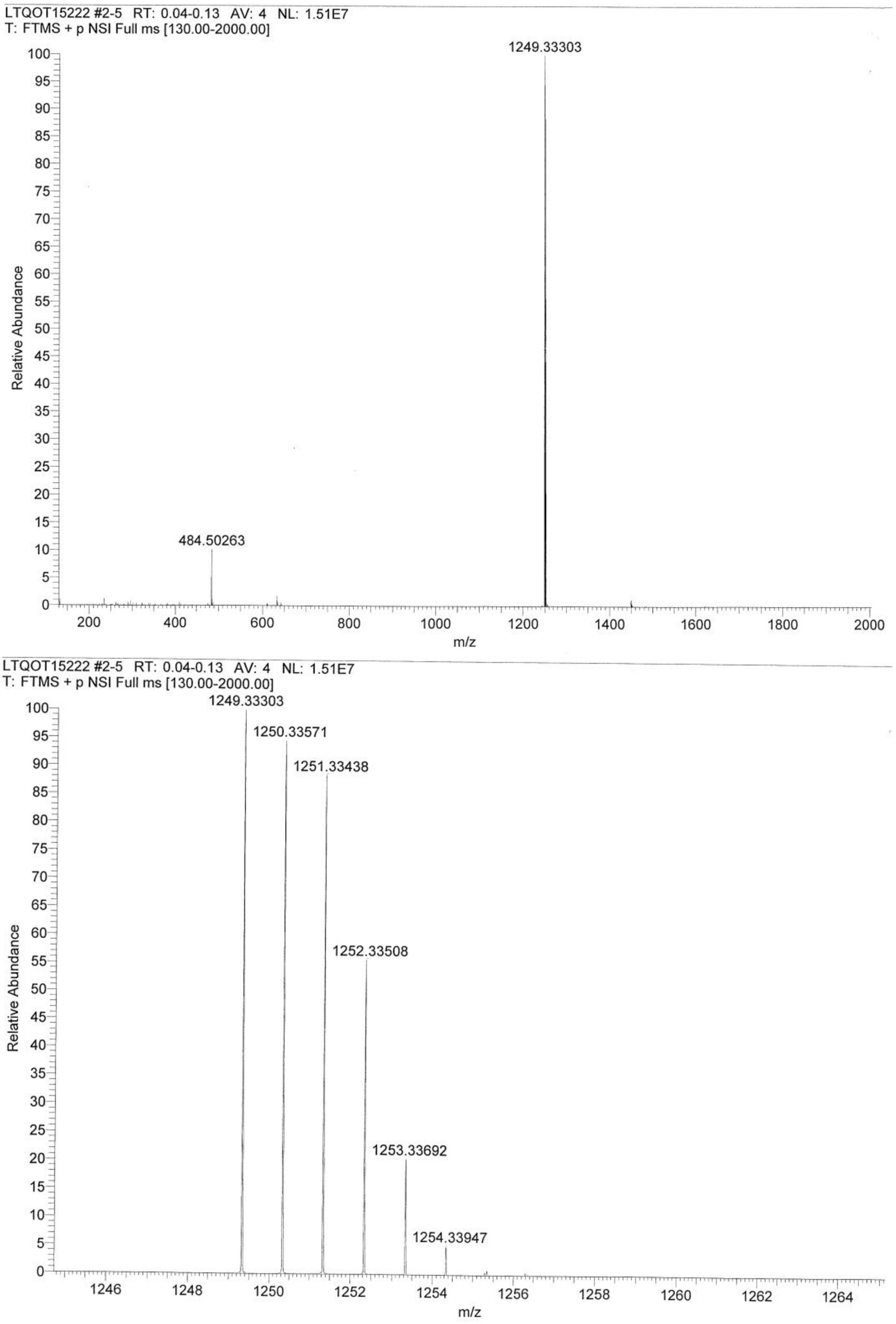

Figure S27. High resolution ESI mass (HRMS) spectrum of [Cu(**Pyr1**)(xant)]PF_6 - **CuPyr1**.

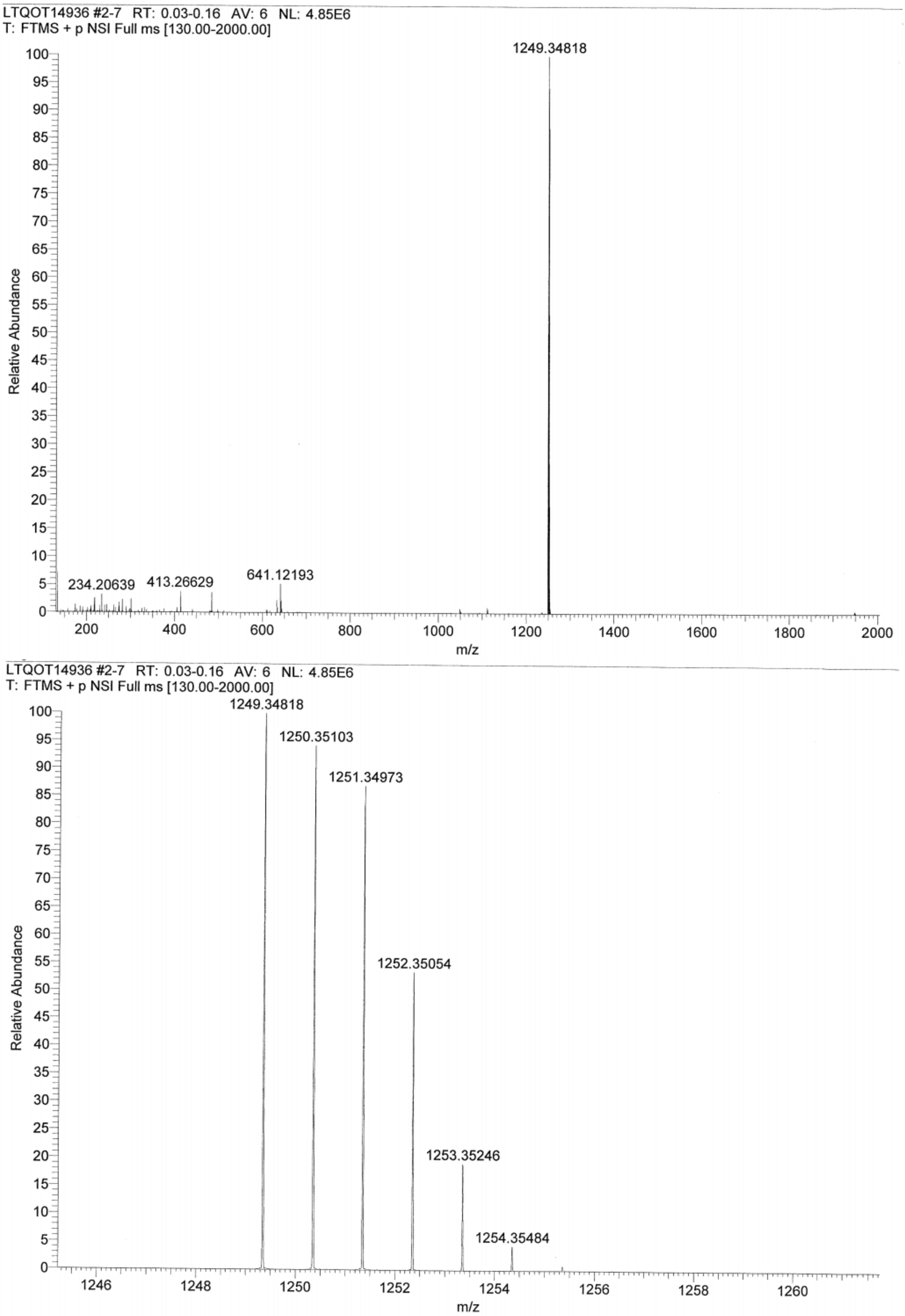

Figure S28. High resolution ESI mass (HRMS) spectrum of [Cu(**Pyr2**)(xant)]PF_6 - **CuPyr2**.

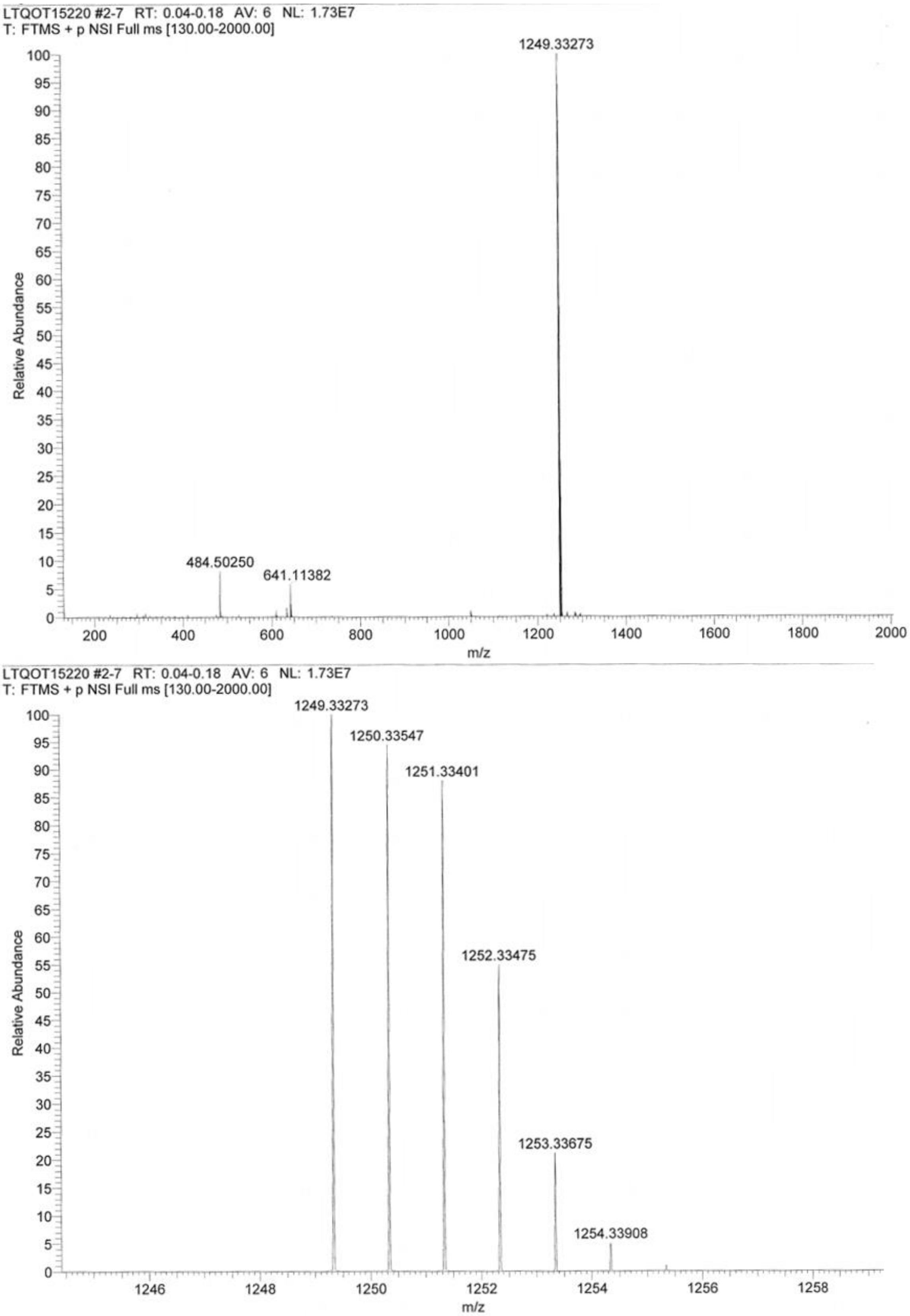

Figure S29. High resolution ESI mass (HRMS) spectrum of [Cu(**Pyr3**)(xant)]PF_6 - **CuPyr3**.

5 Electrochemical Data

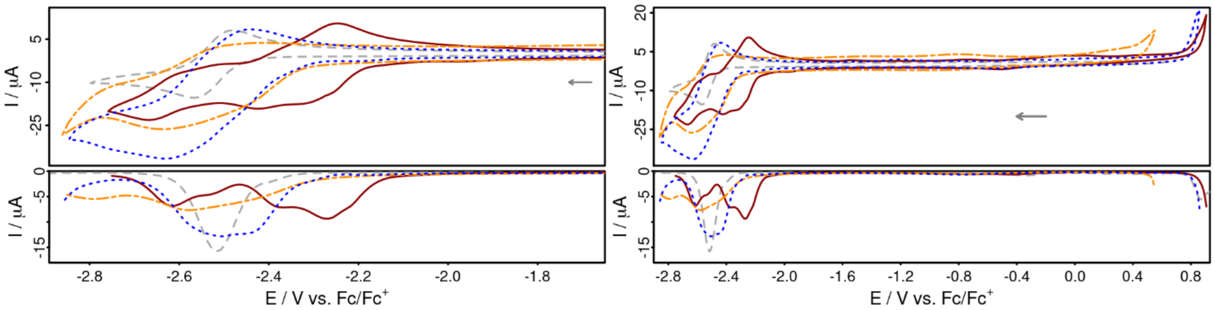

Figure S30. Cyclic voltammograms (top) and differential pulse voltammograms (bottom) of **Pyrene** (grey, dashed, 1 mM), **Pyr1** (red, solid, 1 mM), **Pyr2** (orange, double dash, 1 mM), and **Pyr3** (blue, dotted, 1 mM) in dimethylformamide solution referenced *vs.* the ferrocene/ferricenium (Fc/Fc^+) couple. Conditions: scan rate of 100 mVs^{-1}, $[Bu_4N][PF_6]$ (0.1 M) as supporting electrolyte.

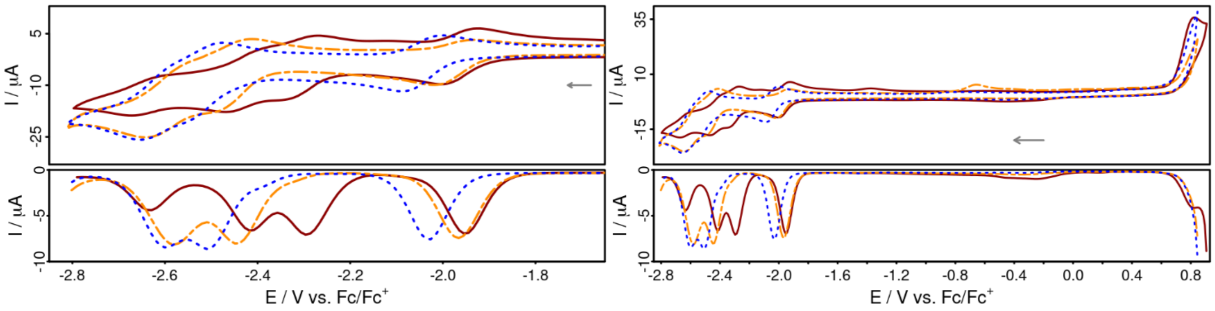

Figure S31. Cyclic voltammograms (top) and differential pulse voltammograms (bottom) of **CuPyr1** (red, solid, 1 mM), **CuPyr2** (orange, double dash, 1 mM), and **CuPyr3** (blue, dotted, 1 mM) in dimethylformamide solution referenced *vs.* the ferrocene/ferricenium (Fc/Fc^+) couple. Conditions: scan rate of 100 mVs^{-1}, $[Bu_4N][PF_6]$ (0.1 M) as supporting electrolyte.

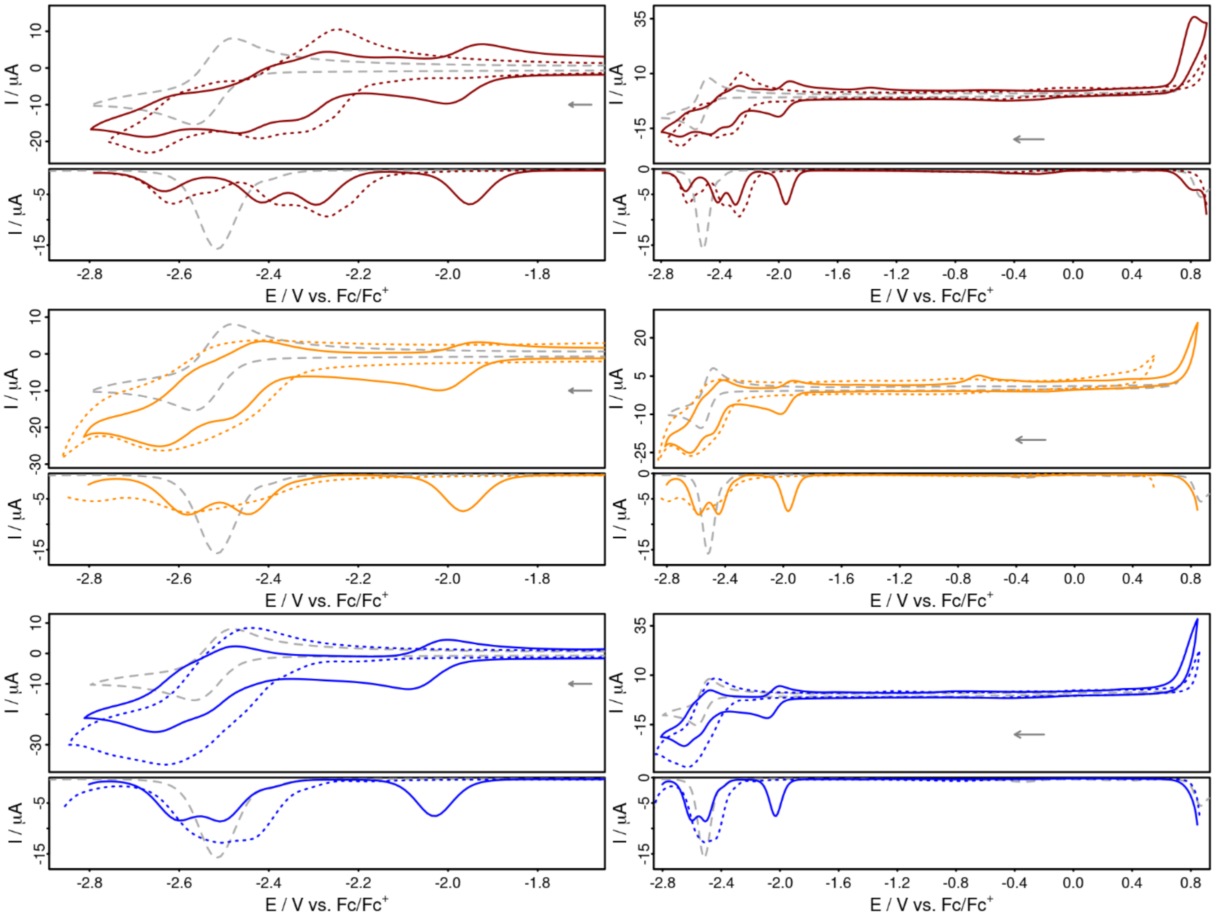

Figure S32. Cyclic voltammograms and differential pulse voltammograms of
Top: **Pyr1** (dark red, dotted) and **CuPyr1** (dark red, solid),
Middle: **Pyr2** (orange, dotted) and **CuPyr2** (orange solid) and
Bottom: **Pyr3** (blue, dotted) and **CuPyr3** (blue, solid).
In dimethylformamide solution referenced *vs.* the ferrocene/ferricenium (Fc/Fc^{+}) couple. Pyrene (grey, dashed) is always depicted as reference. Conditions: concentration of the compound of 1 mM, rate of 100 mVs^{-1}, $[Bu_4N][PF_6]$ (0.1 M) as supporting electrolyte.

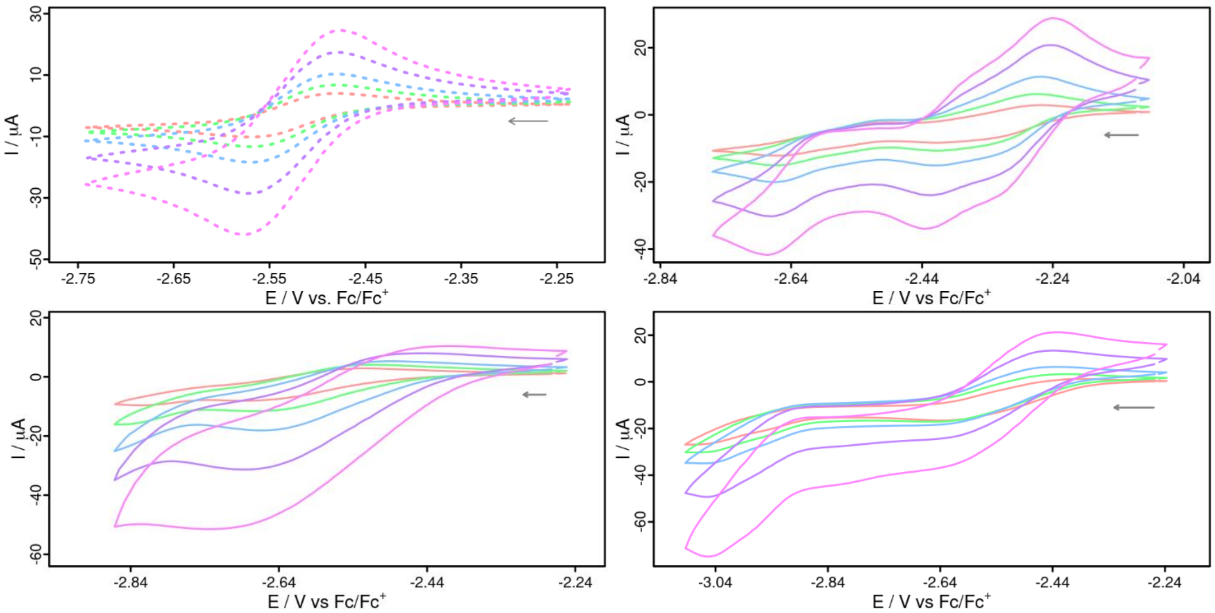

Figure S33. Reductive events of the cyclic voltammograms of pyrene (top left), **Pyr1** (top, right), **Pyr2** (bottom, left) and **Pyr3** (bottom, right) in dimethylformamide solution referenced *vs.* the ferrocene/ferricenium (Fc/Fc^+) couple at different scan rates. Conditions: scan rate of 25 mVs^{-1} (light red), 50 mVs^{-1} (light green), 100 mVs^{-1} (light blue), 250 mVs^{-1} (light purple) and 500 mVs^{-1} (light magenta), with $[Bu_4N][PF_6]$ (0.1 M) as supporting electrolyte. The arrow illustrates the initial scan direction.

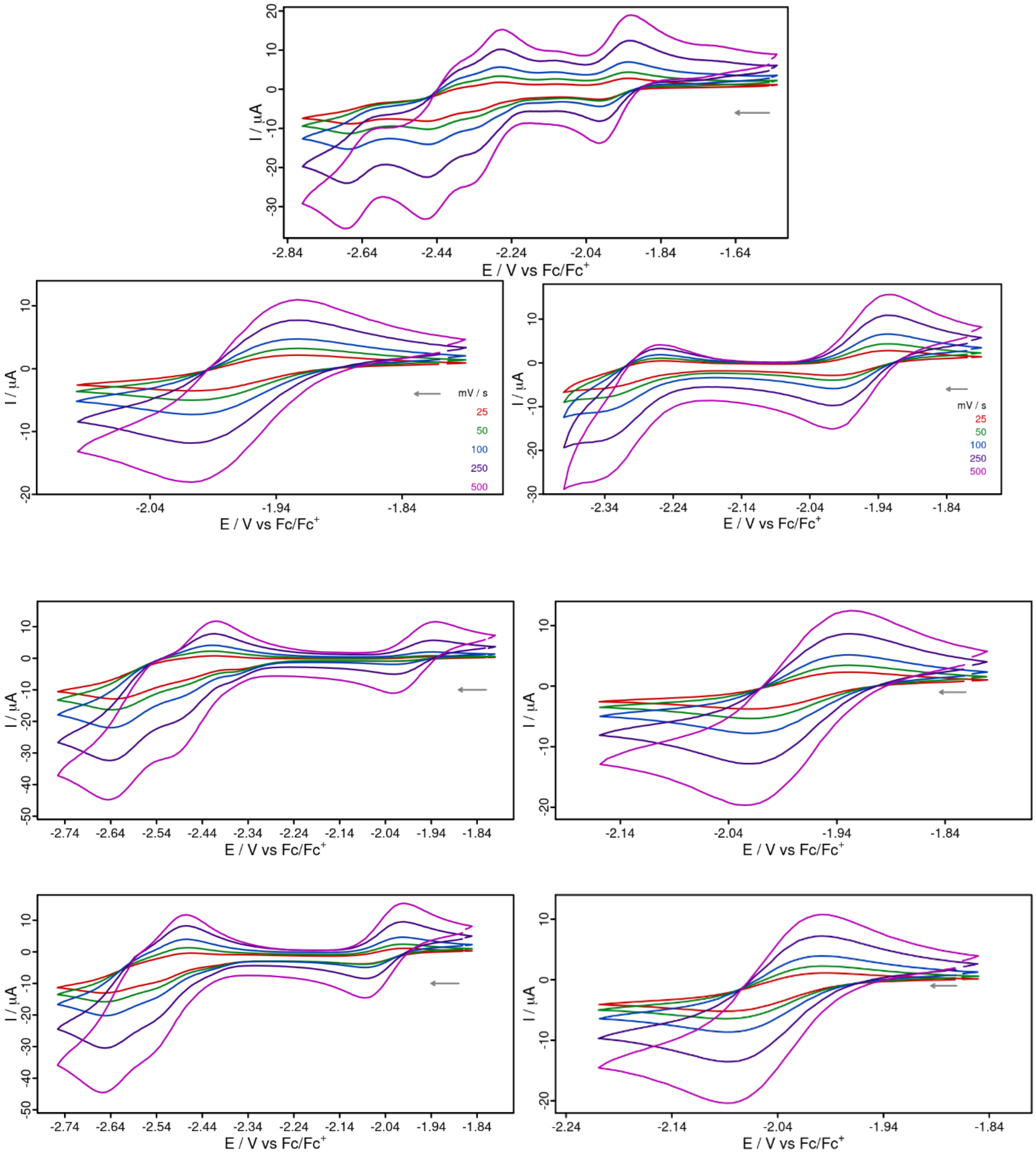

Figure S34. Reductive events of the cyclic voltammograms of **CuPyr1** (top and second top), **CuPyr2** (third from top) and **CuPyr3** (bottom) in dimethylformamide solution referenced *vs.* the ferrocene/ferricenium (Fc/Fc$^+$) couple at different scan rates. Conditions: scan rate of 25 mVs^{-1} (red), 50 mVs^{-1} (green), 100 mVs^{-1} (blue), 250 mVs^{-1} (purple) and 500 mVs^{-1} (magenta), with $[Bu_4N][PF_6]$ (0.1 M) as supporting electrolyte. The arrow illustrates the initial scan direction.

6 (Time-dependent) Density Functional Theory

6.1 Predicted Ground State Structures and Orbital Representation

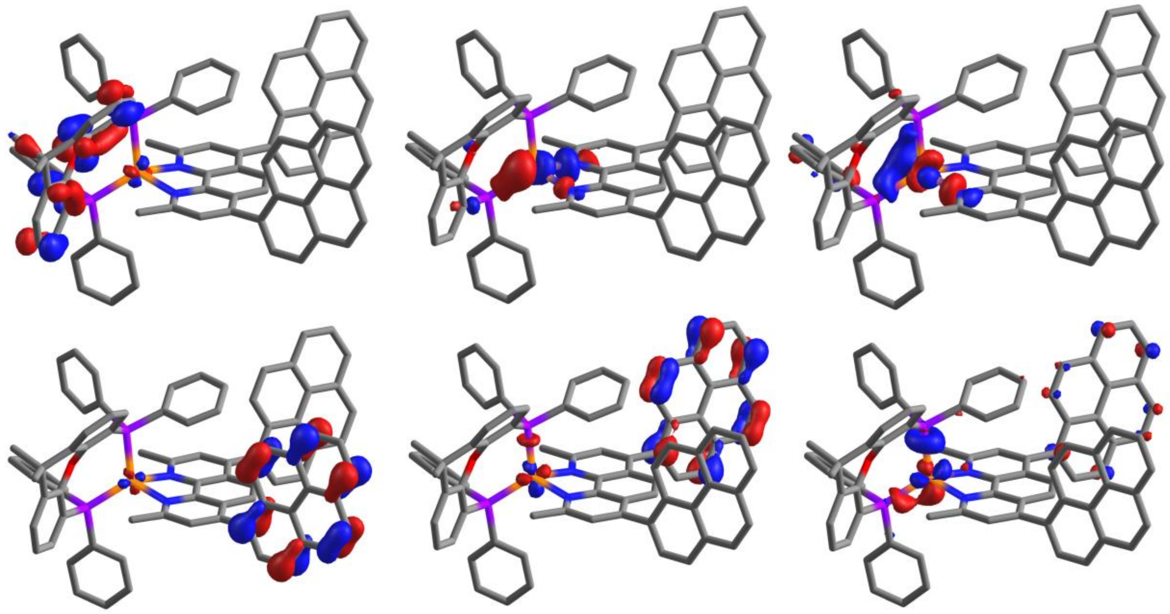

Figure S35. Orbital representation of **CuPyr1** showing the HOMO-5, HOMO-4, HOMO-3, HOMO-2, HOMO-1 and HOMO (from top left to bottom right).

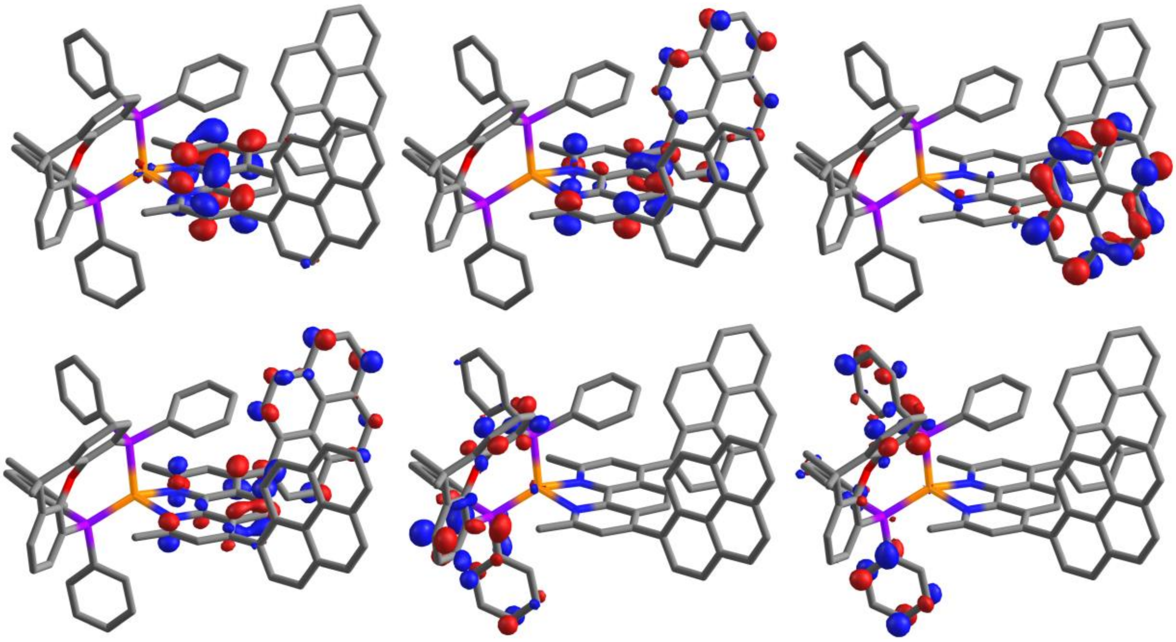

Figure S36. Orbital representation of **CuPyr1** showing the LUMO, LUMO+1, LUMO+2, LUMO+3, LUMO+4 and LUMO+5 (from top left to bottom right).

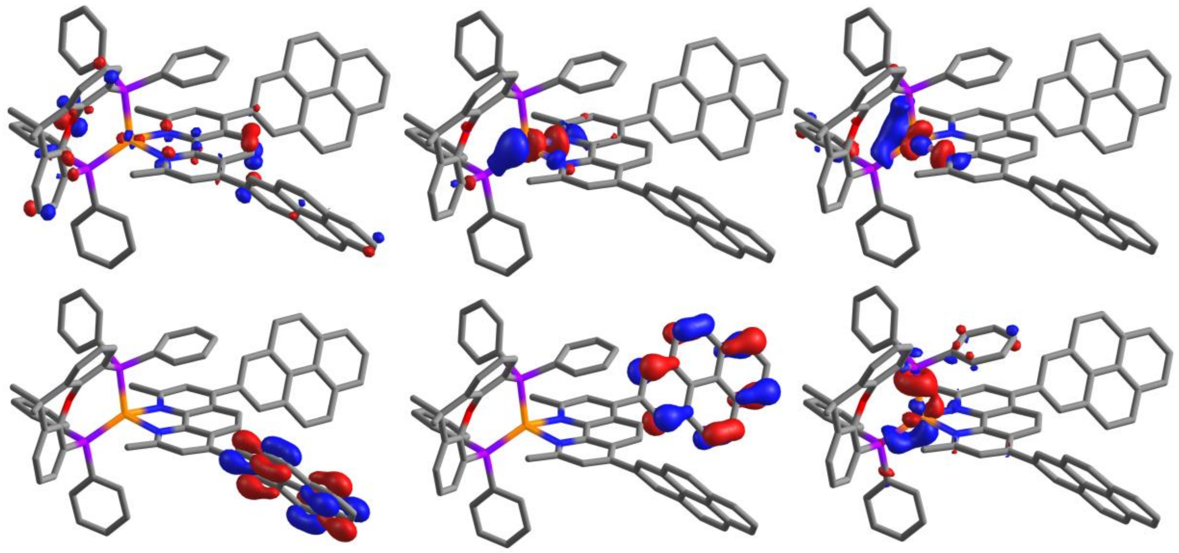

Figure S37. Orbital representation of **CuPyr2** showing the HOMO-5, HOMO-4, HOMO-3, HOMO-2, HOMO-1 and HOMO (from top left to bottom right).

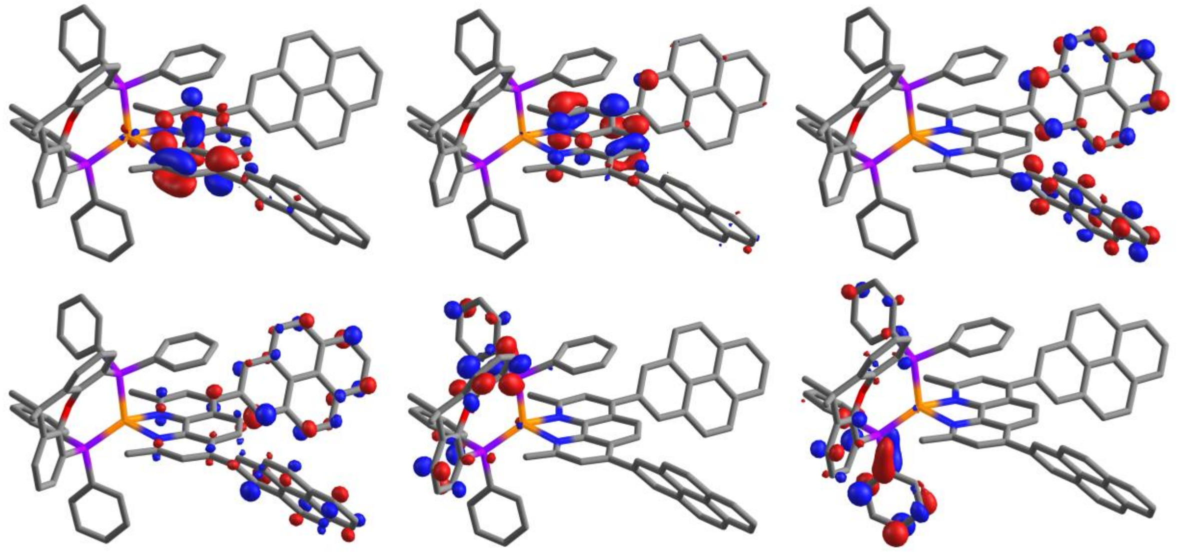

Figure S38. Orbital representation of **CuPyr2** showing the LUMO, LUMO+1, LUMO+2, LUMO+3, LUMO+4 and LUMO+5 (from top left to bottom right).

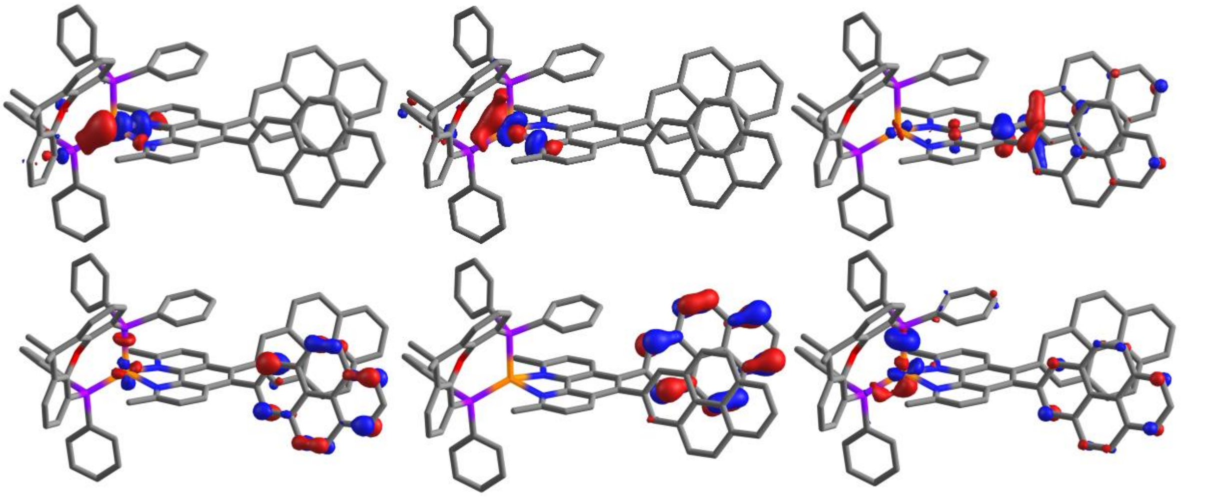

Figure S39. Orbital representation of **CuPyr3** showing the HOMO-5, HOMO-4, HOMO-3, HOMO-2, HOMO-1 and HOMO (from top left to bottom right).

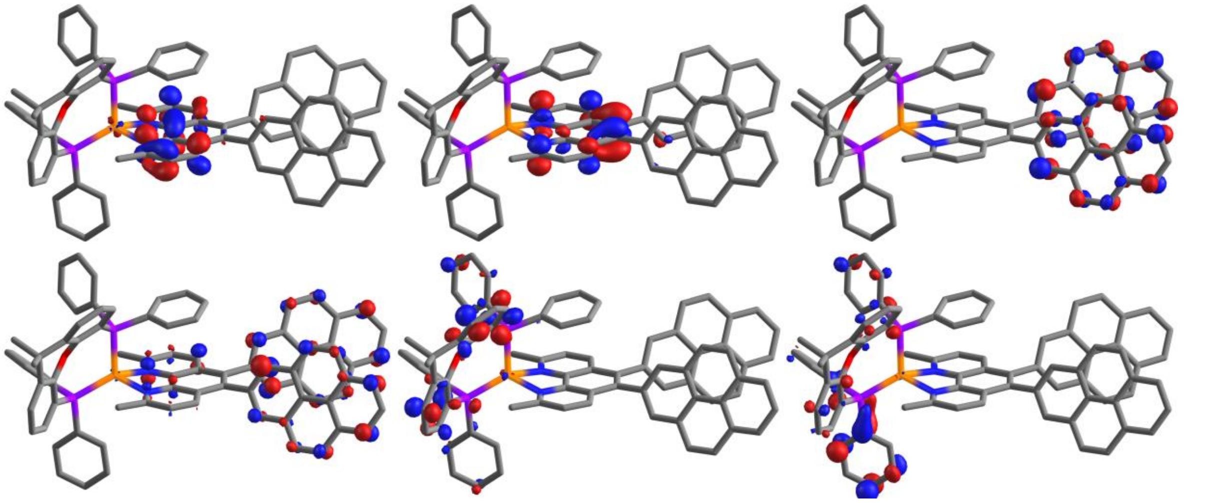

Figure S40. Orbital representation of **CuPyr3** showing the LUMO, LUMO+1, LUMO+2, LUMO+3, LUMO+4 and LUMO+5 (from top left to bottom right).

6.2 Predicted Excitation Spectra and Transitions

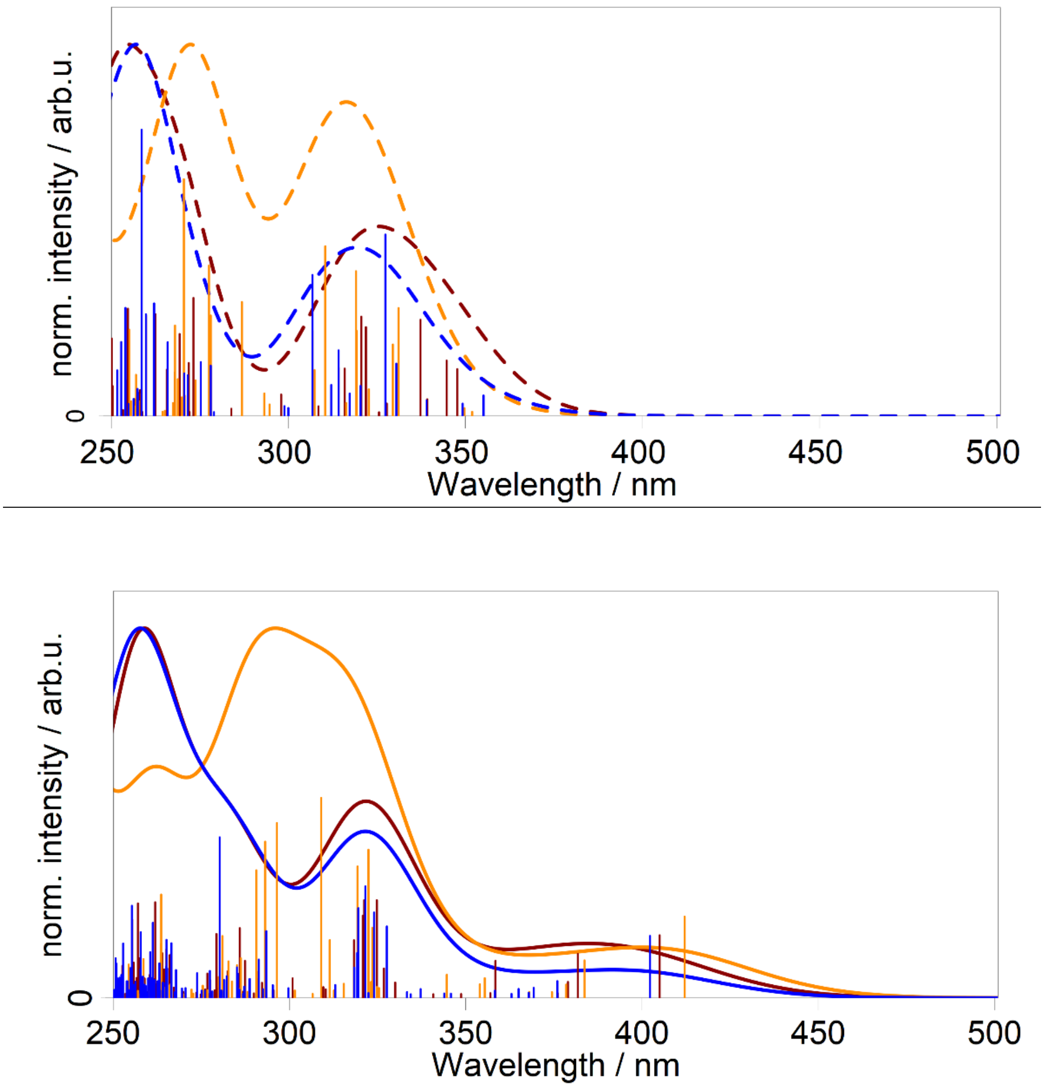

Figure S41. Calculated UV/vis spectra of the ligands **Pyr1-3** (dashed lines: red, orange, blue, respectively) and the complexes **CuPyr1-3** (solid lines: red, orange, blue, respectively) simulated in acetonitrile.

Table S1. Excitation energies, transition moments and corresponding transitions of **CuPyr1** obtained from TD-DFT simulated in acetonitrile. Note that only excitations with an oscillator strength > 0.01 and corresponding orbital contributions with an OC ≥ 0.1 ($|coeff.|^2 100 \geq 0.1$) are shown. Molecular fragments that show only minor contribution to the mixing of orbitals are given in parenthesis (*e.g.*(π_{Pyr})).

State	Exc. energy		Oscillator	Dominant contribution			Transition
#	cm^{-1}	nm	strength	OC	occ. orb.	virt. orb.	
1	24688	405.0	0.169	0.157	HOMO-1	LUMO	$d_{Cu},\pi_{pyr}\rightarrow\pi^*_{phen}$
				0.669	HOMO	LUMO	$d_{Cu},(\pi_{pyr})\rightarrow\pi^*_{phen}$
2	26191	381.8	0.119	0.606	HOMO	LUMO+1	$d_{Cu},(\pi_{pyr})\rightarrow\pi^*_{phen},\pi^*_{pyr}$
				0.103	HOMO	LUMO+3	$d_{Cu},(\pi_{pyr})\rightarrow\pi^*_{phen},\pi^*_{pyr}$
3	26388	379.0	0.043	0.423	HOMO-2	LUMO	$\pi_{pyr},(d_{Cu})\rightarrow\pi^*_{phen}$
				0.126	HOMO-2	LUMO+1	$\pi_{Pyr},(d_{Cu})\rightarrow\pi^*_{phen},\pi^*_{pyr}$
				0.137	HOMO	LUMO	$d_{Cu},(\pi_{Pyr})\rightarrow\pi^*_{phen}$
4	26586	376.1	0.013	0.111	HOMO-2	LUMO	$\pi_{pyr},(d_{Cu})\rightarrow\pi^*_{phen}$
				0.145	HOMO-2	LUMO+1	$\pi_{pyr},(d_{Cu})\rightarrow\pi^*_{phen},\pi^*_{pyr}$
				0.530	HOMO-1	LUMO	$d_{Cu},\pi_{pyr}\rightarrow\pi^*_{phen}$
6	27900	358.4	0.100	0.105	HOMO-2	LUMO+1	$\pi_{pyr},(d_{Cu})\rightarrow\pi^*_{phen},\pi^*_{pyr}$
				0.458	HOMO-1	LUMO+1	$d_{Cu},\pi_{pyr}\rightarrow\pi^*_{phen},\pi^*_{pyr}$
				0.188	HOMO	LUMO+1	$d_{Cu},(\pi_{pyr})\rightarrow\pi^*_{phen},\pi^*_{pyr}$
7	28013	357.0	0.013	0.241	HOMO-2	LUMO	$\pi_{pyr},(d_{Cu})\rightarrow\pi^*_{phen}$
				0.401	HOMO-2	LUMO+1	$\pi_{pyr},(d_{Cu})\rightarrow\pi^*_{phen},\pi^*_{pyr}$
				0.187	HOMO-1	LUMO+1	$d_{Cu},\pi_{pyr}\rightarrow\pi^*_{phen},\pi^*_{pyr}$
8	28679	348.7	0.010	0.426	HOMO-4	LUMO	$d_{Cu}\rightarrow\pi^*_{phen}$
				0.378	HOMO-3	LUMO	$d_{Cu}\rightarrow\pi^*_{phen}$
9	29341	340.8	0.010	0.796	HOMO	LUMO+2	$d_{Cu},(\pi_{pyr})\rightarrow\pi^*_{pyr}$
12	30303	330.0	0.042	0.718	HOMO-1	LUMO+2	$d_{Cu},\pi_{pyr}\rightarrow\pi^*_{pyr}$
				0.101	HOMO	LUMO+2	$d_{Cu},(\pi_{pyr})\rightarrow\pi^*_{pyr}$
13	30601	326.8	0.080	0.113	HOMO-2	LUMO+1	$\pi_{pyr},(d_{Cu})\rightarrow\pi^*_{phen},\pi^*_{pyr}$
				0.320	HOMO-2	LUMO+2	$\pi_{pyr},(d_{Cu})\rightarrow\pi^*_{pyr}$
				0.317	HOMO-2	LUMO+3	$\pi_{pyr},(d_{Cu})\rightarrow\pi^*_{phen},\pi^*_{pyr}$
14	30785	324.8	0.264	0.309	HOMO-2	LUMO+2	$\pi_{pyr},(d_{Cu})\rightarrow\pi^*_{pyr}$
				0.150	HOMO-2	LUMO+6	$\pi_{pyr},(d_{Cu})\rightarrow\pi^*_{pyr}$
15	30872	323.9	0.057	0.168	HOMO-6	LUMO+1	$\pi_{pyr},(\pi_{phen})\rightarrow\pi^*_{phen},\pi^*_{pyr}$
				0.305	HOMO-1	LUMO+7	$d_{Cu},\pi_{pyr}\rightarrow\pi^*_{pyr}$
				0.149	HOMO	LUMO+7	$d_{Cu},(\pi_{pyr})\rightarrow\pi^*_{pyr}$
17	31130	321.2	0.264	0.247	HOMO-1	LUMO+3	$d_{Cu},\pi_{pyr}\rightarrow\pi^*_{phen},\pi^*_{pyr}$
				0.108	HOMO-1	LUMO+4	$d_{Cu},\pi_{pyr}\rightarrow\pi^*_{xant}$
				0.337	HOMO	LUMO+4	$d_{Cu},(\pi_{pyr})\rightarrow\pi^*_{xant}$
18	31174	320.8	0.222	0.271	HOMO-1	LUMO+3	$d_{Cu},\pi_{pyr}\rightarrow\pi^*_{phen},\pi^*_{pyr}$
				0.300	HOMO	LUMO+4	$d_{Cu},(\pi_{pyr})\rightarrow\pi^*_{xant}$
19	31417	318.3	0.156	0.113	HOMO-7	LUMO+2	$\pi_{pyr}\rightarrow\pi^*_{pyr}$
				0.411	HOMO-2	LUMO+3	$\pi_{pyr},(d_{Cu})\rightarrow\pi^*_{phen},\pi^*_{pyr}$
				0.217	HOMO-2	LUMO+6	$\pi_{pyr},(d_{Cu})\rightarrow\pi^*_{pyr}$
20	32231	310.3	0.023	0.139	HOMO-1	LUMO+5	$d_{Cu},\pi_{pyr}\rightarrow\pi^*_{xant}$
				0.442	HOMO	LUMO+5	$d_{Cu},(\pi_{pyr})\rightarrow\pi^*_{xant}$
21	32296	309.6	0.029	0.190	HOMO-8	LUMO	$\pi_{phen}\rightarrow\pi^*_{phen}$
				0.176	HOMO-6	LUMO	$\pi_{pyr},(\pi_{phen})\rightarrow\pi^*_{phen}$
				0.195	HOMO	LUMO+5	$d_{Cu},(\pi_{pyr})\rightarrow\pi^*_{xant}$
23	33228	300.9	0.053	0.201	HOMO-1	LUMO+8	$d_{Cu},\pi_{pyr}\rightarrow\pi^*_{xant}$
				0.653	HOMO	LUMO+8	$d_{Cu},(\pi_{pyr})\rightarrow\pi^*_{xant}$
24	33840	295.5	0.020	0.123	HOMO-8	LUMO+1	$\pi_{phen}\rightarrow\pi^*_{phen},\pi^*_{pyr}$
				0.157	HOMO-7	LUMO	$\pi_{pyr}\rightarrow\pi^*_{phen}$
				0.110	HOMO-7	LUMO+1	$\pi_{pyr}\rightarrow\pi^*_{phen},\pi^*_{pyr}$
				0.114	HOMO-6	LUMO	$\pi_{pyr},(\pi_{phen})\rightarrow\pi^*_{phen}$
				0.115	HOMO-6	LUMO+1	$\pi_{pyr}\rightarrow\pi^*_{phen},\pi^*_{pyr}$
26	34226	292.2	0.041	0.191	HOMO-1	LUMO+9	$d_{Cu},\pi_{pyr}\rightarrow\pi^*_{xant}$
				0.611	HOMO	LUMO+9	$d_{Cu},(\pi_{pyr})\rightarrow\pi^*_{xant}$
27	34371	290.9	0.051	0.184	HOMO-7	LUMO	$\pi_{pyr}\rightarrow\pi^*_{phen}$
				0.446	HOMO-5	LUMO+1	π^*_{phen},π^*_{pyr}
28	34490	289.9	0.012	0.217	HOMO-8	LUMO	$\pi_{phen}\rightarrow\pi^*_{phen}$
				0.132	HOMO-7	LUMO+1	$\pi_{pyr}\rightarrow\pi^*_{phen},\pi^*_{pyr}$
				0.235	HOMO-6	LUMO	$\pi_{pyr},(\pi_{phen})\rightarrow\pi^*_{phen}$
30	34790	287.4	0.100	0.112	HOMO-8	LUMO+1	$\pi_{phen}\rightarrow\pi^*_{phen},\pi^*_{pyr}$
				0.144	HOMO-6	LUMO+1	$\pi_{pyr},(\pi_{phen})\rightarrow\pi^*_{phen},\pi^*_{pyr}$
				0.143	HOMO-3	LUMO+3	$d_{Cu}\rightarrow\pi^*_{phen},\pi^*_{pyr}$

Table S2. Excitation energies, transition moments and corresponding transitions of **CuPyr2** obtained from TD-DFT simulated in acetonitrile. Note that only excitations with an oscillator strength > 0.01 and corresponding orbital contributions with an OC ≥ 0.1 ($|coeff.|^2 100 \geq 0.1$) are shown. Molecular fragments that show only minor contribution to the mixing of orbitals are given in parenthesis (*e.g.*(π_{pyr}).

State	Exc. energy		Oscillator	Dominant contribution			Transition
#	cm^{-1}	nm	strength	OC	occ. orb.	virt. orb.	
1	24266	412	0.220	0.910	HOMO	LUMO	$d_{Cu}\rightarrow\pi^*_{phen}$
2	26063	384	0.102	0.763	HOMO	LUMO+1	$d_{Cu}\rightarrow\pi^*_{phen}$
3	26417	379	0.036	0.788	HOMO-2	LUMO	$\pi_{pyr}\rightarrow\pi^*_{phen}$
4	26705	375	0.016	0.325	HOMO-4	LUMO	$d_{Cu}\rightarrow\pi^*_{phen}$
				0.498	HOMO-3	LUMO	$\pi_{pyr}\rightarrow\pi^*_{phen}$
5	27176	368	0.016	0.598	HOMO-1	LUMO	$\pi_{pyr}\rightarrow\pi^*_{phen}$
				0.284	HOMO-1	LUMO+1	$\pi_{pyr}\rightarrow\pi^*_{phen}$
6	28139	355	0.053	0.362	HOMO-1	LUMO	$\pi_{pyr}\rightarrow\pi^*_{phen}$
				0.467	HOMO-1	LUMO+1	$\pi_{pyr}\rightarrow\pi^*_{phen}$
7	28246	354	0.037	0.445	HOMO-4	LUMO	$d_{Cu}\rightarrow\pi^*_{phen}$
				0.375	HOMO-3	LUMO	$\pi_{pyr}\rightarrow\pi^*_{phen}$
8	29021	345	0.063	0.802	HOMO-2	LUMO+1	$\pi_{pyr}\rightarrow\pi^*_{phen}$
12	30729	325	0.027	0.217	HOMO-4	LUMO+1	$d_{Cu}\rightarrow\pi^*_{phen}$
				0.214	HOMO-3	LUMO+1	$\pi_{pyr}\rightarrow\pi^*_{phen}$
				0.407	HOMO	LUMO+4	$d_{Cu}\rightarrow\pi^*_{xant}$
13	30789	325	0.030	0.174	HOMO-4	LUMO+1	$d_{Cu}\rightarrow\pi^*_{phen}$
				0.155	HOMO-3	LUMO+1	$\pi_{pyr}\rightarrow\pi^*_{phen}$
				0.542	HOMO	LUMO+4	$d_{Cu}\rightarrow\pi^*_{xant}$
14	30916	324	0.190	0.264	HOMO-2	LUMO+2	$\pi_{pyr}\rightarrow\pi^*_{pyr}$
				0.329	HOMO-2	LUMO+3	$\pi_{pyr}\rightarrow\pi^*_{pyr},(\pi^*_{phen})$
15	30949	323	0.043	0.617	HOMO-1	LUMO+2	$\pi_{pyr}\rightarrow\pi^*_{pyr}$
				0.174	HOMO-1	LUMO+3	$\pi_{pyr}\rightarrow\pi^*_{pyr},(\pi^*_{phen})$
17	31017	322	0.401	0.120	HOMO-5	LUMO	$d_{Cu}\rightarrow\pi^*_{phen}$
				0.172	HOMO-1	LUMO+2	$\pi_{pyr}\rightarrow\pi^*_{pyr}$
				0.448	HOMO-1	LUMO+3	$\pi_{pyr}\rightarrow\pi^*_{pyr},(\pi^*_{phen})$
18	31318	319	0.356	0.421	HOMO-5	LUMO	$d_{Cu}\rightarrow\pi^*_{phen}$
19	31709	315	0.038	0.908	HOMO	LUMO+5	$d_{Cu}\rightarrow\pi^*_{xant}$
20	31989	313	0.020	0.111	HOMO-7	LUMO+2	$\pi_{pyr},(\pi_{phen})\rightarrow\pi^*_{pyr}$
				0.133	HOMO-1	LUMO+3	$\pi_{pyr}\rightarrow\pi^*_{pyr},(\pi^*_{phen})$
				0.130	HOMO-1	LUMO+6	$\pi_{pyr}\rightarrow\pi^*_{phen},\pi^*_{pyr}$
				0.258	HOMO-1	LUMO+7	$\pi_{pyr}\rightarrow\pi^*_{phen},\pi^*_{pyr}$
21	32112	311	0.156	0.118	HOMO-5	LUMO+1	$d_{Cu}\rightarrow\pi^*_{phen}$
				0.160	HOMO-2	LUMO+3	$\pi_{pyr}\rightarrow\pi^*_{pyr},(\pi^*_{phen})$
				0.189	HOMO-2	LUMO+6	$\pi_{pyr}\rightarrow\pi^*_{phen},\pi^*_{pyr}$
				0.106	HOMO-2	LUMO+7	$\pi_{pyr}\rightarrow\pi^*_{phen},\pi^*_{pyr}$
22	32362	309	0.541	0.162	HOMO-7	LUMO	$\pi_{pyr},(\pi_{phen})\rightarrow\pi^*_{phen}$
				0.288	HOMO-5	LUMO+1	$d_{Cu}\rightarrow\pi^*_{phen}$
23	32619	307	0.011	0.747	HOMO-6	LUMO	$\pi^*_{xant},(d_{Cu})\rightarrow\pi^*_{phen}$
				0.178	HOMO-5	LUMO	$d_{Cu}\rightarrow\pi^*_{phen}$
24	33166	302	0.020	0.280	HOMO	LUMO+6	$d_{Cu}\rightarrow\pi^*_{phen},\pi^*_{pyr}$
					HOMO	LUMO+8	$d_{Cu}\rightarrow\pi^*_{xant}$
					HOMO	LUMO+9	$d_{Cu}\rightarrow\pi^*_{xant}$
25	33740	296	0.473	0.201	HOMO	LUMO+6	$d_{Cu}\rightarrow\pi^*_{phen},\pi^*_{pyr}$
				0.327	HOMO	LUMO+8	$d_{Cu}\rightarrow\pi^*_{xant}$
26	33875	295	0.021	0.498	HOMO-7	LUMO	$\pi_{Pyr},(\pi_{Phen})\rightarrow\pi^*_{phen}$
27	34119	293	0.423	0.536	HOMO	LUMO+9	$d_{Cu}\rightarrow\pi^*_{xant}$
28	34403	291	0.071	0.445	HOMO-3	LUMO+2	$\pi_{pyr}\rightarrow\pi^*_{pyr}$
					HOMO-3	LUMO+3	$\pi_{pyr}\rightarrow\pi^*_{pyr},(\pi^*_{phen})$
29	34415	291	0.345	0.107	HOMO-8	LUMO	$\pi_{phen},(\pi_{pyr})\rightarrow\pi^*_{phen}$
					HOMO-7	LUMO+1	$\pi_{pyr},(\pi_{phen})\rightarrow\pi^*_{phen}$
					HOMO-3	LUMO+2	$\pi_{pyr}\rightarrow\pi^*_{pyr}$

Table S3. Excitation energies, transition moments and corresponding transitions of **CuPyr3** obtained from TD-DFT simulated in acetonitrile. Note that only excitations with an oscillator strength > 0.01 and corresponding orbital contributions with an OC ≥ 0.1 ($|coeff.|^2 100 \geq 0.1$) are shown. Molecular fragments that show only minor contribution to the mixing of orbitals are given in parenthesis (*e.g.*(π_{pyr}).

State	Exc. energy		Oscillator	Dominant contribution			Transition
#	cm^{-1}	nm	strength	OC	occ. orb.	virt. orb.	
1	24859	402.3	0.168	0.215	HOMO-2	LUMO	$d_{Cu},\pi_{pyr}\rightarrow\pi^*_{phen}$
				0.675	HOMO	LUMO	$d_{Cu},(\pi_{pyr})\rightarrow\pi^*_{phen}$
2	26598	376.0	0.046	0.182	HOMO-2	LUMO+1	$d_{Cu},\pi_{pyr}\rightarrow\pi^*_{phen}$
				0.697	HOMO	LUMO+1	$d_{Cu},(\pi_{pyr})\rightarrow\pi^*_{phen}$
3	27080	369.3	0.027	0.178	HOMO-5	LUMO	$d_{Cu}\rightarrow\pi^*_{phen}$
				0.286	HOMO-4	LUMO	$d_{Cu}\rightarrow\pi^*_{phen}$
				0.170	HOMO-2	LUMO	$d_{Cu},\pi_{pyr}\rightarrow\pi^*_{phen}$
				0.120	HOMO	LUMO	$d_{Cu},(\pi_{pyr})\rightarrow\pi^*_{phen}$
4	27184	367.9	0.014	0.112	HOMO-5	LUMO	$d_{Cu}\rightarrow\pi^*_{phen}$
				0.178	HOMO-4	LUMO	$d_{Cu}\rightarrow\pi^*_{phen}$
				0.446	HOMO-1	LUMO	$\pi_{pyr}\rightarrow\pi^*_{phen}$
5	27399	365.0	0.024	0.340	HOMO-2	LUMO	$d_{Cu},\pi_{pyr}\rightarrow\pi^*_{phen}$
				0.387	HOMO-1	LUMO	$\pi_{pyr}\rightarrow\pi^*_{phen}$
6	27551	363.0	0.011	0.791	HOMO-1	LUMO+1	$\pi_{pyr}\rightarrow\pi^*_{phen}$
7	27906	358.3	0.020	0.108	HOMO-2	LUMO	$d_{Cu},\pi_{pyr}\rightarrow\pi^*_{phen}$
				0.559	HOMO-2	LUMO+1	$d_{Cu},\pi_{pyr}\rightarrow\pi^*_{phen}$
				0.175	HOMO	LUMO+1	$d_{Cu},(\pi_{pyr})\rightarrow\pi^*_{phen}$
9	28921	345.8	0.012	0.446	HOMO-5	LUMO	$d_{Cu}\rightarrow\pi^*_{phen}$
				0.282	HOMO-4	LUMO	$d_{Cu}\rightarrow\pi^*_{phen}$
10	29074	343.9	0.012	0.105	HOMO-2	LUMO+3	$d_{Cu},\pi_{pyr}\rightarrow\pi^*_{pyr},(\pi^*_{phen})$
				0.370	HOMO-1	LUMO+2	$\pi_{pyr}\rightarrow\pi^*_{pyr}$
				0.171	HOMO-1	LUMO+3	$\pi_{pyr}\rightarrow\pi^*_{pyr},(\pi^*_{phen})$
				0.187	HOMO	LUMO+3	$d_{Cu},(\pi_{pyr})\rightarrow\pi^*_{pyr},(\pi^*_{phen})$
11	29656	337.2	0.024	0.508	HOMO-3	LUMO	$d_{Cu},\pi_{phen},\pi_{pyr}\rightarrow\pi^*_{phen}$
				0.150	HOMO-2	LUMO+3	$d_{Cu},\pi_{pyr}\rightarrow\pi^*_{pyr},(\pi^*_{phen})$
12	29901	334.4	0.010	0.259	HOMO-5	LUMO+1	$d_{Cu}\rightarrow\pi^*_{phen}$
				0.486	HOMO-4	LUMO+1	$d_{Cu}\rightarrow\pi^*_{phen}$
13	30000	333.3	0.016	0.129	HOMO-2	LUMO+2	$d_{Cu},\pi_{pyr}\rightarrow\pi^*_{pyr}$
				0.665	HOMO	LUMO+2	$d_{Cu},(\pi_{pyr})\rightarrow\pi^*_{pyr}$
15	30522	327.6	0.193	0.146	HOMO-2	LUMO+2	$d_{Cu},\pi_{pyr}\rightarrow\pi^*_{pyr}$
				0.120	HOMO-1	LUMO+2	$\pi_{pyr}\rightarrow\pi^*_{pyr}$
				0.406	HOMO-1	LUMO+3	$\pi_{pyr}\rightarrow\pi^*_{pyr},(\pi^*_{phen})$
16	30868	324.0	0.231	0.532	HOMO-3	LUMO+1	$d_{Cu},\pi_{phen},\pi_{pyr}\rightarrow\pi^*_{phen}$
				0.103	HOMO-2	LUMO+2	$d_{Cu},\pi_{pyr}\rightarrow\pi^*_{pyr}$
				0.100	HOMO	LUMO+3	$d_{Cu},(\pi_{pyr})\rightarrow\pi^*_{pyr},(\pi^*_{phen})$
17	31016	322.4	0.013	0.241	HOMO-2	LUMO+4	$d_{Cu},\pi_{pyr}\rightarrow\pi^*_{xant}$
				0.623	HOMO	LUMO+4	$d_{Cu},(\pi_{pyr})\rightarrow\pi^*_{xant}$
18	31105	321.5	0.302	0.127	HOMO-3	LUMO+1	$d_{Cu},\pi_{phen},\pi_{pyr}\rightarrow\pi^*_{phen}$
				0.172	HOMO-3	LUMO+3	$d_{Cu},\pi_{phen},\pi_{pyr}\rightarrow\pi^*_{Pyr},(\pi^*_{phen})$
				0.110	HOMO-1	LUMO+2	$\pi_{pyr}\rightarrow\pi^*_{pyr}$
				0.109	HOMO	LUMO+3	$d_{Cu},(\pi_{pyr})\rightarrow\pi^*_{pyr},(\pi^*_{phen})$
19	31303	319.5	0.243	0.133	HOMO-2	LUMO+2	$d_{Cu},\pi_{pyr}\rightarrow\pi^*_{pyr}$
20	31325	319.2	0.121	0.371	HOMO-5	LUMO+1	$d_{Cu}\rightarrow\pi^*_{phen}$
				0.210	HOMO-4	LUMO+1	$d_{Cu}\rightarrow\pi^*_{phen}$
21	31411	318.4	0.082	0.122	HOMO-3	LUMO+1	$d_{Cu},\pi_{phen},\pi_{pyr}\rightarrow\pi^*_{phen}$
				0.166	HOMO-3	LUMO+2	$d_{Cu},\pi_{phen},\pi_{pyr}\rightarrow\pi^*_{pyr}$
				0.153	HOMO-1	LUMO+7	$\pi_{pyr}\rightarrow\pi^*_{pyr}$
22	31948	313.0	0.035	0.236	HOMO-2	LUMO+5	$d_{Cu},\pi_{pyr}\rightarrow\pi^*_{xant}$
				0.626	HOMO	LUMO+5	$d_{Cu},(\pi_{pyr})\rightarrow\pi^*_{xant}$
24	33368	299.7	0.026	0.156	HOMO-2	LUMO+8	$d_{Cu},\pi_{pyr}\rightarrow\pi^*_{xant}$
				0.400	HOMO	LUMO+8	$d_{Cu},(\pi_{Pyr})\rightarrow\pi^*_{xant}$
25	33865	295.3	0.035	0.170	HOMO-7	LUMO	$\pi_{pyr}\rightarrow\pi^*_{phen}$
				0.335	HOMO-3	LUMO+2	$d_{Cu},\pi_{phen},\pi_{pyr}\rightarrow\pi^*_{pyr}$

				0.137	HOMO-3	LUMO+3	$d_{Cu},\pi_{phen},\pi_{pyr}\rightarrow\pi^*_{pyr}$, (π^*_{phen})
26	34086	293.4	0.181	0.106	HOMO-7	LUMO+2	$\pi_{Pyr}\rightarrow\pi^*_{Pyr}$
				0.200	HOMO-3	LUMO+3	$d_{Cu},\pi_{Phen},\pi_{Pyr}\rightarrow\pi^*_{Pyr},(\pi^*_{phen})$
				0.104	HOMO-1	LUMO+7	$\pi_{pyr}\rightarrow\pi^*_{pyr}$
27	34182	292.5	0.024	0.145	HOMO-7	LUMO+1	$\pi_{pyr}\rightarrow\pi^*_{phen}$
				0.116	HOMO-3	LUMO+3	$d_{Cu},\pi_{phen},\pi_{pyr}\rightarrow\pi^*_{pyr}$, (π^*_{phen})
				0.109	HOMO	LUMO+8	$d_{Cu},(\pi_{pyr})\rightarrow\pi^*_{xant}$
28	34331	291.3	0.104	0.197	HOMO-2	LUMO+9	$d_{Cu},\pi_{pyr}\rightarrow\pi^*_{xant}$
				0.532	HOMO	LUMO+9	$d_{Cu},(\pi_{pyr})\rightarrow\pi^*_{xant}$
29	34637	288.7	0.052	0.116	HOMO-6	LUMO+1	$d_{Cu},\pi_{xant}\rightarrow\pi^*_{phen}$
				0.211	HOMO	LUMO+7	$d_{Cu},(\pi_{pyr})\rightarrow\pi^*_{pyr}$
30	34790	287.4	0.100	0.112	HOMO-8	LUMO+1	$d_{Cu},\pi_{phen},(\pi_{pyr})\rightarrow\pi^*_{phen}$
				0.144	HOMO-6	LUMO+1	$d_{Cu},\pi_{xant}\rightarrow\pi^*_{phen}$
				0.143	HOMO-3	LUMO+3	$d_{Cu},\pi_{phen},\pi_{pyr}\rightarrow\pi^*_{pyr}$, (π^*_{phen})
31	34867	286.8	0.017	0.701	HOMO-4	LUMO+2	$d_{Cu}\rightarrow\pi^*_{pyr}$
				0.106	HOMO-3	LUMO+2	$d_{Cu},\pi_{phen},\pi_{pyr}\rightarrow\pi^*_{Pyr}$
32	34983	285.9	0.189	0.130	HOMO-1	LUMO+10	$\pi_{Pyr}\rightarrow\pi^*_{xant}$
				0.440	HOMO	LUMO+10	$d_{Cu},(\pi_{pyr})\rightarrow\pi^*_{xant}$

7 UV/vis Absorption

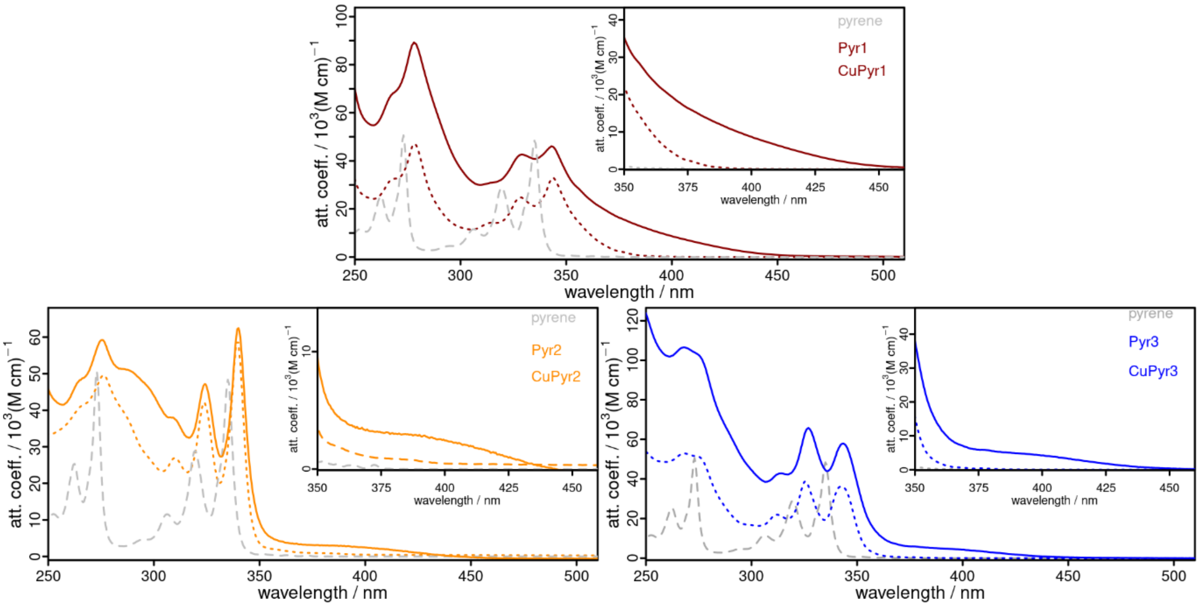

Figure S42. Experimental UV/vis absorption spectra in aerated acetonitrile of pyrene (grey) and
Top: **Pyr1** (dark red, dotted) and **CuPyr1** (dark red, solid)
Left: **Pyr2** (orange, dotted) and **CuPyr2** (orange, solid)
Right: **Pyr3** (blue, dotted) and **CuPyr3** (blue, solid)

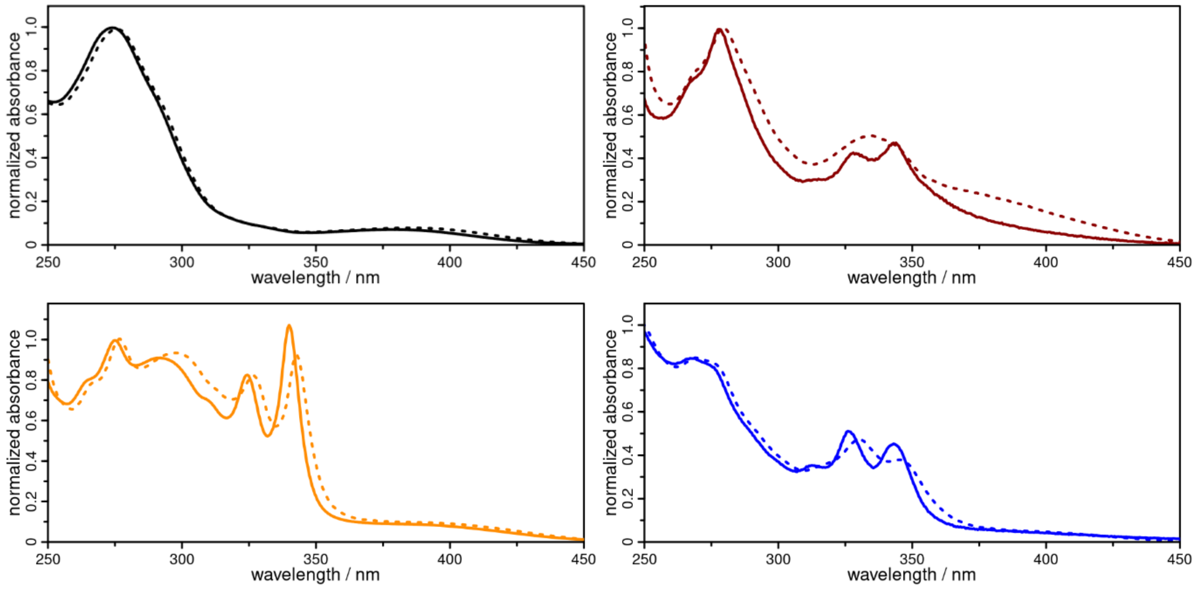

Figure S43. Normalized absorption spectra of **CuNeo** (black), **CuPyr1** (dark red), **CuPyr2** (orange), **CuPyr3** (blue) in acetonitrile (solid lines) and in dichloromethane (dotted lines) under inert conditions.

8 Steady-State Emission

8.1 Emission Spectra of Ligands in Acetonitrile and in Dichloromethane

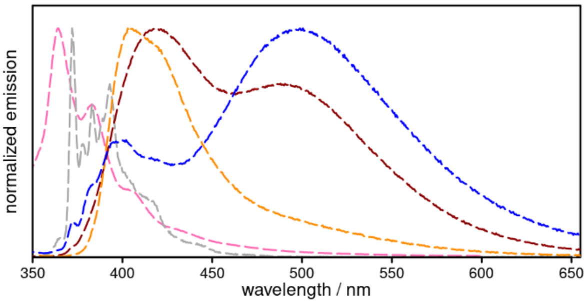

Figure S44. Emission spectra of **Neo** (pink), pyrene (grey), **Pyr1** (red), **Pyr2** (orange) and **Pyr3** (blue) with 334 nm excitation wavelengths recorded in dichloromethane under inert conditions.

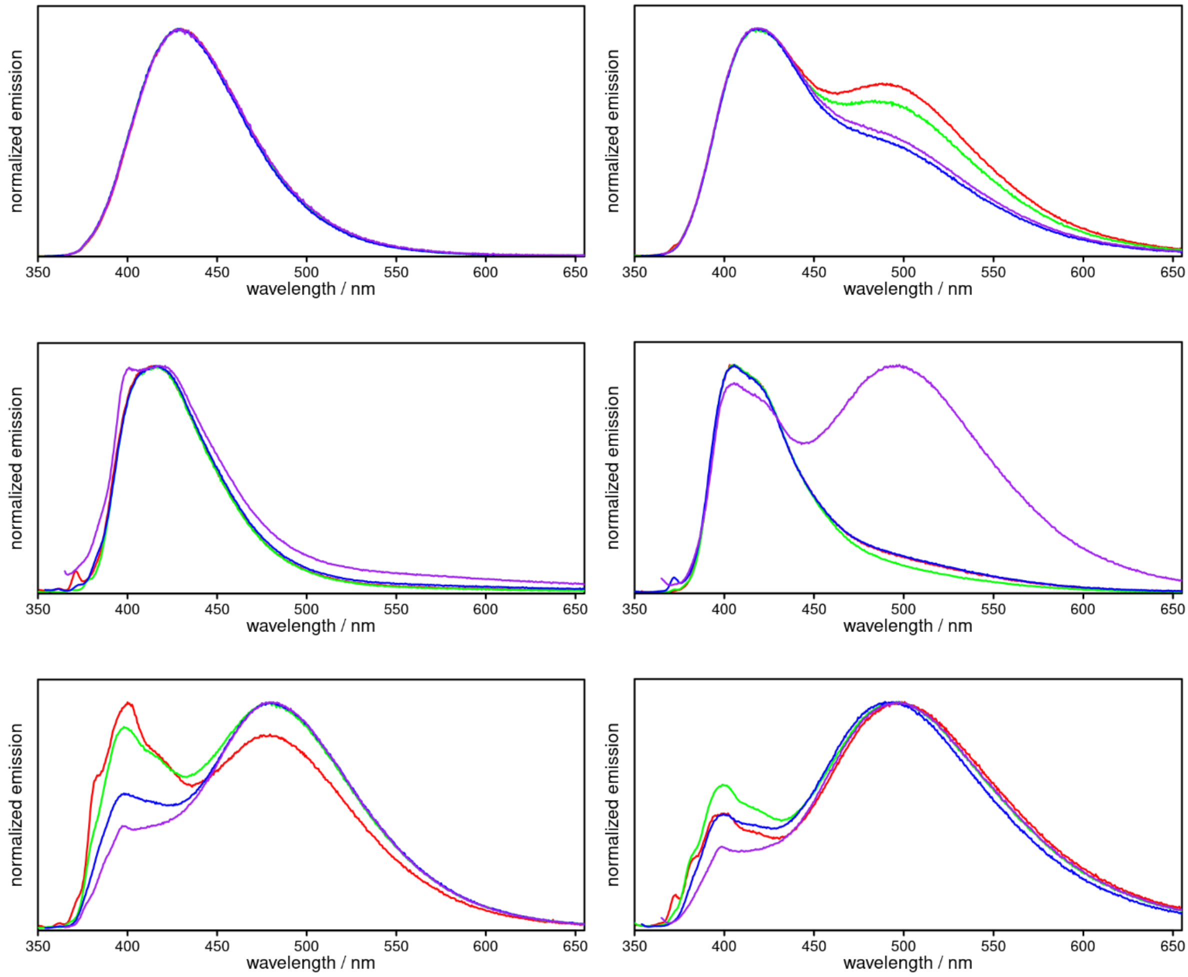

Figure S45. Room temperature emission spectra of **Pyr1** (top**), Pyr2** (middle), **Pyr3** (bottom) with different excitation wavelength, 334 nm (red), 340 nm (green), 344 nm (blue), 355 nm(purple), recorded in acetonitrile (left) and in dichloromethane (right) under inert conditions.

8.2 Emission Spectra of Ligands With and Without Trifluoroacetic acid

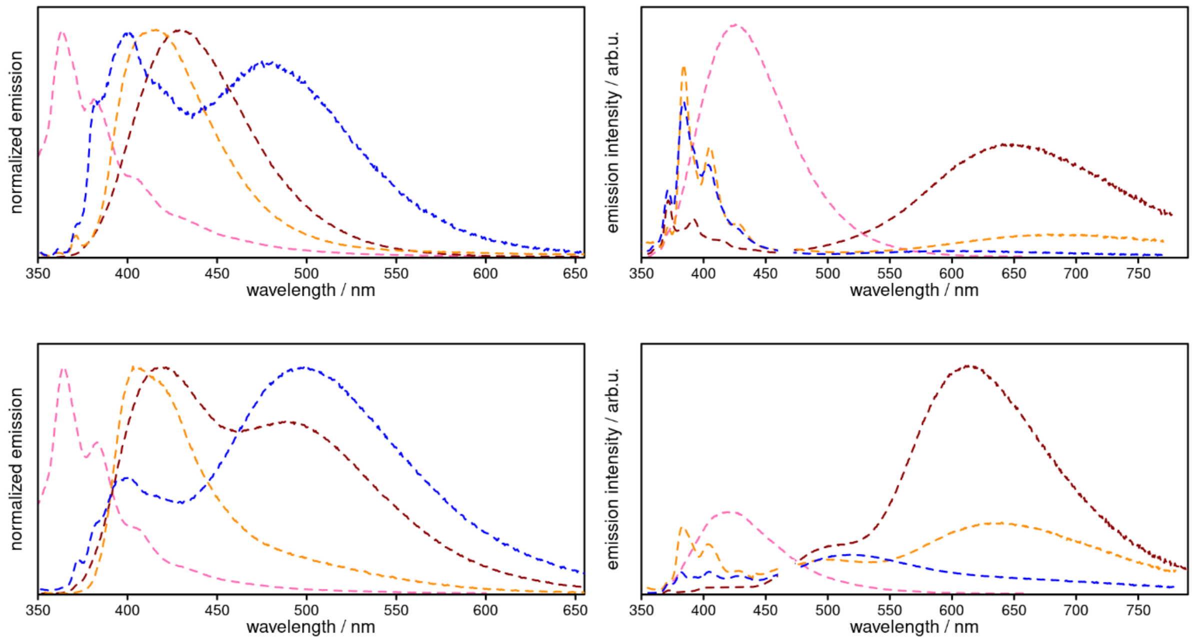

Figure S46. Emission spectra of **Neo**(pink), **Pyr1** (red), **Pyr2** (orange) and **Pyr3** (blue) with 334 nm excitation wavelengths recorded in
Top left: acetonitrile
Top right: acid acetonitrile (trifluoroacetic acid, 100 equivalent)
Bottom left: dichloromethane
Bottom right: acid dichloromethane (trifluoroacetic acid, 100 equivalent)
The emission intensity of **Pyr1**, **Pyr2** and **Pyr3** in acid solvent is respect to the absorbance at the excitation wavelength. The Emission measurements from 350 to 460nm were without 400 nm long pass filter and from 470 to 800 nm were with 400 nm long pass filter.

Table S4. Summary of the emission maxima of pyrene-based ligands (**Pyr1-3**) and reference compounds (**Neo**) in different solutions under inert conditions excited at 334 nm.

compound	$\lambda_{em,max}$ [nm]		$\lambda_{em,max}$ [nm]	
	acetonitrile	acetonitrile with 100 eq trifluoroacetic acid	dichloromethane	dichloromethane with 100 eq trifluoroacetic acid
**Neo**	363	425	364	420
**Pyr1**	430	647	420,494	503, 613
**Pyr2**	416	685	405	498, 636
**Pyr3**	400, 480	601	398, 498	515

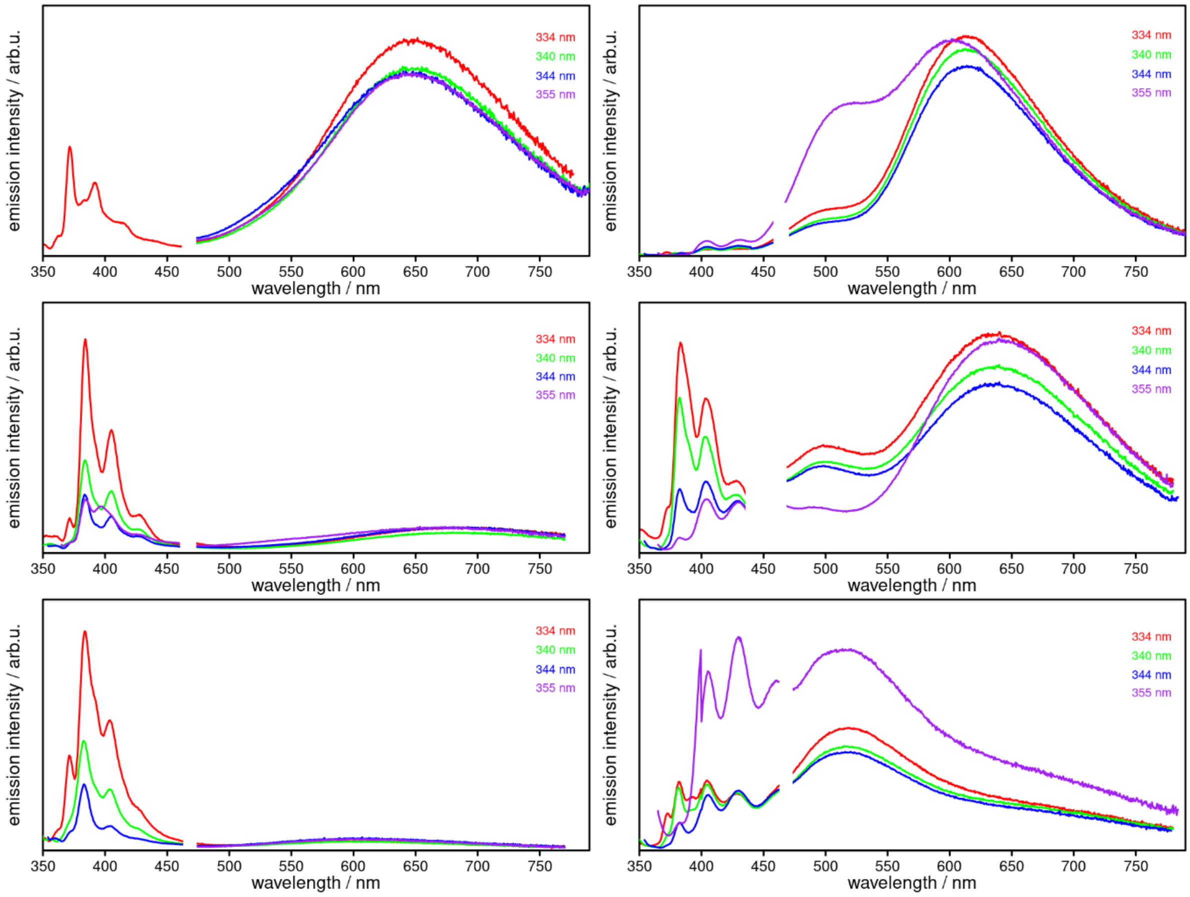

Figure S47. Emission spectra with different excitation wavelength, 334 nm (red), 340 nm (green), 344 nm (blue), 355 nm (purple), recorded of **Pyr1** (top), **Pyr2** (middle) and **Pyr3** (bottom)

Left: in acid acetonitrile (trifluoroacetic acid, 100 equivalent)

Right: in acid dichloromethane (trifluoroacetic acid, 100 equivalent)

The emission intensity is respect to the absorbance at the excitation wavelength. The Emission measurements from 350 to 460nm were without 400 nm long pass filter and from 470 to 800 nm were with 400 nm long pass filter.

8.3 Emission Spectra of Complexes

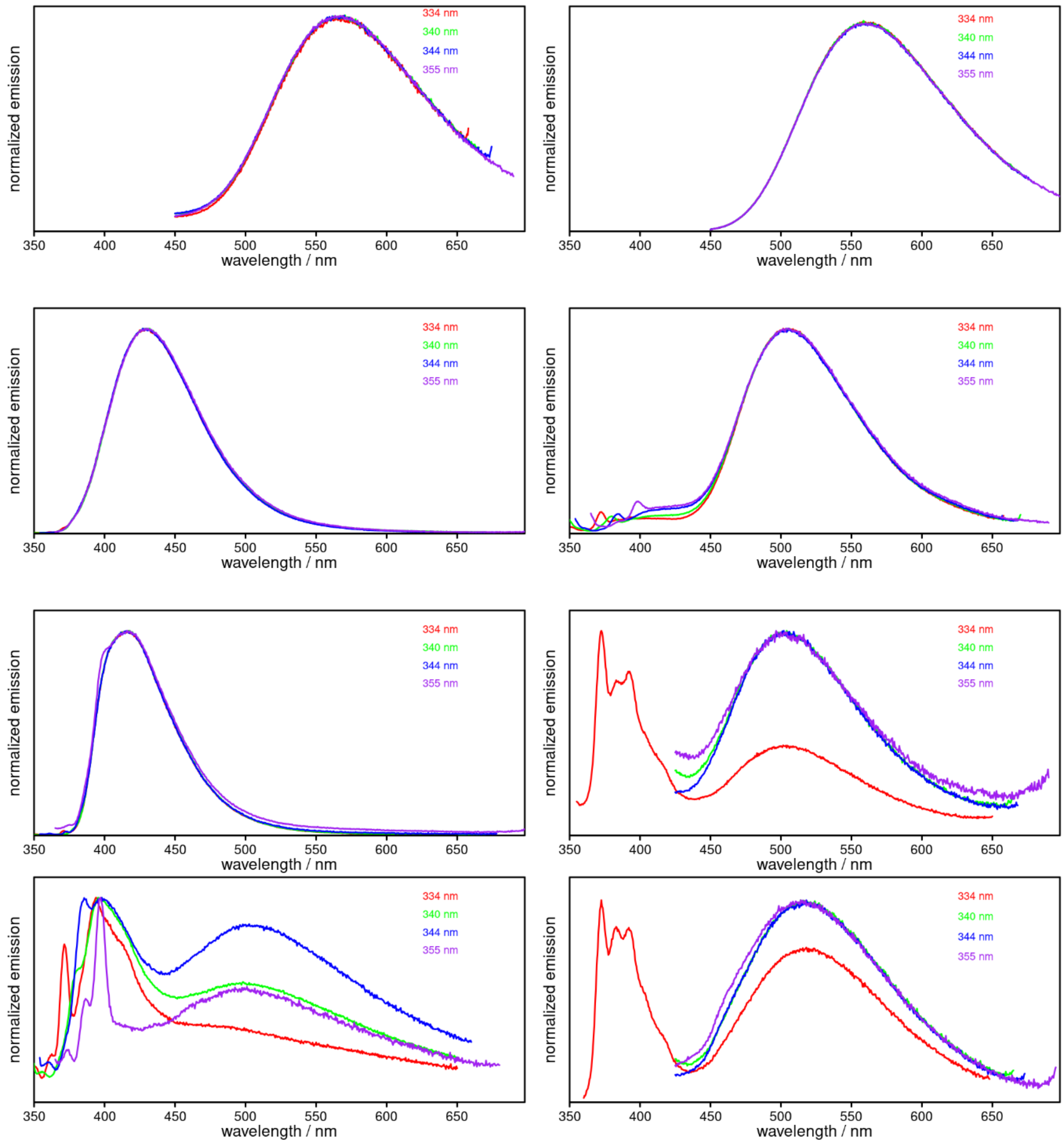

Figure S48. Emission spectra of **CuNeo** (top), **CuPyr1** (second), **CuPyr2** (third) and **CuPyr3** (bottom) with different excitation wavelength, 334 nm (red), 340 nm (green), 344 nm (blue), 355 nm(purple) recorded in acetonitrile (left) and in dichloromethane (right) under inert conditions

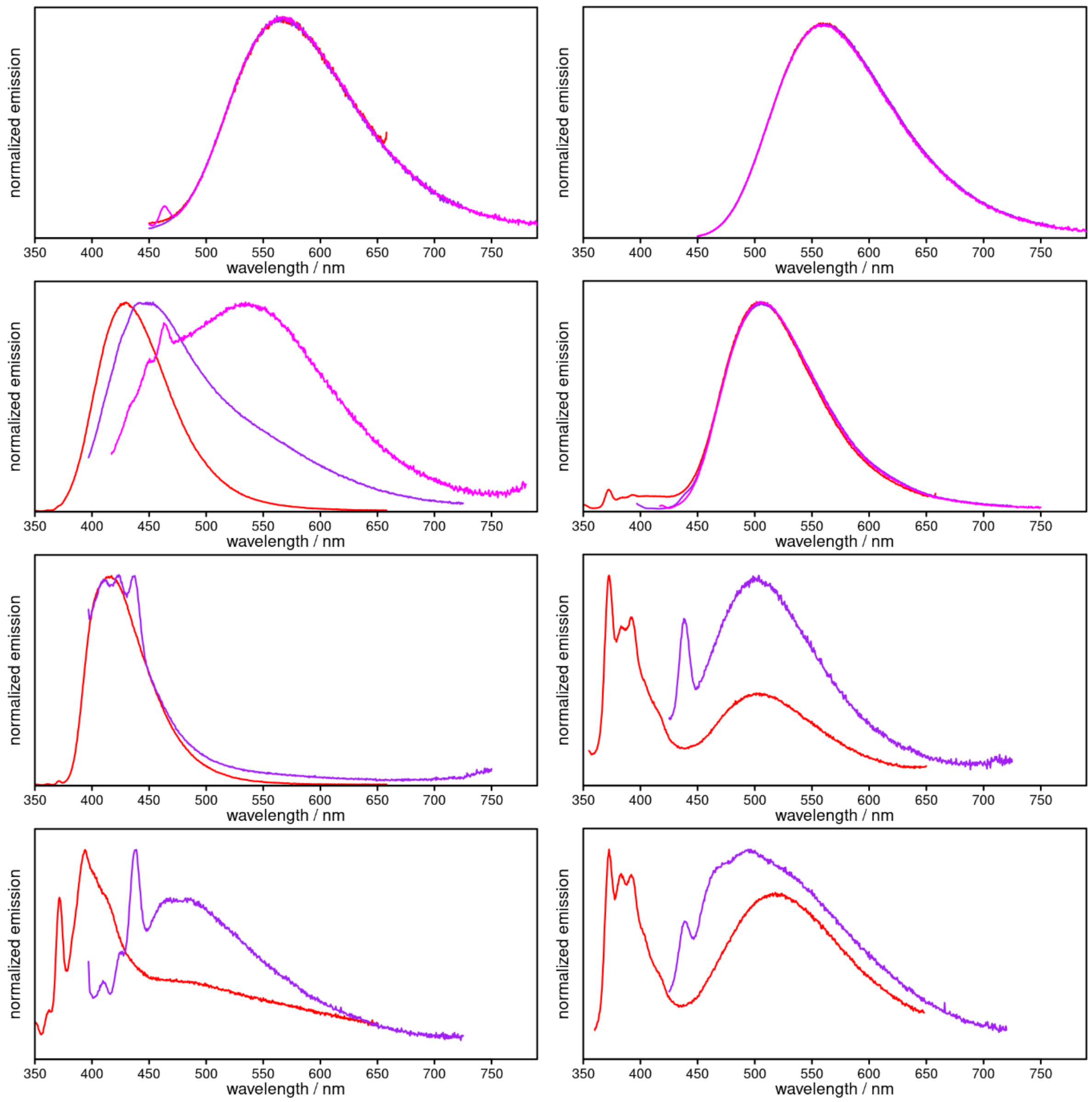

Figure S49. Emission spectra of **CuNeo** (top), **CuPyr1** (second), **CuPyr2** (third) and **CuPyr3** (bottom) with different excitation wavelength, 334 nm (red), 387 nm(purple), 407 (magenta) recorded in acetonitrile (left) and in dichloromethane (right) under inert conditions. Due to weak emission of **CuPyr2** and **CuPyr3** with excitation wavelength 407 nm, the results are not showed.

9. Transient Absorption

Table S5. Summary of averaged excited state lifetime of all pyrene-based ligands (**Pyr1-3**), complexes (**CuPyr1-3**) and of reference compounds (**CuNeo**) in acetonitrile and dichloromethane solution under inert conditions. All data were obtained at room temperature.

Compound		**CuNeo**	**Pyr1**	**CuPyr1**	**Pyr2**	**CuPyr2**	**Pyr3**	**CuPyr3**
τ [μs]	acetonitrile	0.20	16.03	22.42	3.71	17.70	8.47	1.15, 5.81
	dichloromethane	4.12	26.43	35.95	9.86	25.02	-	1.19, 2.62

- signal with large noise, sample not stable.

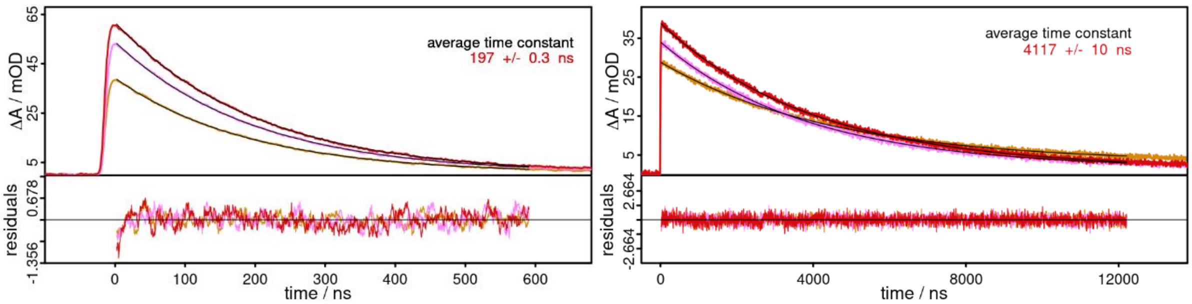

Figure S50. Single wavelength kinetic analysis at 480 (dark yellow), 500 (magenta) and 520 nm (red) of **CuNeo** excited at 355 nm under inert conditions
Left: in acetonitrile
Right: in dichloromethane
The black line belongs to the fits.

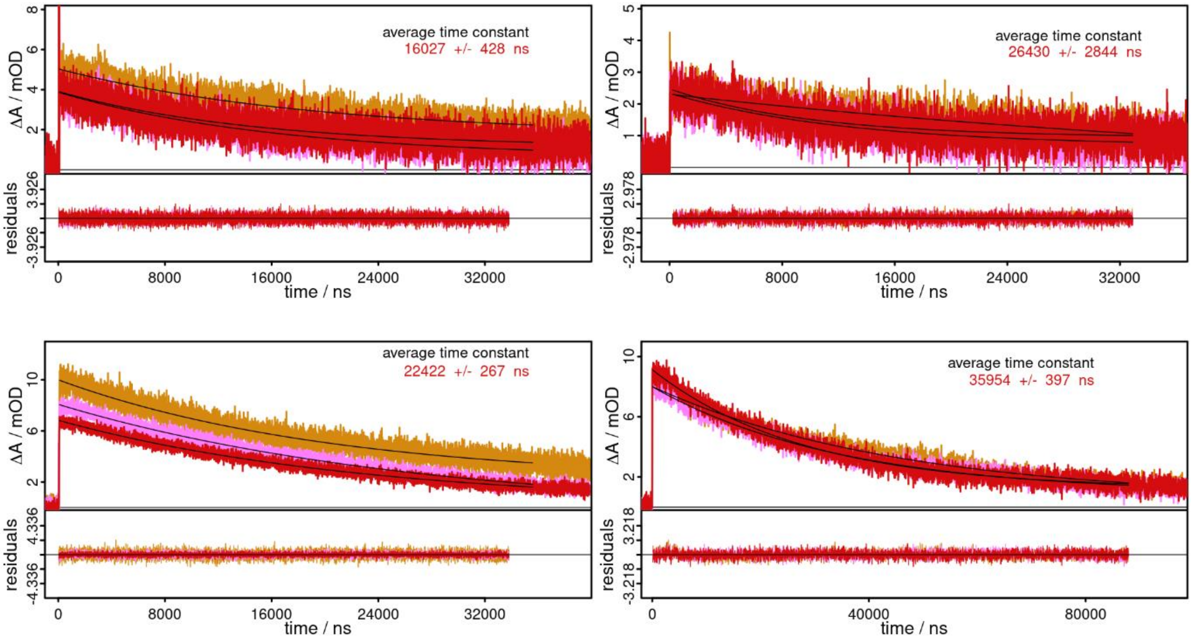

Figure S51. Single wavelength kinetic analysis at 480 (dark yellow), 500 (magenta) and 520 nm (red) of **Pyr1** (top) and **CuPyr1** (bottom) excited at 355 nm under inert conditions
Left: in acetonitrile
Right: in dichloromethane
The black line belongs to the fits.

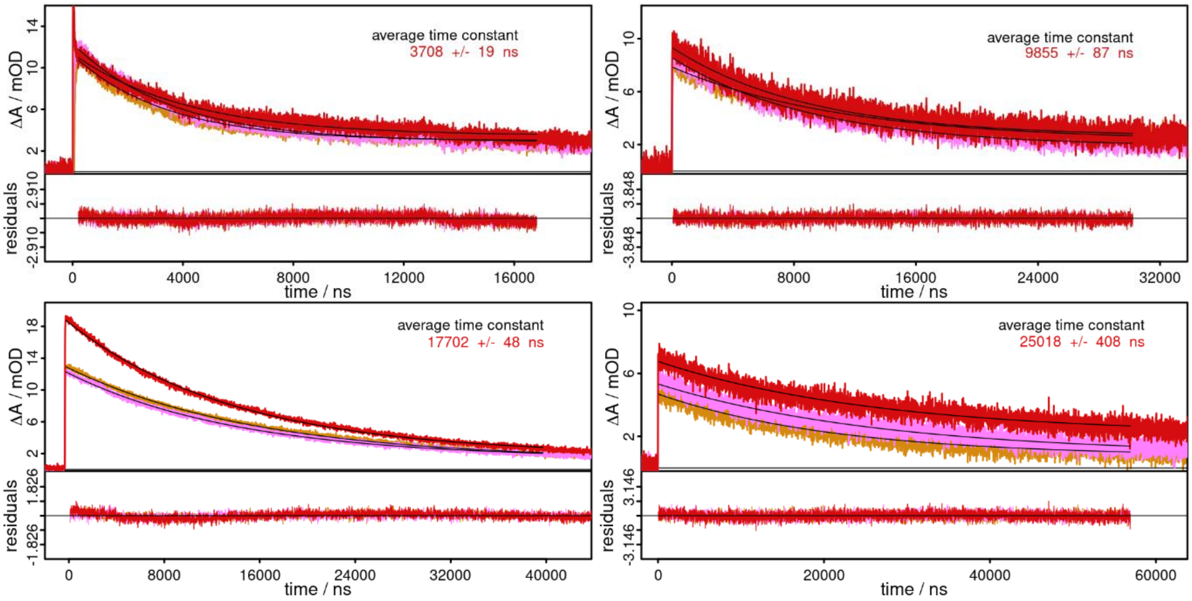

Figure S52. Single wavelength kinetic analysis at 480 (dark yellow), 500 (magenta) and 520 nm (red) of **Pyr2** (top) and **CuPyr2** (bottom) excited at 355 nm under inert conditions
Left: in acetonitrile
Right: in dichloromethane
The black line belongs to the fits.

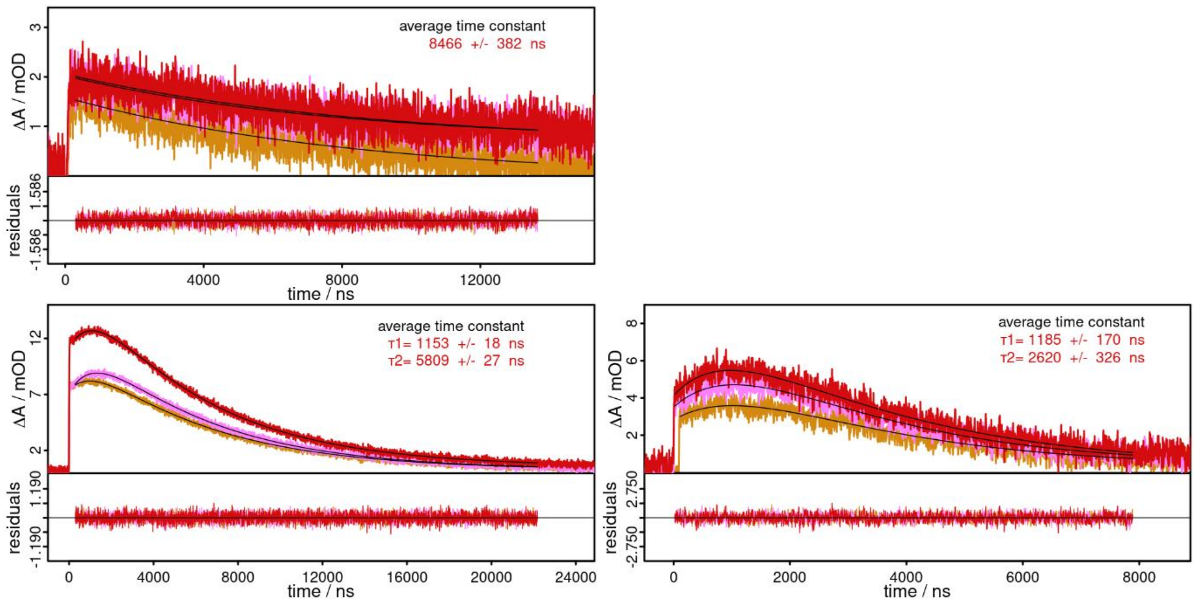

Figure S53. Single wavelength kinetic analysis at 480 (dark yellow), 500 (magenta) and 520 nm (red) of **Pyr3** (top) and **CuPyr3** (bottom) excited at 355 nm under inert conditions
Left: in acetonitrile
Right: in dichloromethane
The black line belongs to the fits.

10. Singlet Oxygen Generation

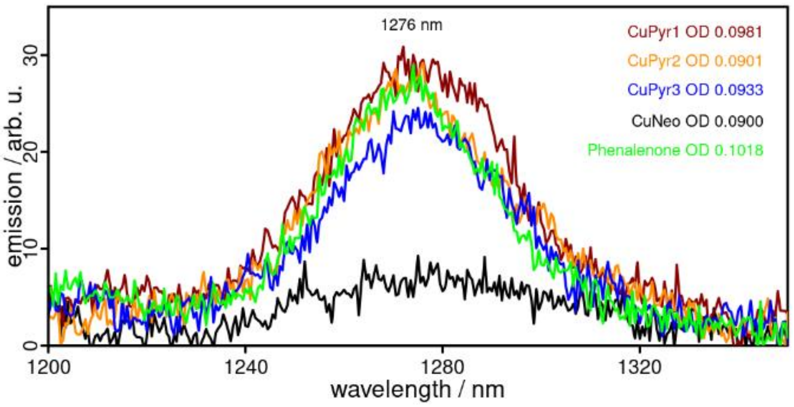

Figure S54. $^1O_2$ emission spectra after baseline correlation of phenalenone (green), **CuNeo** (black), **CuPyr1** (dark red), **CuPyr2** (orange), **CuPyr3** (blue) in aerated acetonitrile upon excitation at 407 nm with OD at 407 nm around 0.1.

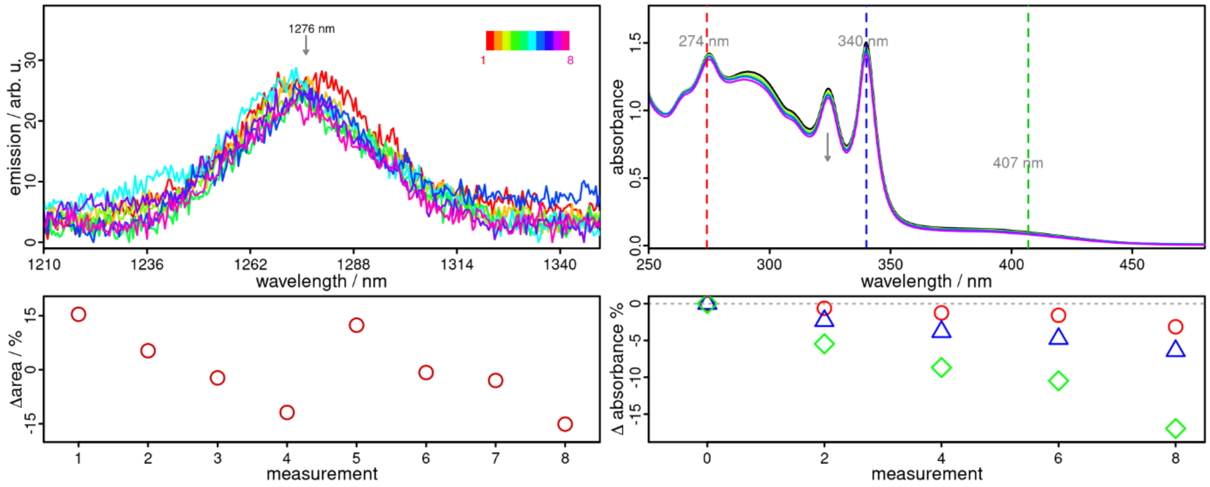

Figure S55. Near-infrared emission spectra of **CuPyr2** in aerated acetonitrile after excitation at 407 nm, showing the characteristic $^1O_2$ emission at 1276 nm. The covered cuvette was shaken vigorously three times between each measurement. Left Bottom: Relative integral differences of the recorded $^1O_2$ emission. Right Top: UV/vis absorption spectra of the same sample after every second emission measurement. Right Bottom: Relative differences in the absorbance of **CuPyr2** monitored at 270 (red), 336 (blue) and 400 nm (green), respectively.

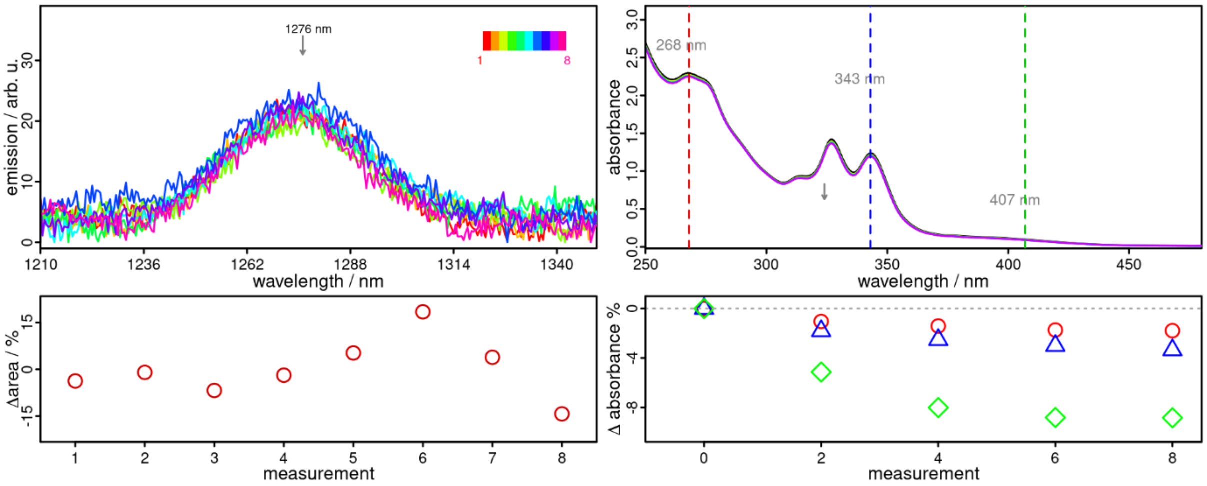

Figure S56. Near-infrared emission spectra of **CuPyr3** in aerated acetonitrile after excitation at 407 nm, showing the characteristic $^1O_2$ emission at 1276 nm. The covered cuvette was shaken vigorously three times between each measurement. Left Bottom: Relative integral differences of the recorded $^1O_2$ emission. Right Top: UV/vis absorption spectra of the same sample after every second emission measurement. Right Bottom: Relative differences in the absorbance of **CuPyr3** monitored at 270 (red), 336 (blue) and 400 nm (green), respectively.

11 Photostability

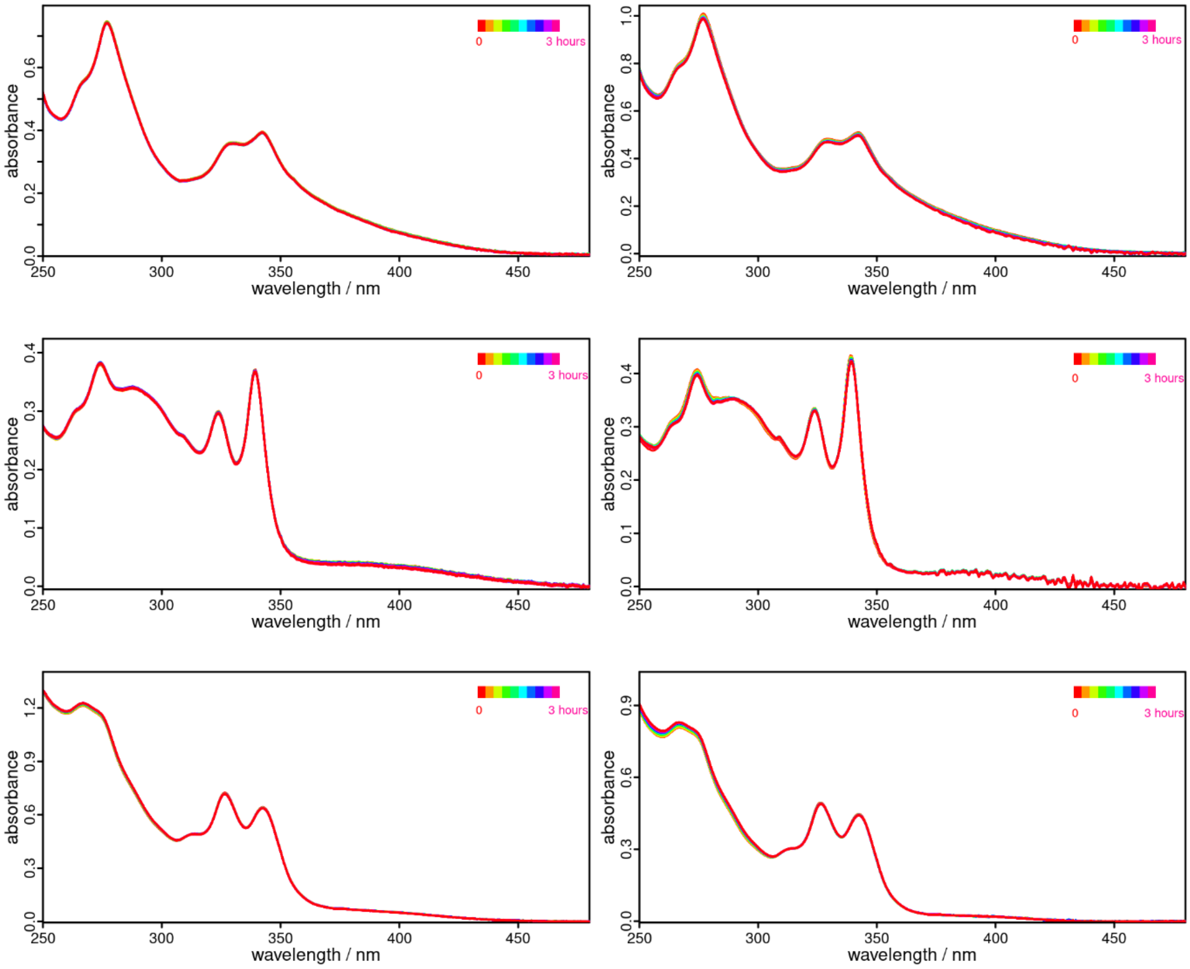

Figure S57. UV/vis absorption photostability measurements of **CuPyr1** (top), **CuPyr2** (middle) and **CuPyr3** (bottom) in oxygen free acetonitrile in the dark (left) and under irradiation (right) with 150 W Xe arc lamp for 3 hours.

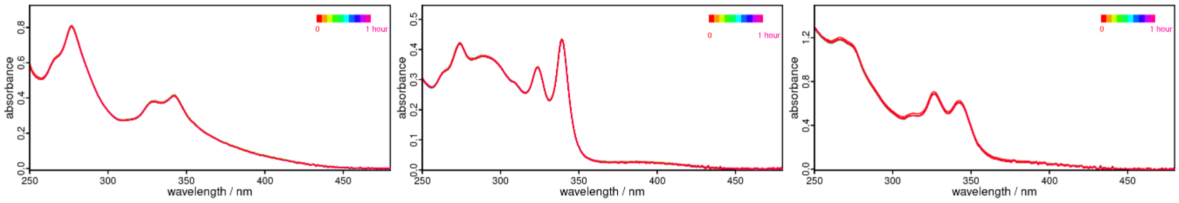

Figure S58. UV/vis absorption photostability measurements of **CuPyr1** (left), **CuPyr2** (middle), **CuPyr3** (right) in aerated acetonitrile under irradiation with 150 W Xe arc lamp for 1 hour.

12 Photooxidation of 1,5-Dihydroxynaphthalene

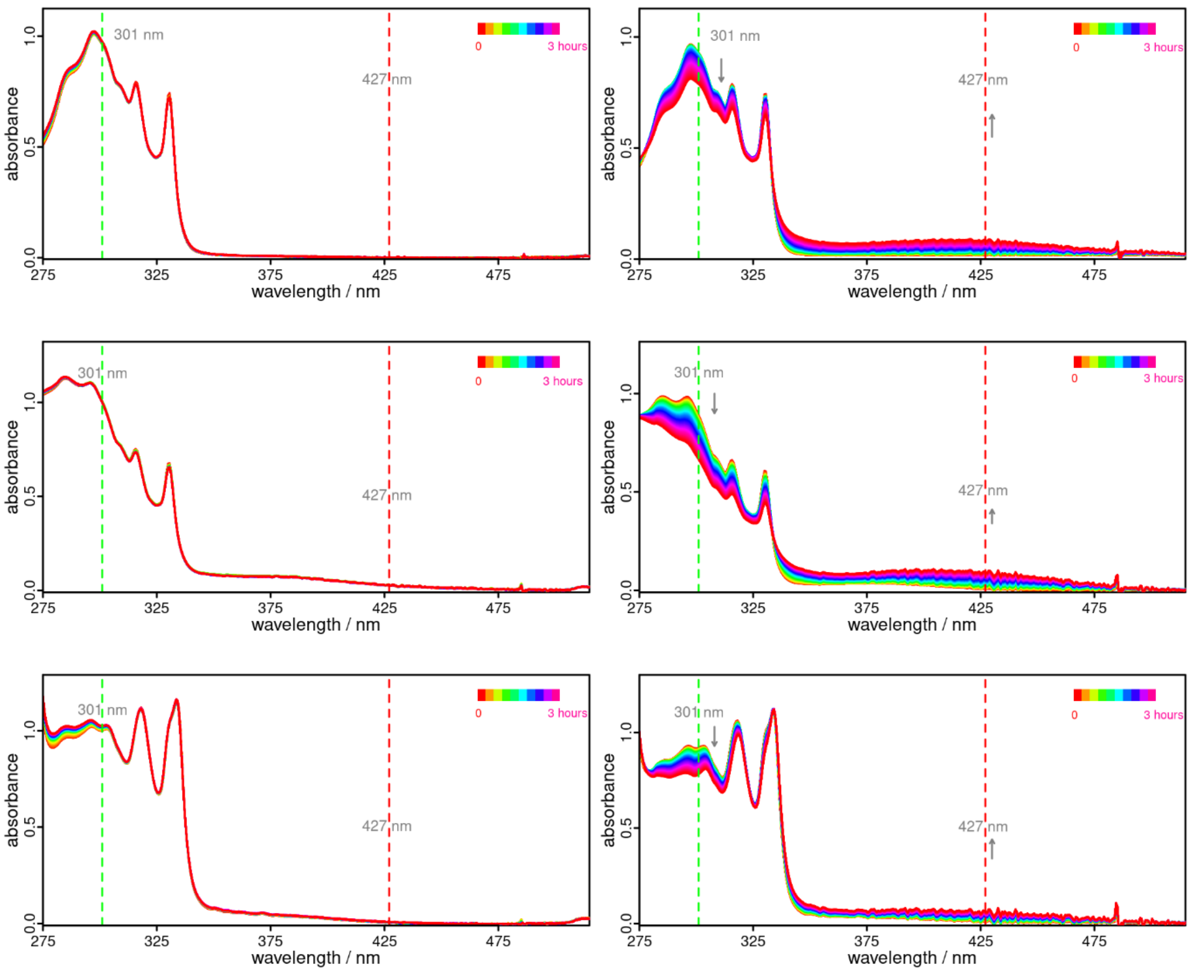

Figure S59. UV/vis absorption spectral change for the photooxidation of
Top: only DHN (c = 1.0 × 10^{-4} M),
Middle: DHN (c = 1.0 × 10^{-4} M) and **CuNeo** (c = 1.0 × 10^{-5} M) as $^1O_2$ sensitizer and
Bottom: DHN (c = 1.0 × 10^{-4} M), **CuNeo** (c = 1.0 × 10^{-5} M) and pyrene (c = 2.0 × 10^{-5} M) as $^1O_2$ sensitizer
in aerated acetonitrile in the dark (without light, left) and under irradiation (right) with a 150 W Xe arc lamp using a 380 nm long pass filter measured every 30 seconds for 3 hours.

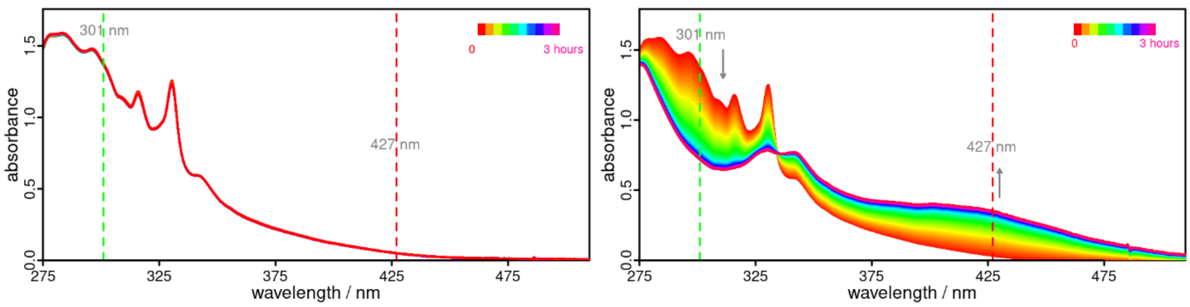

Figure S60. UV/vis absorption spectral change for the photooxidation of DHN (c = 1.0 × 10^{-4} M) and **CuPyr1** (c = 1.0 × 10^{-5} M) as $^1O_2$ sensitizer in aerated acetonitrile in the dark (without light, left) and under irradiation (right) with a 150 W Xe arc lamp and 380 nm long pass filter measured every 30 seconds for 3 hours.

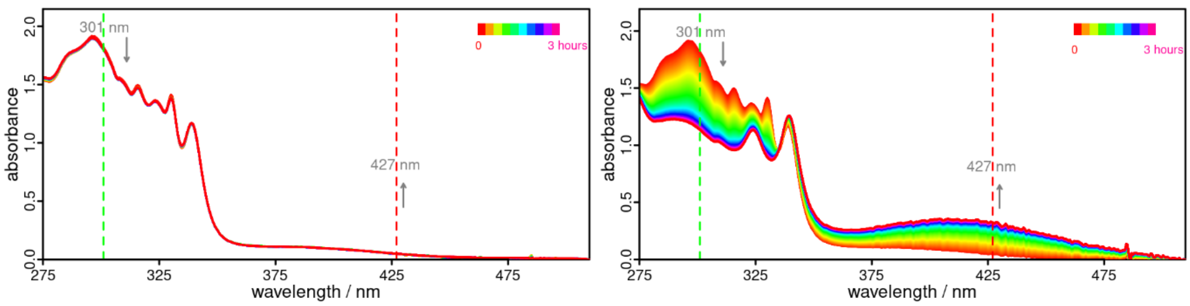

Figure S61. UV/vis absorption spectral change for the photooxidation of DHN (c = 1.0 × 10^{-4} M) and **CuPyr2** (c = 1.0 × 10^{-5} M) as $^1O_2$ sensitizer in aerated acetonitrile in the dark (without light, left) and under irradiation (right) with a 150 W Xe arc lamp and 380 nm long pass filter measured every 30 seconds for 3 hours.

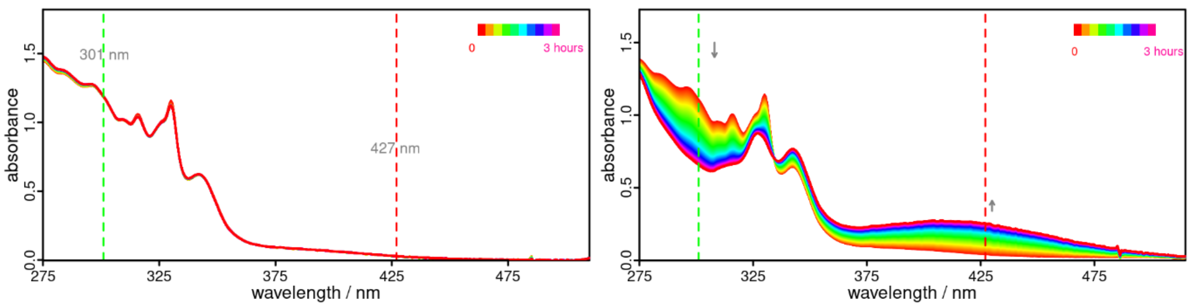

Figure S62. UV/vis absorption spectral change for the photooxidation of DHN (c = 1.0 × 10^{-4} M) and **CuPyr3** (c = 1.0 × 10^{-5} M) as $^1O_2$ sensitizer in aerated acetonitrile in the dark (without light, left) and under irradiation (right) with a 150 W Xe arc lamp and 380 nm long pass filter measured every 30 seconds for 3 hours.

References

[1] D. Karpovich, G. Blanchard, Relating the polarity-dependent fluorescence response of pyrene to vibronic coupling. Achieving a fundamental understanding of the py polarity scale, *J. Phys. Chem*. **1995**, *99*, 12, 3951–3958.

[2] J. Shirdel, A. Penzkofer, R. Procházka, Z. Shen, J. Strauss, J. Daub, Absorption and emission spectroscopic characterisation of a pyrene-flavin dyad, *Chemical Physics* **2007**, *331*, 427–437.

[3] R. Schmidt, C. Tanielian, R. Dunsbach, C. Womb, Phenalenone, a universal reference compound for the determination of quantum yields of singlet oxygen $O_2(^1\Delta_g)$ sensitization, *J. Photochem. Photobiol. A: Chem.* **1994**, *79*, 11–17.

[4] N. Epelde-Elezcano, V. Martınez-Martınez, E. Peña-Cabrera, C. Gómez-Durán, I. López Arbeloaa, S. Lacombec, Modulation of singlet oxygen generation in halogenated BODIPY dyes by substitution at their *meso* position: towards a solvent-independent standard in the vis region, *RSC Adv*. **2016**, *6*, 41991–41998.

[5] T. Gallavardin, C. Armagnat, O. Maury, P. Baldeck, M. Lindgren, C. Monnereau, C. Andraud, An improved singlet oxygen sensitizer with two-photon absorption and emission in the biological transparency window as a result of ground state symmetry-breaking, *Chem. Commun*. **2012**, *48*, 1689–1691.

[6] M.-A. Schmid, J. Brückmann, J. Bösking, D. Nauroozi, M. Karnahl, S. Rau, S. Tschierlei, Merging of a Perylene Moiety Enables a Ru^{II} Photosensitizer with Long-Lived Excited States and the Efficient Production of Singlet Oxygen, *Chem. Eur. J.* **2022**, *28*, e202103609.

[7] A. Hallett, N. White, W. Wu, X. Cui, P. Horton, S. Coles, J. Zhao, S. Pope, Enhanced photooxidation sensitizers: the first examples of cyclometalated pyrene complexes of iridium(iii), *Chem. Commun.* **2012**, *48*, 10838–10840.

[8] S. Tanaka, T. Enoki, H. Imoto, Y. Ooyama, J. Ohshita, T. Kato, K. Naka, Highly Efficient Singlet Oxygen Generation and High Oxidation Resistance Enhanced by Arsole-Polymer-based Photosensitizer: Application as a Recyclable Photooxidation Catalyst, *Macromolecules* **2020**, *53*, 2006−2013.

[9] F. Neese, F. Wennmohs, U. Becker, C. Riplinger, The ORCA quantum chemistry program package, *J. Chem. Phys.* **2020**, *152*, 224108.

[10] K. Eichkorn, O. Treutler, H. Öhm, M. Häser, R. Ahlrichs, Auxiliary basis sets to approximate Coulomb potentials, *Chem. Phys. Lett.* **1995**, *240*, 283−290.

[11] J. Perdew, K. Burke, and M. Ernzerhof, Generalized Gradient Approximation Made Simple, *Phys. Rev. Lett.* **1997**, *78*, 1396.

[12] S. Grimme, S. Ehrlich, L. Goerigk, Effect of the damping function in dispersion corrected density functional theory, *J. Comput. Chem* **2011**, *32*, 1456–1465.

[13] S. Grimme, J. Antony, S. Ehrlich and H. Krieg, A consistent and accurate *ab initio* parametrization of density functional dispersion correction (DFT-D) for the 94 elements H-Pu, *J. Chem .Phys*. **2010**, *132*, 154104.

[14] F. Weigend and R. Ahlrichs, Balanced basis sets of split valence, triple zeta valence and quadruple zeta valence quality for H to Rn: Design and assessment of accuracy, *Phys. Chem. Chem. Phys.* **2005**, *7*, 3297–3305.

[15] F. Weigend, Accurate Coulomb-fitting basis sets for H to Rn, *Phys. Chem. Chem. Phys.* **2006**, *8*, 1057–1065.

[16] V. Barone, M. Cossi, Quantum Calculation of Molecular Energies and Energy Gradients in Solution by a Conductor Solvent Model, *J. Phys. Chem. A* **1998**, *102*, 1995–2001.

[17] Chemcraft - graphical software for visualization of quantum chemistry computations. www.chemcraftprog.com
